# Novel C3/C28-bis-1,2,4-Triazolyl-sulfanylacetate-betulin Derivatives: Synthesis and Evaluation of Anticancer Potential

**DOI:** 10.3390/ijms27135960

**Published:** 2026-07-02

**Authors:** Alexandra Prodea, Marius Mioc, Andreea Munteanu, Alexandra Mioc, Nicoleta Anamaria Paşcalău, Bogdan-Ionuț Mara, Elisabeta Atyim, Mihaela Balan-Porcarasu, Roxana Racoviceanu, Codruța Șoica

**Affiliations:** 1 Faculty of Pharmacy, “Victor Babes” University of Medicine and Pharmacy, Eftimie Murgu Square, No. 2, 300041 Timisoara, Romania; alexandra.ulici@umft.ro (A.P.); marius.mioc@umft.ro (M.M.); andreea.milan@umft.ro (A.M.); alexandra.mioc@umft.ro (A.M.); mara.bogdan@umft.ro (B.-I.M.); elisabeta.atyim@umft.ro (E.A.); babuta.roxana@umft.ro (R.R.); codrutasoica@umft.ro (C.Ș.); 2 Research Center for Experimental Pharmacology and Drug Design (X-Pharm Design), “Victor Babes” University of Medicine and Pharmacy, Eftimie Murgu Square, No. 2, 300041 Timisoara, Romania; 3 Department of Psycho Neuroscience and Recovery, Faculty of Medicine and Pharmacy, University of Oradea, 410087 Oradea, Romania; 4 Coriolan Dragulescu Institute of Chemistry, Romanian Academy, Bv. M. Viteazu, No. 24, 300223 Timisoara, Romania; 5 Institute of Macromolecular Chemistry ‘Petru Poni’, 700487 Iasi, Romania; mihaela.balan@icmpp.ro

**Keywords:** pentacyclic triterpene, betulin, triazole, anticancer, bistriazole, network pharmacology, molecular docking, molecular dynamics simulation

## Abstract

The current study describes the synthesis and preliminary anticancer assessment of a novel series of C3/C28-bis-1,2,4-triazolyl-sulfanylacetate-betulin (AP1–5) derivatives to identify potent agents for clinical development. The cytotoxicity of AP1–5 was evaluated using the Alamar blue assay against MCF-7, A375, PANC-1 (cancer cells) and HaCat (human keratinocytes) cells. Moreover, the molecular mechanisms responsible for cytotoxicity were investigated through in vitro (DCFDA/H_2_DCDFA assay, caspase-3/7 assay, and morphological analysis) and in silico assays (network pharmacology, molecular docking, molecular dynamics simulation, and ADMET predictions). The result highlighted AP5, containing unsubstituted 1,2,4-triazoles, as the lead derivative of the series with increased potency against MCF-7, with an IC_50_ value of 7.41 μM compared to its phenyl-substituted analogs (AP1–4). The derivatives induced apoptosis, marked by fragmented nuclei, round cells, disorganized cytoskeletons, and activation of caspases-3/-7 through a ROS-decreasing mechanism. The network pharmacology assessment predicted AP5 may interact with key proteins in the PI3K/Akt pathway, such as MAP2K1, MDM2, IGF1, JAK2, IL2 and FGFR1, as well as ESR1, PGR and MMP2. Molecular docking suggested MMP-2 is the most favorable target for AP5 among the validated proteins, while molecular dynamics simulations supported the predicted AP5–MMP-2 interaction. Moreover, the ADMET profiling of AP5 showed acceptable intestinal absorption, non-glycoprotein-P substrate status, and reduced hepatic metabolism compared to betulin. However, the ADMET analysis also highlighted some potential toxicity risks such as DILI, genotoxicity, carcinogenicity and skin sensitization that need to be further investigated. Altogether, these promising findings support the further exploration of AP5 as a promising drug candidate for breast cancer in vivo to assess its potency and toxicity.

## 1. Introduction

Cancer remains one of the leading causes of death worldwide despite the progress made in the oncology field over the past century [1]. Among the various cancer types, breast cancer is the most commonly diagnosed malignancy in women; melanoma incidence is increasing, particularly in populations with light skin tones, while pancreatic cancer remains a highly aggressive and lethal cancer, all contributing significantly to the global cancer burden [2,3,4]. In the future, increased life expectancy and demographic growth are also expected to raise the prevalence of cancer worldwide [1]. These factors support the urgency of developing better anticancer strategies that address current limitations, such as multidrug resistance [5], bioavailability challenges [6], and treatment complexity that could lead to increased occurrence of side effects [7].

Triterpenes are natural compounds that could overcome multidrug resistance and reduce treatment complexity due to their complex structures, which can exert anticancer effects through multiple mechanisms [8], potentially reducing the number of co-administered anticancer drugs. However, their lipophilic structure leads to poor oral absorption, permeability and rapid metabolism in vivo, which limit their use in clinical practice [9]. One of the strategies used to improve their bioavailability is the chemical functionalization with heterocycles, such as triazoles, that reduce the lipophilicity of the structure, improving Absorption–Distribution–Metabolization–Elimination (ADME) parameters [10]. Moreover, they can act as bioisosteres of other functional groups [11] and increase the binding to biomolecular targets [12], potentially increasing cytotoxicity.

**Figure 1:**
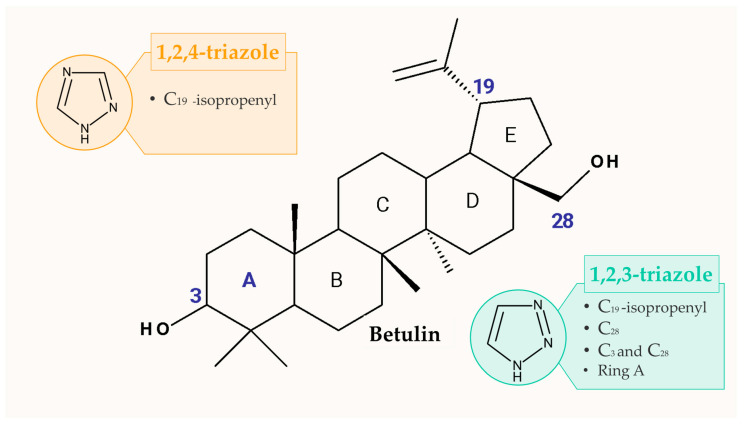
Target positions for betulin-triazole derivatives with cytotoxic effect.

Betulin (Bet) is a lupane-type pentacyclic triterpene (Figure 1) that exerts its anticancer effects by inducing cell cycle arrest, autophagy, and apoptosis via both the intrinsic and extrinsic pathways [13]. Furthermore, the bark of Betulaceae species allows the extraction of Bet in high concentrations of approximately 30 wt% [14], making it a suitable candidate for drug development.

Previously reported Bet-triazolyl derivatives, tested for their anticancer effect, were obtained by chemical derivatization in four key positions: C3 [15,16], C28 [17], C19-isopropenyl [18,19] and ring A [20,21] (Figure 1). In our previous study [18], we obtained a series of derivatives by functionalizing diacetylbetulin at the C30 position with various 1,2,4-triazololes. Upon cytotoxic evaluation, the lead compound, 3,28-O-diacetyl-30-(1*H*-1,2,4-triazole-3-yl-sulfanyl)-betulin, showed modest cytotoxicity against the A375 (melanoma) and MCF-7 (breast cancer) cell lines. Although it is not commonly used, the simultaneous functionalization of Bet with triazoles in two positions is also possible, as demonstrated by Grymel et al. [15], who used 1,2,3-triazoles as linker units to create C3, C28-glycoconjugates of Bet. These conjugates exhibited reduced cytotoxicity against MCF-7 (breast cancer) and HCT-116 (colorectal cancer) cell lines. However, when they tested a synthesis intermediate containing only 1,2,3-triazole heterocycles without sugar units, they observed increased cytotoxicity against the MCF-7 cell line compared to Bet. An increase in cytotoxicity relative to Bet was also observed in the series of Bet-1,2,3-bistriazole and monosubstituted Bet-1,2,3-triazole derivatives reported by Bębenek et al., when tested against a panel of five cell lines. Furthermore, the bistriazole derivatives demonstrated more potent cytotoxicity in breast cancer and glioblastoma cell lines than their monosubstituted analogues [16]. Considering that 1,2,3-bistriazoles derivatives showed increased cytotoxicity compared to Bet and monosubstituted analogues, we hypothesized that bi-functionalization of Bet with 1,2,4-triazoles might also influence its anticancer effect.

To validate our hypothesis, we employed five previously synthesized 5-substituted-1,2,4-triazoles [18,22] to obtain a series of C3/C28-bis-1,2,4-triazolyl-sulfanylacetate-Bet derivatives (AP1–5), which were further evaluated in vitro against HaCaT (human keratinocytes), MCF-7 (breast cancer), A375 (melanoma) and PANC-1 (pancreatic cancer) cell lines, selected as an initial screening panel to assess the potential cytotoxicity of AP1–5. Moreover, the mechanisms underlying their biological activity were investigated through immunofluorescence analysis, as well as by assessing caspase-3/-7 activation and reactive oxygen species (ROS) generation. To gain further insights into the potential anticancer molecular mechanism, an integrated in silico approach combining network pharmacology, molecular docking, and molecular dynamics simulation was employed.

## 2. Results and Discussions

### 2.1. Chemistry

**Figure 2:**
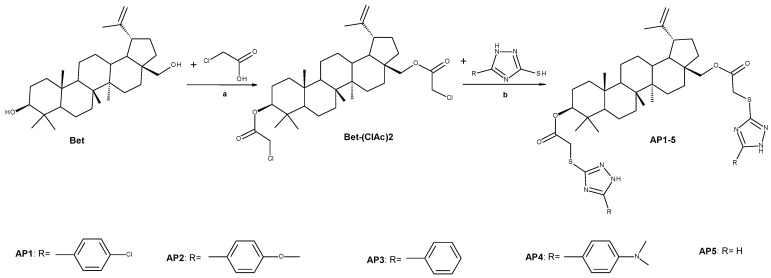
Synthesis of C3/C28-bis-1,2,4-triazolyl-sulfanylacetate-Bet derivatives. Bet (betulin); Bet-(ClAc)2 (betulin-3,28-O-di(chloroacetate)); AP1 (betulin-3,28-O-diyl-bis[5-(4-chlorophenyl)-1*H*-1,2,4-triazol-3-yl)sulfanylacetate]); AP2 (betulin-3,28-O-diyl-bis[5-(4-methoxyphenyl)-1*H*-1,2,4-triazol-3-yl)sulfanylacetate]); AP3 (betulin-3,28-O-diyl-bis[5-(phenyl)-1*H*-1,2,4-triazol-3-yl)sulfanylacetate]); AP4 (betulin-3,28-O-diyl-bis[5-(4-dimethylaminophenyl)-1*H*-1,2,4-triazol-3-yl)sulfanylacetate]); AP5 (betulin-3,28-O-diyl-bis[5-(1*H*-1,2,4-triazol-3-yl)sulfanylacetate]). Reaction conditions: a. DCM, DCC, DMAP, rt, 24 h, inert atmosphere; b. K_2_CO_3_, DMF, rt, 48 h.

The compounds AP1–5 were obtained through a two-step protocol depicted in Figure 2. The first step, a Steglich-type esterification, was performed according to a minorly adjusted protocol from the literature [19,23]. The resulting intermediate, betulin-3,28-O-di(chloroacetate) (Bet-(ClAc)2), was obtained without noticeable impurities, revealed by TLC, and was therefore used in all reactions without further purification. In the subsequent step, Bet-(ClAc)2 underwent alkylation [24] with previously synthesized 5-substituted-1,2,4-triazole-thiols [18] to form the final derivatives (AP1–5) in moderate yields (30–38%). The AP1–5 derivatives were also purified through column chromatography using various ratios of hexane:ethyl acetate (1:1, 2:1) as eluent. The structures of AP1–5 were confirmed using ^1^H, ^13^C NMR, and FTIR spectroscopy. All spectral data are contained within the Supplementary Material File (Figures S1–S18).

The ^1^H NMR spectrum of Bet-(ClAc)2 shows the peaks for the betulin scaffold in the 4.7–0.7 ppm region and two additional peaks at 4.08 and 4.05 ppm, each corresponding to two protons from the two newly attached chloromethylenic groups. The ^13^C NMR spectrum shows the peaks for the betulinic carbons at the expected chemical shifts, the peaks for the esteric C31 and C33 carbons at 167 ppm and the peaks for the chloromethylenic carbons, C32 and C34, at 41 ppm. The NMR spectra of the AP1–AP5 derivatives are in agreement with the proposed structures and show the peaks for the betulinic protons and for the triazole derivative substituents in the appropriate integral ratios and at the expected chemical shifts. When analyzing the aromatic region of the ^1^H NMR spectra for AP1, AP3 and AP5, we observed that some of the peaks were doubled and broadened, but if integrated together, the integral values were in appropriate ratios compared to the protons from Bet. This behavior was also observed in diacetylbetulin derivatives containing 5-substituted-1,2,4-triazoles at C30 [18] and was attributed to the existence of a slow exchange tautomeric equilibrium that can occur in 3,5-disubstituted 1,2,4-triazoles [25,26]. To prove beyond any doubt that the aspect of the NMR spectra is due to the existence of tautomeric equilibrium, to each solution of AP1, AP3 and AP5 in DMSO-d_6_, for which the initial experiments were recorded, we added a drop of trifluoroacetic acid (TFA) and then recorded all the NMR experiments again. Adding TFA sped up the tautomeric equilibrium, leading to a fast exchange system, and the NMR spectra show only one set of peaks for the triazole derivatives, in appropriate integral ratios compared to the protons from Bet.

### 2.2. Biological Assessment

#### 2.2.1. The Evaluation of AP1–5 on Cell Viability

The cytotoxic effect against human keratinocytes HaCaT, melanoma A375, breast cancer MCF-7 and pancreatic PANC-1 cancer cells was assessed 48 h post-treatment with AP1–5 (1, 5, 10, 50 and 100 μM) by means of the Alamar blue assay. When tested against HaCaT cells, it was shown that only the highest tested concentration (100 μM) of each compound induced low cytotoxic effects against human keratinocytes, these effects not being observed at other concentrations (Supplementary File, Figure S19). These show that AP1–5 are less cytotoxic than Bet against HaCaT, with a previously reported IC_50_ value of 60.75 μM [18], suggesting the derivatization decreased the cytotoxicity on non-cancer cells and may lead to a more favorable in vitro selectivity profile.

In terms of anticancer activity, AP5, containing two unsubstituted 1,2,4-triazole units, exhibited potent cytotoxic effects against all cancer cell lines tested, with IC_50_ values (μM) reported in Table 1. These suggest increased cytotoxicity compared to previously reported IC_50_ values for Bet of 37.29 μM in MCF-7 and 46.19 μM in A375 [18]. Additionally, a similar bis(hydroxymethyl-1,2,3-triazolyl)-Bet derivative reduced the proliferation of MCF-7 up to 20% at both tested concentrations (25–50 μM), though the IC_50_ value has not been determined [15], suggesting bis-triazole derivatives of Bet have anticancer potential in breast cancer models. However, the AP1–4 derivatives, containing phenyl-substituted triazoles, were essentially inactive with IC_50_ values greater than 100 μM. By contrast, similar 3,28-bis-1,2,3-triazole-Bet derivatives, where the triazoles were substituted with 4-fluorobenzyl and 4-(methylthio)benzyl, exerted marked cytotoxicity (IC_50_ < 1 μM) in MCF-7 [16], highlighting that minor structural differences of triazole substituents can drastically change the anticancer effect. When compared with the standard drug, doxorubicin, AP5 exerted superior cytotoxicity in the PANC-1 cell line, but lower cytotoxicity in MCF-7 and A375 cell lines (Table 1). Additionally, the lower cytotoxicity of AP5 compared to doxorubicin on the HaCaT cell line (Table 1) suggests that AP5 might exert a more selective effect towards cancerous compared to non-cancerous cells, an aspect that should be further studied.

Nevertheless, AP1–4 exhibited cytotoxic effects against MCF-7, A375 and PANC-1 cell lines in a dose- and time-dependent manner. In terms of their anticancer activity against breast cancer, the compounds proved the strongest cytotoxicity when the highest concentrations were applied as follows: 71.27 ± 2.82 (100 μM) and 78.21 ± 4.32 (50 μM) for AP1, 72.28 ± 3.33 (100 μM) and 74.76 ± 4.68 (50 μM) for AP2, 69.43 ± 2.13 (100 μM) and 75.14 ± 3.73 (50 μM) for AP3, 69.17 ± 3.83 (100 μM) and 74.72 ± 3.69 (50 μM) for AP4 (Supplementary File, Figure S20).

The incubation of A375 melanoma cells with the highest tested concentrations of each compound (48 h) recorded a viability inhibition after treatment of: 71.47 ± 2.43 (100 μM), 80.04 ± 2.26 (50 μM) for AP1, 78.52 ± 3.88 (100 μM), 84.03 ± 2.36 (50 μM) for AP2, 74.85 ± 3.97 (100 μM), 82.35 ± 4.7 (50 μM) for AP3, 82.47 ± 4.96 (100 μM), 85.1 ± 5.1 (50 μM) for AP4 (Supplementary File, Figure S21).

When tested against PANC-1 cells, the compounds inhibited cell proliferation in a dose-dependent manner, only the highest tested concentrations showing cytotoxic activity, as follows: 74.35 ± 2.31 (100 μM), 78.82 ± 2.37 (50 μM) for AP1, 74.29 ± 4.76 (100 μM), 77.53 ± 5.61 (50 μM) for AP2, 72.17 ± 3.36 (100 μM), 75.22 ± 6.93 (50 μM) for AP3, 70.58 ± 4.63 (100 μM), 71.55 ± 4.99 (50 μM) for AP4 (Supplementary File, Figure S22).

#### 2.2.2. Morphological Changes of Cell Nuclei and Cytoskeleton—Immunofluorescence Assay

Apoptosis is a cell death mechanism characterized by cellular hallmarks, such as blebbing, cell shrinkage, DNA and nuclear fragmentation [27,28]. Additionally, following cell shrinkage, the cells detach from neighboring cells and the extracellular matrix, adopting a more rounded morphology due to the reorganization of focal adhesions [29]. The 48 h incubation of AP1–5 with non-malignant HaCaT cells showed no significant difference in terms of cellular morphology, only displaying a slightly lower number of cells compared to the control group. However, against MCF-7, A375 and PANC-1 cancer cells, all compounds induced an increase in overall cell reduction, as well as morphological changes such as detached and round cells (Supplementary File, Figures S23–S26).

**Figure 3:**
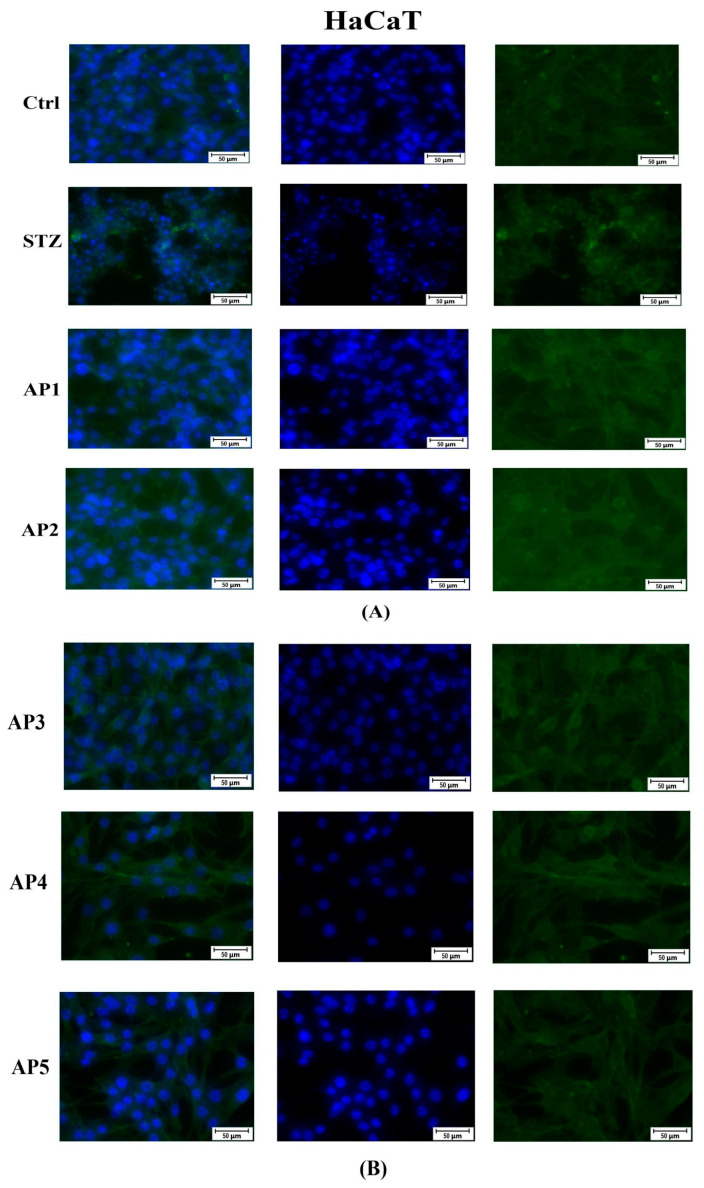
The effects of 48 h treatment with AP1, AP2 (100 μM) (**A**), AP3, AP4 and AP5 (100 μM) (**B**) on HaCaT cell nuclei (second column—blue—Hoechst staining), cytoskeleton (third column—green—beta-actin) and the merged picture (first column). Staurosporine (STZ, 5 μM) was used as a positive control for necrotic cell death. The scale bar is 50 μm. Enlarged versions of panels (**A**,**B**) are provided in the Supplementary File (Figures S27 and S28).

**Figure 4:**
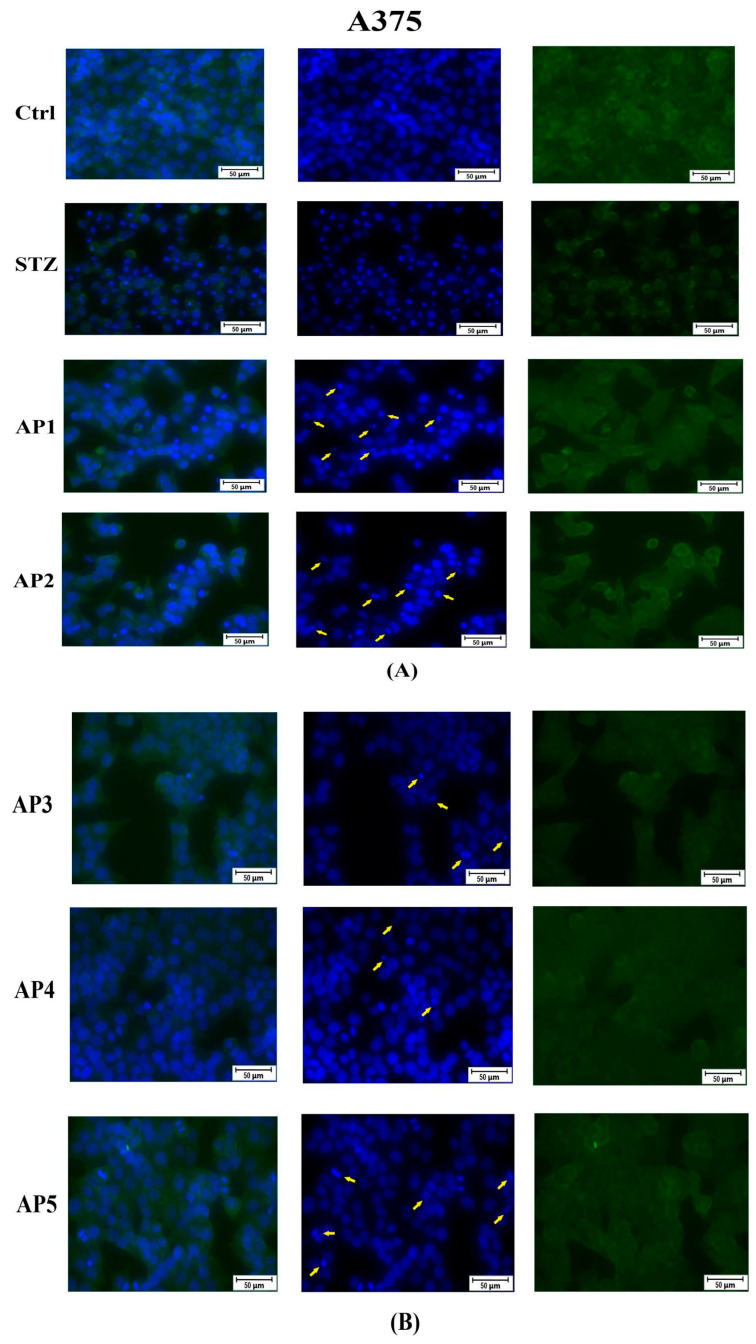
The effects of 48 h treatment with AP1, AP2 (100 μM) (**A**), AP3, AP4 and AP5 (100 μM/IC_50_) (**B**) on A375 cell nuclei (second column—blue—Hoechst staining), cytoskeleton (third column—green—beta-actin), and the merged picture (first column). Staurosporine (STZ, 5 μM) was used as a positive control for necrotic cell death. The scale bar is 50 μm. The yellow arrows indicate morphological features consistent with apoptosis. Enlarged versions of panels (**A**,**B**) are provided in the Supplementary File (Figures S29 and S30).

**Figure 5:**
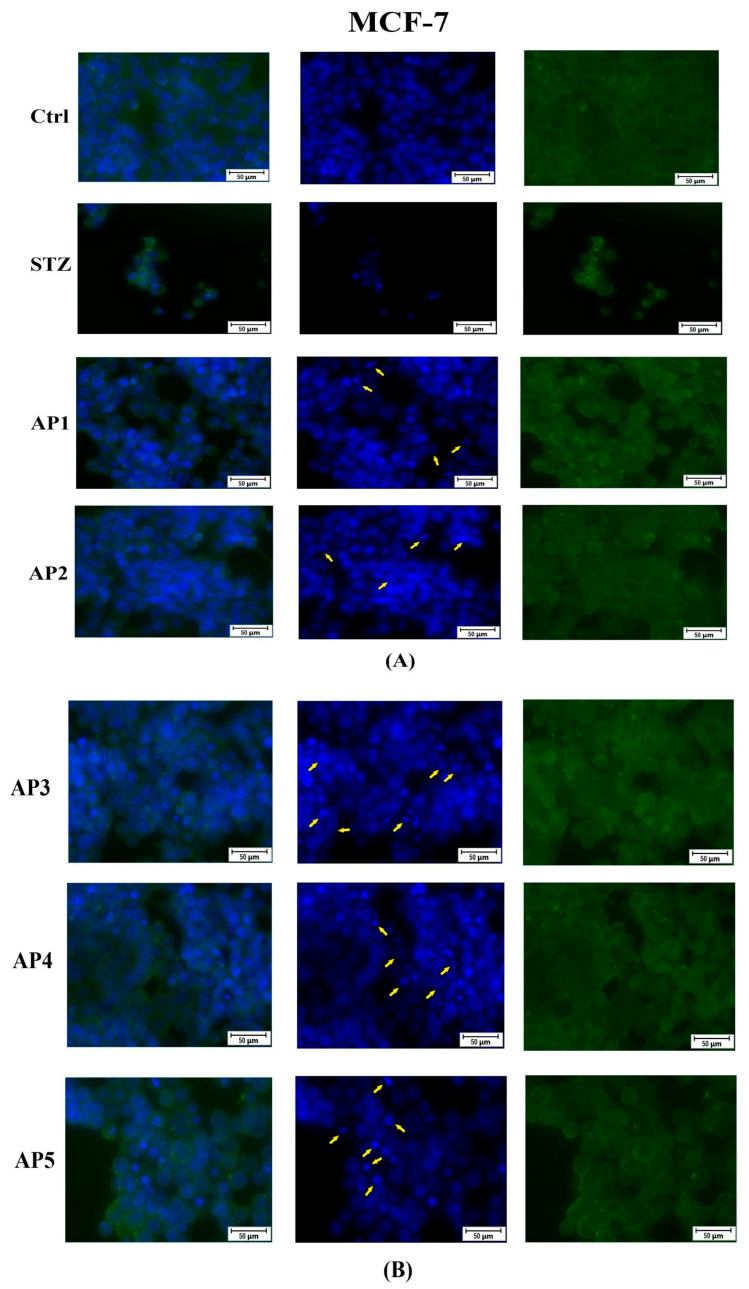
The effects of 48 h treatment with AP1, AP2 (100 μM) (**A**), AP3, AP4 and AP5 (100 μM/IC_50_) (**B**) on MCF-7 cell nuclei (second column—blue—Hoechst staining), cytoskeleton (third column—green—beta-actin) and the merged picture (first column). Staurosporine (STZ, 5 μM) was used as a positive control for necrotic cell death. The scale bar is 50 μm. The yellow arrows indicate morphological features consistent with apoptosis. Enlarged versions of panels (**A**,**B**) are provided in the Supplementary File (Figures S31 and S32).

**Figure 6:**
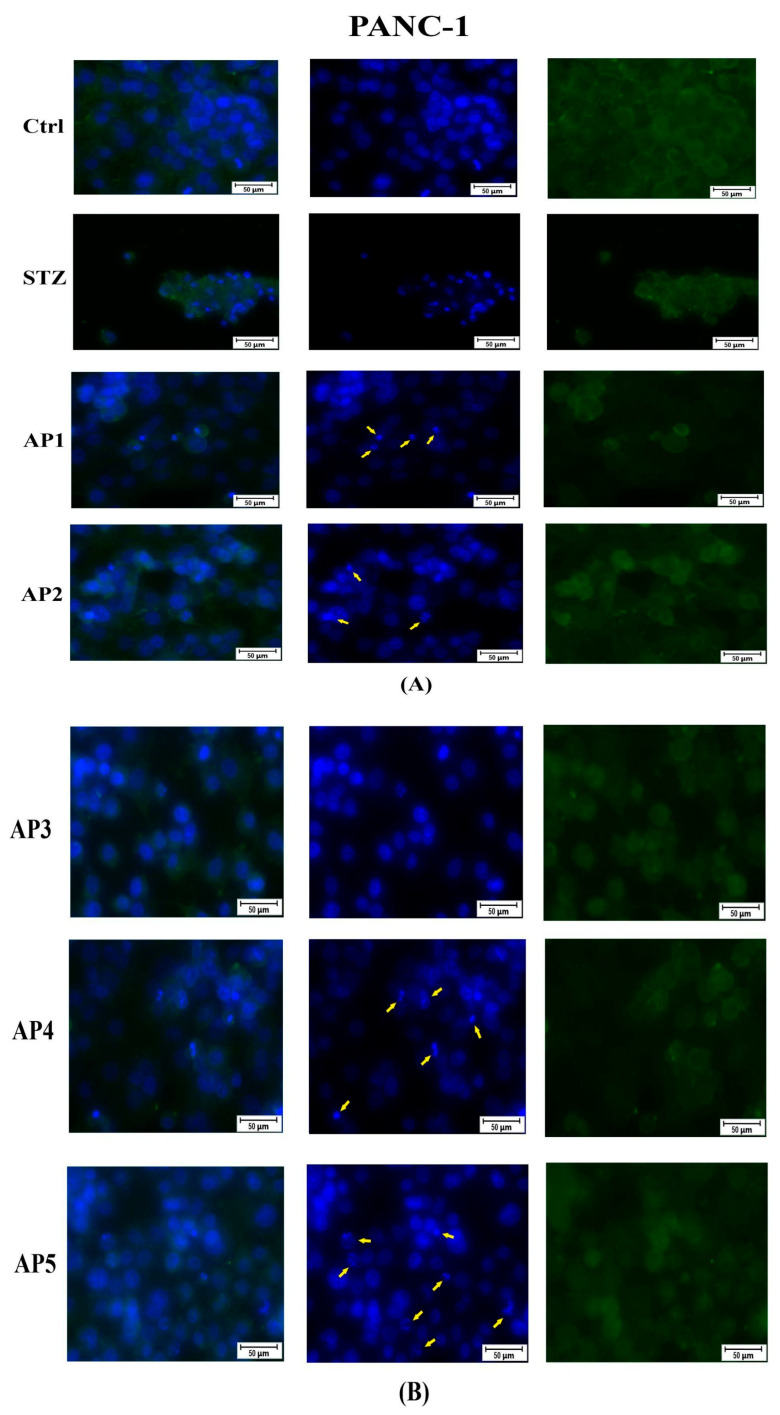
The effects of 48 h treatment with AP1, AP2 (100 μM) (**A**), AP3, AP4 and AP5 (100 μM/IC_50_) (**B**) on PANC-1 cell nuclei (second column—blue—Hoechst staining), cytoskeleton (third column—green—beta-actin) and the merged picture (first column). Staurosporine (STZ, 5 μM) was used as a positive control for necrotic cell death. The scale bar is 50 μm. The yellow arrows indicate morphological features consistent with apoptosis. Enlarged versions of panels (**A**,**B**) are provided in the Supplementary File (Figures S33 and S34).

To evaluate the cytotoxic effects recorded in A375, MCF-7 and PANC-1 cells, as well as in non-malignant HaCaT, the cells’ nuclei were stained with Hoechst solution, while their cytoskeleton was labelled with beta-actin 48 h post-treatment with AP1–5. Morphological features consistent with apoptosis were observed in all tested cancer cell lines, including small, bright, and fragmented nuclei, round cells, and disorganized cytoskeletons. Furthermore, in HaCaT cells, no significant changes were detected in terms of nuclei and cytoskeleton architecture after the highest tested concentration (100 μM) was applied (Figure 3, Figure 4, Figure 5 and Figure 6), suggesting a degree of selectivity of AP1–5 towards cancerous cells under the experimental conditions used.

#### 2.2.3. The Effects of AP1–5 on Caspase-3/-7 Production

**Figure 7:**
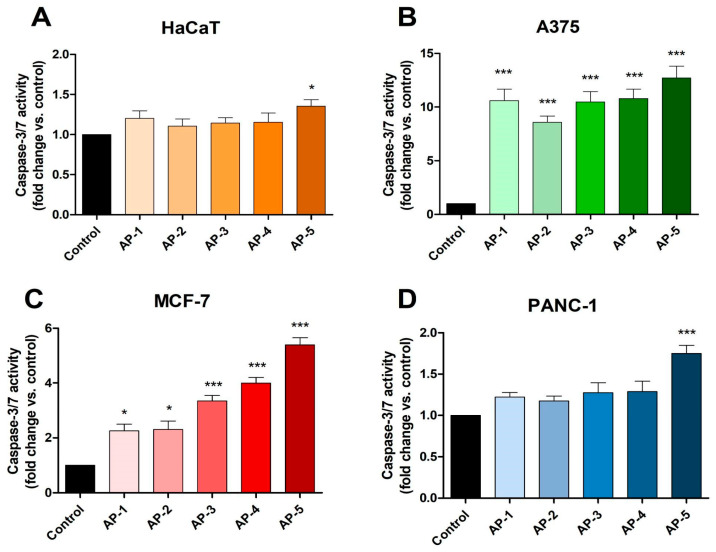
The activation of caspase-3/7 in HaCaT (**A**), A375 (**B**), MCF-7 (**C**), and PANC-1 (**D**) cell lines after 24 h of treatment with AP1–5 (IC_50_/100 μM). The experiments were performed in triplicate, and results were calculated as mean ± SD; *n* = 3 per group. * *p* < 0.05, and *** *p* < 0.001.

Previous studies have shown that Bet and similar pentacyclic triterpenes induce apoptosis mainly through the intrinsic pathway [8,28]. Caspases are key enzymes in apoptosis and can be divided into initiator caspases, such as caspase-8 and -9, and executioner caspases, such as caspase-3 and -7 [30]. To further investigate the pro-apoptotic potential of the tested compounds, HaCaT, A375, MCF-7 and PANC-1 cells were treated with AP1–5 (IC_50_/100 μM) for 24 h; following the treatment period, caspase-3/7 activity was quantitatively evaluated using the Caspase-3/7 Green Detection Reagent kit and an automated fluorescence cell counter. The results show that treatment of A375 and MCF-7 cells with AP1–5 induced significant increases in caspase-3/7 activity compared to untreated controls (Figure 7). In contrast, in HaCaT and PANC-1 cell lines, only AP5 significantly increased caspase-3/7 activity compared with control (Figure 7). For comparison, a C-28 triazole-Bet derivative reduced the expression of procaspase-3 in HL-60 cells (leukemia) in a dose-dependent manner, indirectly suggesting the formation of cleaved caspase-3 [31]. Moreover, the increased expression of caspase-3 was also shown in SK-BR-3 cells (breast cancer) for 30-diethoxyphosphoryl-28-propynoyl-Bet [32], supporting the ability of Bet derivatives to induce apoptosis.

Notably, as the MCF-7 cell line lacks functional caspase-3 [33], the increased expression of caspases observed in our assay can be attributed solely to caspase-7, which promotes the detachment of apoptotic cells from the extracellular matrix [34], an effect confirmed through the morphological assessment of MCF-7 in the presence of AP1–5 (Supplementary File, Figure S25).

#### 2.2.4. The Effects of AP1–5 Derivatives on ROS Production

**Figure 8:**
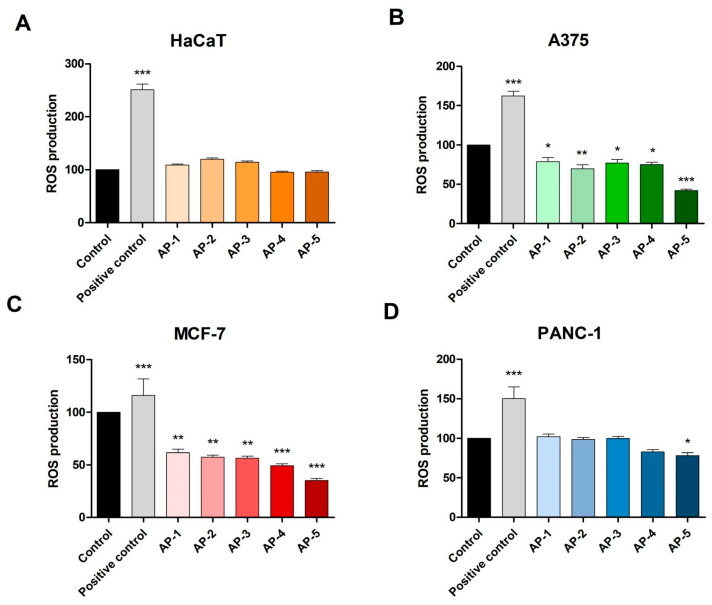
The effect of 24 h of treatment with AP1–5 (IC_50_/100 μM) on ROS production in HaCaT (**A**), A375 (**B**), MCF-7 (**C**), and PANC-1 (**D**) cell lines. The results are expressed as mean ± SD; *n* = 3 per group. * *p* < 0.05, ** *p* < 0.01, and *** *p* < 0.001.

The role of ROS in cancer treatment remains controversial, as ROS are involved both in carcinogenesis and apoptosis induction through intrinsic and extrinsic pathways [35,36]. To investigate whether the observed effects of AP1–5 are associated with the modulation of ROS levels, HaCaT, A375, MCF-7 and PANC-1 cell lines were treated for 24 h with AP1–5 (IC_50_/100 μM) and assessed using the DCFDA/H_2_DCFDA assay. Tert-butyl hydroperoxide (TBHP) was used as a positive control (Figure 8). The results showed that ROS production in HaCat cells was not influenced by AP1–5 treatment. In contrast, all the tested compounds significantly decreased ROS production in A375 and MCF-7 cell lines compared to the control. The most notable decrease in ROS production was recorded after AP5 treatment in A375 (41.96% ± 2.7) and MCF-7 (35.19% ± 4.2) cell lines vs. control (100%), while in the PANC-1 cell line, only AP5 decreased ROS production (78.11% ± 7.3 vs. control) (Figure 8). Collectively, these findings suggest that AP1–5 exert an antioxidant effect in selected cell lines, aligning with a previous report of a Bet derivative showing a cell-type-dependent ROS modulation, increasing ROS in MCF-7 and decreasing ROS in the SK-BR-3 cell line while still inducing cell death [32], thereby suggesting that modulation of ROS levels by AP1–5 may contribute to the observed cytotoxicity.

### 2.3. Network Pharmacology

Network pharmacology is a bioinformatic approach used to explore molecular targets and subsequent mechanisms of action responsible for a pharmacological effect, widely used in the study of natural compounds, such as Bet and other triterpenes, due to their innate polypharmacological nature [37,38,39]. This approach was used to predict possible mechanisms responsible for the cytotoxicity of Bet and AP1–5 in melanoma, breast, and pancreatic cancer.

#### 2.3.1. Target Identification

PharmaMapper was used to identify potential targets for Bet and AP1–5, which were further refined based on dual criteria, fit score (>3) and z’-score (>1) (Table 2), thresholds used in similar studies of natural compounds [40,41]. The fit score threshold ensures the compound and target binding sites have good geometric compatibility, while the z’-score threshold ensures the compound–target interactions are statistically relevant [42]. Among the derivatives, AP5 presented 104 potential targets, a significant increase compared to the other compounds (Bet and AP1–4). The curated lists of potential targets for Bet and AP1–5 are provided in the Supplementary File (Table S1).

**Figure 9:**
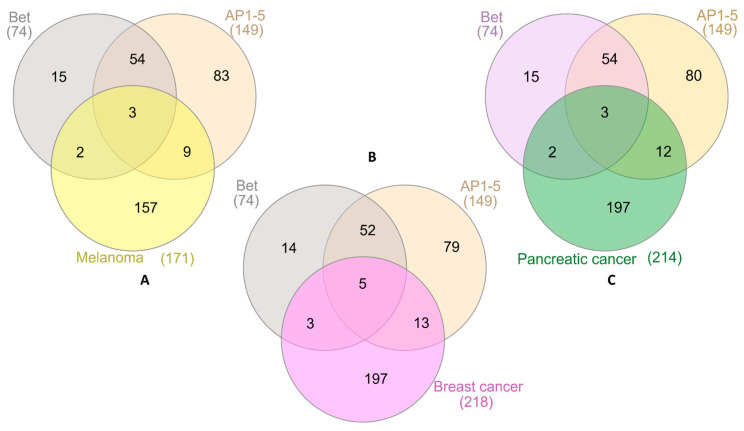
Venn diagrams illustrating the overlap of predicted protein targets among Bet, AP1–5, and cancer-specific gene sets across melanoma (**A**), breast cancer (**B**) and pancreatic cancer (**C**). Target sets for AP1–AP5 were combined, and duplicates were removed based on UniProt ID before overlap analysis. Overlapping regions represent common target sets, and total target counts per set are indicated in parentheses.

To visualize and identify common targets between our compounds (Bet and AP1–5) and relevant targets in melanoma, breast, and pancreatic cancer, three Venn diagrams were constructed (Figure 9). To create the compound–target sets, the predicted protein targets of AP1–5 were pooled into a single dataset by eliminating duplicate targets. The intersection with cancer-specific targets for melanoma, breast, and pancreatic cancer revealed that AP5 had the highest number of target overlaps across all cancer types, suggesting that the in vitro observed cytotoxicity may result from a polypharmacological mechanism. Moreover, the results suggest that although AP1–5 share some targets with the parent compound, Bet, the triazole functionalization can increase the number of targets reached. For instance, in breast cancer, while Bet and AP1–5 share five common targets, namely PGR, IL2, MDM2, PIK3R1 and CHEK1, the triazole functionalization increased the number of targets with 13 additional proteins, namely EGFR, ESR1, FRFR1, MET, MMP2, MAP2K1, IGF1, BRAF, JAK2, ERBB4, SRC, TGFBR1, and AURKA (Supplementary Material, Table S3). However, functionalization might nullify the effect on AR and PPARG (Figure 9, Supplementary File: Table S3). The shared targets predicted by PharmaMapper between each compound (Bet and AP1–5) and at least one cancer-specific target were further analyzed. The curated lists of protein targets for each cancer type, along with the common targets identified between these malignancies and the compounds, are provided in the Supplementary Material (Tables S2 and S3).

#### 2.3.2. Global Pathway Enrichment Analysis (Metascape)

**Figure 10:**
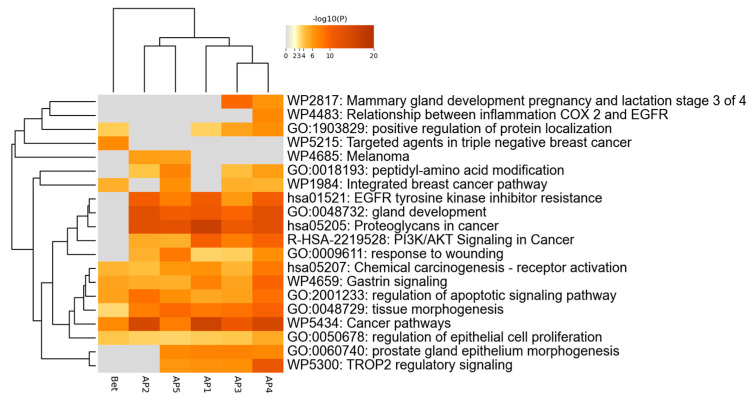
Metascape pathway enrichment heatmap of predicted targets for Bet and AP1–5.

To explore if our previously identified targets interact in the human organism, we employed a global pathway enrichment analysis using Metascape, highlighting the top 20 pathways based on their statistical significance (Figure 10). The heatmap shows biological pathways linked to the predicted targets for Bet and AP1–5, with darker orange indicating stronger statistical enrichment and greater predicted mechanistic significance [43]. The results show that the potential targets for AP1–5 are involved in general cancer pathways, including cancer pathways, proteoglycans in cancer, and chemical carcinogenesis (Figure 10). Moreover, specific cancer pathways for proliferation (regulation of epithelial cell proliferation, TROP2 regulatory signaling) and apoptosis (regulation of apoptotic signaling pathway) are also enriched (Figure 10). Notably, the enrichment analysis also highlighted the significance of P13K/Akt signaling in cancer and EGFR tyrosine kinase inhibitor resistance, pathways involved in both proliferation and apoptosis [44]. The enrichment of melanoma and breast cancer-related pathways, such as targeted agents in triple-negative breast cancer and the integrated breast cancer pathway, was also observed, supporting the potential involvement in the anticancer effect observed in MCF-7 and A375 cell lines. The analysis also revealed that AP1–5 could potentially have a broader effect on cancer pathways compared to Bet, indicating that triazole functionalization can enhance anticancer effects. However, the cytotoxicity assay showed superior cytotoxicity only for AP5 compared to Bet on MCF-7 and A375 [18], indicating that while triazole substitution of Bet is beneficial, the triazole substituents have a direct effect on target affinity.

#### 2.3.3. Network Construction and Hub Target Identification (Cytoscape, CytoHubba)

**Figure 11:**
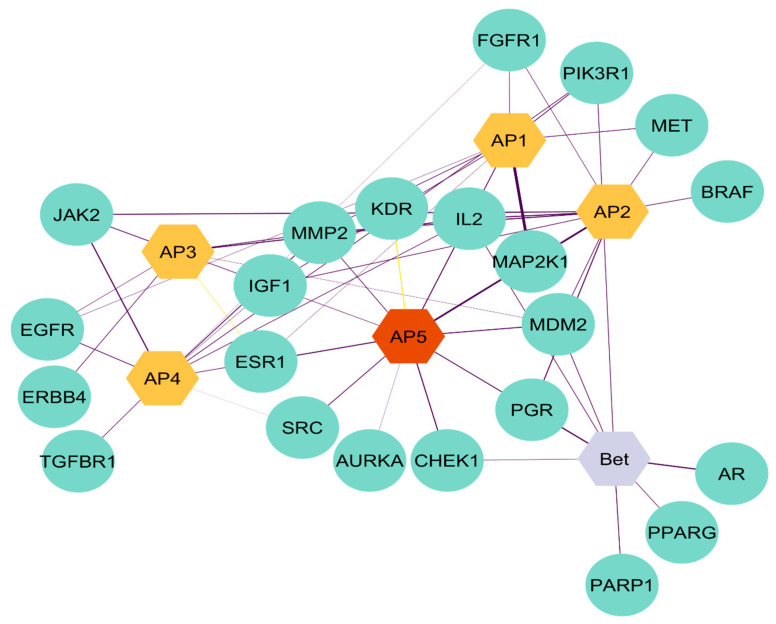
Compound–target interaction network for Bet and AP1–5 (radial layout). Hexagons represent the compounds, whereas ellipses represent protein targets. The lead derivative (AP5) is shown as a red hexagon, the parent compound as a light purple hexagon (Bet) and its other derivatives as yellow hexagons. Edges depict the predicted compound–target interactions, with edge width proportional to the PharmMapper fit score and edge color ranging from yellow to violet according to the GeneCards relevance score for breast cancer.

**Figure 12:**
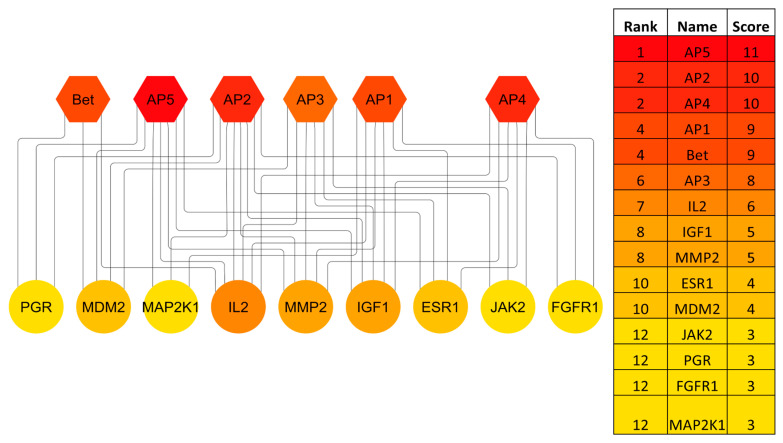
CytoHubba ranking of the top 15 nodes in the compound–target network (degree ranking, yfiles hierarchical layout). Nodes with equal score values were assigned the same rank, and the subsequent rank was skipped according to standard competition ranking.

To visualize and topologically characterize the interactions between our compounds (Bet and AP1–5) and the identified targets, a compound–target network with 28 nodes and 56 edges was constructed using Cytoscape (Figure 11). Network edges were colored from yellow to dark violet in accordance with the breast cancer relevance score (Supplementary Material: Table S2), as breast cancer showed the highest target overlap with our compounds (Figure 9), and the greatest in vitro sensitivity to AP5, our lead derivative. The network topology highlighted the central importance of AP5 (Figure 11), results consistent with its superior cytotoxicity observed against all tested cell lines, in particular against MCF-7. The results are also supported by the cytoHubba ranking, which illustrates, in a color gradient manner, from red to yellow, the most influential nodes, placing AP5 first among the top 15 nodes in the network (Figure 12). This shows AP5 possesses a high degree of connectivity with the targets, suggesting that the superior cytotoxicity observed in vitro might arise from a multitarget mechanism of action. Besides the six compounds (Bet and AP1–5), the ranking analysis identified nine hub targets, namely IL2, IGF1, MMP2, ESR1, MDM2, JAK2, PGR, FGFR1 and MAP2K1, that have different relevance in the types of cancer investigated. In breast cancer, IGF, FGFR and ESR act as upstream regulators of the PI3K/Akt and MAPK signaling pathways, associated with proliferation and survival mechanisms [45,46]. Moreover, although progesterone was primarily used as a marker for ESR activity in breast cancer models, recent studies suggest its role is more complex, acting as a binding partner able to modify ESR activity [47]. Pentacyclic triterpenes such as dimethyl melaleucate and ursolic acid have inhibited PI3K/Akt signaling in MCF-7 cells [48,49].

In melanoma, the PI3K/Akt and MAPK signaling are associated with invasion, proliferation and survival mechanisms [50,51]. Other pentacyclic triterpenes, such as oleanolic and ursolic acids, have downregulated these pathways in melanoma models [52]. Additionally, IL2 has also been shown to enhance immune response in metastatic melanoma, for which it has gained FDA approval [53]. However, its mechanism in melanoma is not fully understood, and clinical response can vary significantly among patients. Moreover, high doses of IL2 can be associated with life-threatening side effects, such as the capillary leak syndrome [54]. While the immunomodulatory properties of pentacyclic triterpenes have been reported in the literature, the effect on IL2 is rarely quantified, as shown for 3β,6β-Dihydroxyolean-12-en-27-oic acid, which increased IL2 in a murine sarcoma model [55,56].

Additionally, the involvement of PI3K/Akt, MAPK, and JAK/STAT pathways in pancreatic cancer was established, with napabucasin, a JAK/STAT inhibitor, showing promising results in an early-stage clinical trial [57,58]. The involvement of PI3K/Akt and MAPK signaling in the PANC-1 cell line has been previously reported for methyl 2-cyano-3,12-dioxoolean-1,9-dien-28-oate, a semisynthetic pentacyclic triterpene [59].

Collectively, as similar natural and semisynthetic triterpenes have modulated pathways encompassing our target proteins in melanoma, breast and pancreatic cancer, these observations suggest that AP5 may exert its anticancer activity through similar pathways, although experimental validation is needed.

#### 2.3.4. Protein–Protein Interaction Network of Hub Targets and Pathway Cross-Validation (STRING + Enrichr)

**Figure 13:**
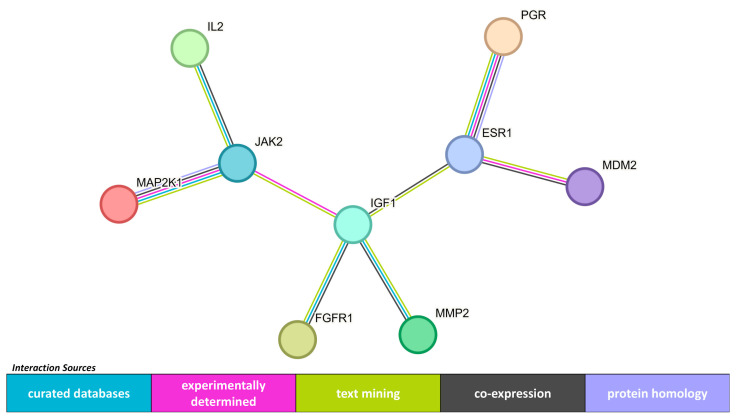
Protein–protein interaction network for the nine hub targets (STRING, confidence threshold > 0.700). Edge colors denote the different interaction sources, as indicated in the legend at the bottom of the figure.

A protein–protein interaction (PPI) network of the nine hub targets was built with a high confidence interaction threshold (>0.700) in STRING (Figure 13, Supplementary File: Table S4) to understand how the hub targets interact in a biological system. The network showed IGF1 as a central node interacting with JAK2M, ESR1, FGFR1, and MMP2. Additionally, ESR1 acted as a secondary node, linking IGF1 with PGR and MDM2, while JAK2 linked IGF1 with MAP2K1 and IL2. The interactions, depicted as edges, are supported by multiple sources of evidence, including curated databases and experimentally confirmed interactions, reinforcing their biological relationship [60].

**Figure 14:**
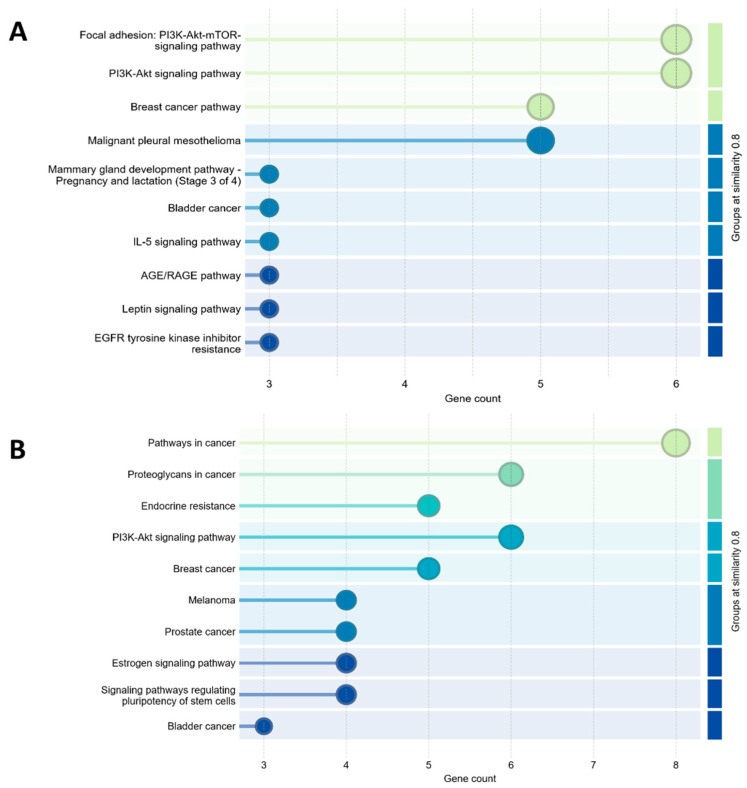
Pathway enrichment analysis for the nine hub targets represented as bubble plots (STRING); Panel (**A**): WikiPathways and Panel (**B**): KEGG Pathways. The bubble size represents the gene count identified in each pathway, and the color gradient, blue to light green, indicates the statistical significance based on the false discovery rate value.

**Figure 15:**
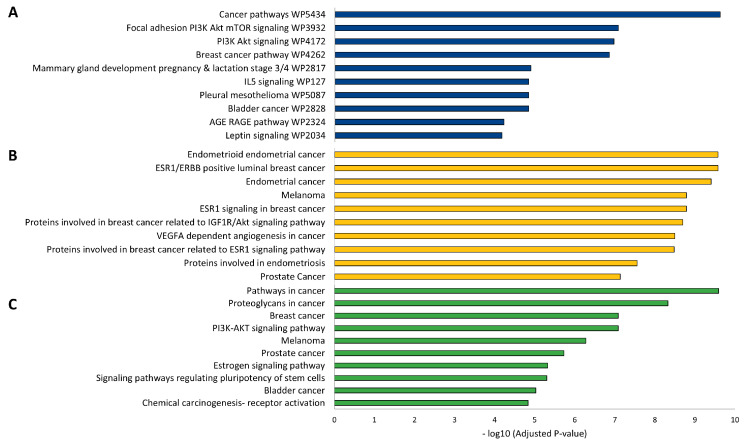
Pathway enrichment analysis for the nine hub targets (Enrichr); Panel (**A**): WikiPathways 2024, Panel (**B**): Elsevier Pathway Collection and Panel (**C**): KEGG 2026. The length of the bars corresponds to the significance score (−log10 *p*-value) of the targets in the pathway.

To identify the core pathways and cross-validate the global enrichment analysis (Metascape), a second enrichment analysis focused on the nine hub targets identified was performed using the STRING built-in enrichment tool and Enrichr platform across multiple databases (Figure 14 and Figure 15, Supplementary File, Tables S5 and S6). Both platforms highlighted the significance of the hub targets in breast cancer and PI3K-Akt signaling pathways across multiple databases. Additionally, the enrichment analysis of the Elsevier Pathway Collection (Figure 15B) highlighted the ESR1/ERBB positive luminal breast cancer and ESR1 signaling in breast cancer. Moreover, the melanoma pathway was also among the top 10 enriched pathways on both platforms. Collectively, the cross-platform validation of PI3K-Akt signaling, breast cancer, and melanoma-related pathways across both enrichment approaches supports the potential involvement of these pathways in the anticancer activity of Bet and AP1–5, particularly AP5, although biological validation is required.

While the network pharmacology is a promising field in drug design, it has inherent limitations such as reliance on incomplete biological databases, oversimplification of biological systems and the inability to account for molecular interactions [61]. Therefore, to assess the binding affinity for AP5–target interactions suggested by the network analysis, we employed molecular docking.

### 2.4. Molecular Docking and Molecular Dynamics Simulation

Molecular docking is a computational method used to predict the binding affinity between a molecule and protein targets, widely employed in natural compound-based drug discovery to identify and validate potential compound–target interactions [62]. Molecular docking was used to assess the binding affinity of AP5 against the top targets identified by CytoHubba degree ranking that were also predicted to interact with AP5 by PharmaMapper (ESR1, IGF1, MAP2K1, IL2, MDM2, MMP and PGR). Docking results are presented in Table 3. MMP-2 showed the most favorable docking score for AP5 (compared to the native ligand) among all validated targets, suggesting the strongest predicted binding affinity, whereas IGF1 was not included in the final docking comparison because the corresponding docking protocol did not meet the predefined validation step.

**Figure 16:**
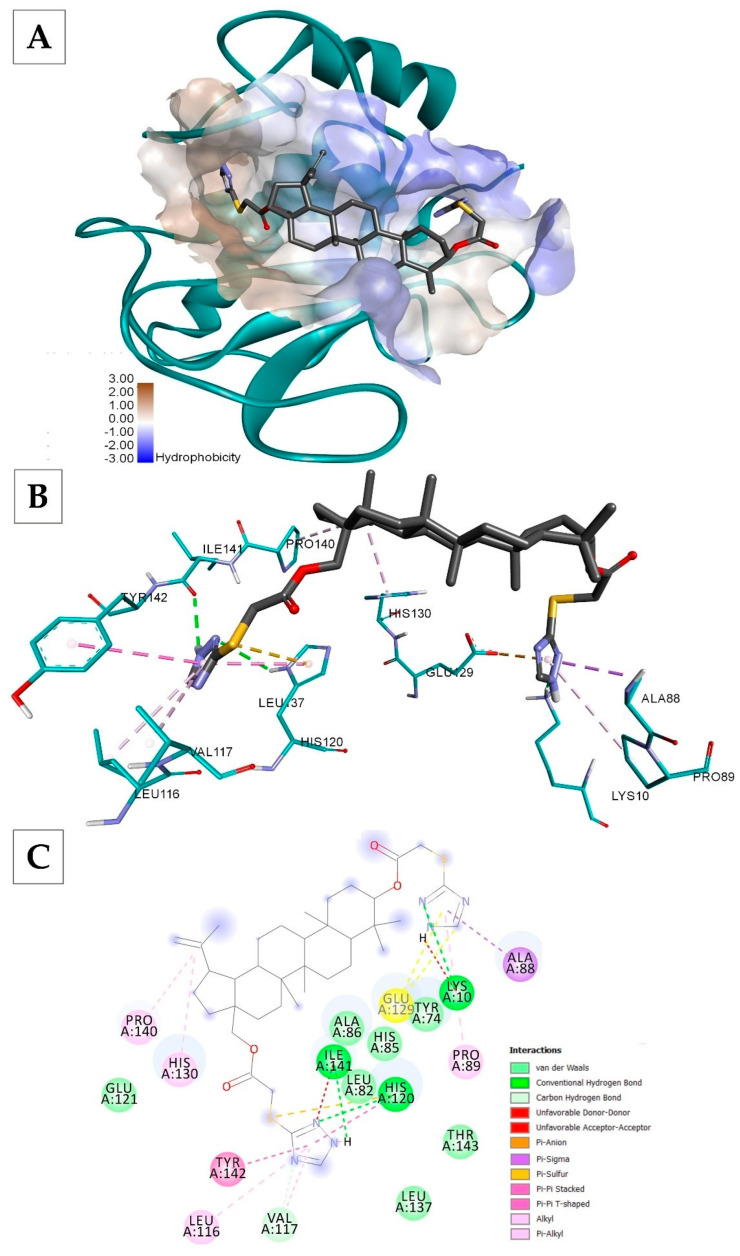
Predicted binding mode of AP5 within the MMP-2 binding pocket. (**A**) Three-dimensional representation of AP5 within the MMP-2 active site, shown together with a hydrophobic surface rendering of the binding pocket. (**B**) Detailed 3D interaction profile of AP5 with the interacting amino acid residues. (**C**) Two-dimensional interaction diagram highlighting the main ligand–target interactions.

Binding mode analysis suggests that AP5 is well accommodated inside the MMP-2 binding pocket, occupying an extended region of the active site (Figure 16A). The interaction profile shows that the docked compound is stabilized mainly by hydrophobic and π-related interactions, together with several hydrogen bonds (HBs). The betulin triterpenic core interacts predominantly through van der Waals and alkyl/π-alkyl interactions, involving only two residues, namely His130 and Pro140 (Figure 16B,C). On the other hand, both triazole-containing side chains contribute by forming the majority of polar and aromatic interactions. Several contacts are observed with residues such as Lys10, Ala84, Ala88, Pro89, Leu 116, Val 117, His120, Glu129, Tyr74, Leu82, Ile141 and, Tyr142, including conventional hydrogen bonds, carbon hydrogen bonds, π-anion/π-sulfur interactions and π-alkyl contacts.

The AP5–MMP-2 complex was submitted to post-docking Prime MM-GBSA analysis. The obtained results further supported the favorable interaction of AP5 with the target protein (Table 4). The complex exhibited a ΔGbind value of −43.81 kcal/mol, which indicates favorable binding in the minimized docking complex. Protein–ligand interaction was mainly supported by van der Waals and Coulombic contributions, along with lipophilic, packing and hydrogen-bond interactions. The positive Solv_GB value was consistent with the expected solvation penalty upon complex formation, and the ligand strain energy revealed a moderate energetic penalty related to ligand-bound conformation achievement. In summary, docking prediction was validated by MM-GBSA calculations and provided a rationale for selecting the AP5–MMP-2 complex for the following molecular dynamics simulation.

Following molecular docking, the AP5–MMP-2 complex, which showed the most favorable predicted binding affinity, was selected for molecular dynamics simulation (MDS).

**Figure 17:**
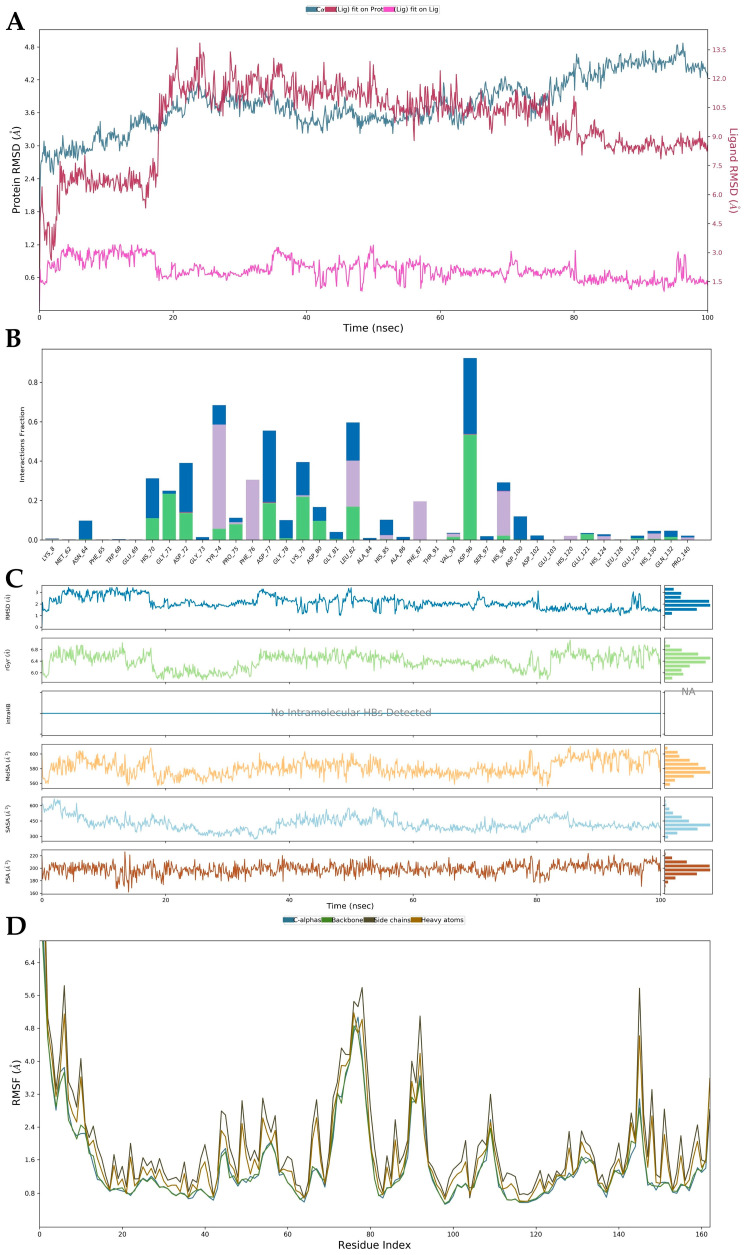
Molecular dynamics simulation analysis of the AP5–MMP-2 complex over 100 ns. (**A**) Protein backbone RMSD and ligand RMSD profiles during the simulation. (**B**) Protein–ligand contact histogram showing the interaction fraction of AP5 with MMP-2 residues throughout the trajectory. (**C**) Ligand property analysis, including ligand RMSD, radius of gyration, intramolecular hydrogen bonds, molecular surface area, solvent-accessible surface area, and polar surface area. (**D**) Protein RMSF profile showing residue-level flexibility for Cα atoms, backbone atoms, side chains, and heavy atoms.

During the 100 ns MDS run, the AP5–MMP-2 complex showed an initial adaptation phase followed by a relatively stable trajectory. The protein RMSD increased during the first part of the simulation and remained mostly within a moderate range, with a slight increase toward the end (Figure 17A). Despite the slight RMSD increase at the end, there was no clear evidence of ligand dissociation. The ligand-fit-on-ligand RMSD remained comparatively low, suggesting that the ligand’s internal conformation was mostly preserved. Therefore, the observed RMSD variation can be cautiously interpreted as conformational flexibility and receptor adaptation around the ligand rather than definitive complex destabilization. Nevertheless, longer or independent replicate simulations would be required to confirm long-term convergence. The ligand RMSD calculated as ligand fit on protein showed higher values, indicating possible positional rearrangement of AP5 within the binding pocket. However, the ligand fit on ligand RMSD remained comparatively low, supporting that the ligand’s internal conformation was mostly preserved (Figure 17A).

The protein–ligand contact analysis showed persistent interactions with several residues, namely Asp96, Tyr74, Asp77, Leu82, His98, His70, Lys79, Asp80 and Glu121, suggesting that AP5 remained bound with the MMP-2 catalytic region through multiple interaction types (Figure 17B,C). The RMSF profile of the target protein further supported this interpretation, showing that most residues displayed low fluctuations, while higher flexibility was restricted to specific regions, especially the N-terminal segment and loop regions around residues 70–80, 90–95 and 140–150 (Figure 17D). Therefore, the observed RMSD changes are likely related to localized flexibility and receptor adaptation around the ligand rather than global protein destabilization. Overall, the MDS results support the stability of the AP5–MMP-2 complex and reinforce the favorable docking prediction for this target.

The preferential docking-based targeting of MMP-2 by AP5 is biologically plausible given that several pentacyclic triterpenes have previously been reported to inhibit invasion and metastasis-related gelatinases. The antimetastatic effect of betulin, the parent scaffold of AP5, in non-small-cell lung cancer cells has been described to be mediated by the inhibition of MMP-2/MMP-9 [63]. Similarly, betulinic acid inhibited migration and invasion in breast and colorectal cancer models, correlated with modulation of MMP expression and an increase in TIMP-2 levels [64]. Other pentacyclic triterpenes, such as ursolic and oleanolic acids, were also found to inhibit the migration and invasion of colon cancer cells via decreased secretion/activity of MMP-2 and MMP-9 [65]. Thus, the predicted interaction of AP5 with MMP-2 is in line with previous reports linking pentacyclic triterpenes with antimetastatic mechanisms involving gelatinase inhibition.

### 2.5. In Silico ADMET Predictions

**Figure 18:**
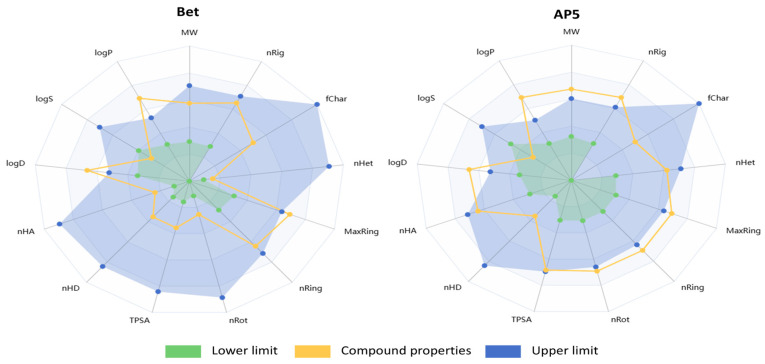
Radar charts illustrating the physicochemical properties of Bet and AP5. MW: molecular weight; logP: partition coefficient; logS: aqueous solubility; logD: distribution coefficient; TPSA: topological polar surface area; nHA: number of hydrogen bond acceptors; nHD: number of hydrogen bond donors; nRot: number of rotatable bonds; nRing: number of rings; MaxRing: number of atoms in the largest ring; nHet: number of heteroatoms; fChar: formal charge; nRig: number of rigid bonds.

In silico assessments of physicochemical and ADMET parameters are valuable tools in early-stage drug development that can highlight potential pharmacokinetic and toxicological liabilities of drug candidates, guiding future in vitro and in vivo research [66,67]. For this assessment, AP5 was selected based on the superior cytotoxicity and network pharmacology results, with Bet, the parent compound, included for comparison based on the availability of previous studies describing several physicochemical and ADMET properties in preclinical models [68,69,70]. The physicochemical and ADMET profile of Bet and AP5 was predicted using ADMETlab 3.0 [66], and the key results are summarized in Figure 18 and Table 5. The full numerical values are included in the Supplementary File (Table S7). As depicted in Figure 18, Bet displays physicochemical properties largely contained in the optimal range, while the properties of AP5 expand to borderline or beyond the optimal range for several parameters (MW, nRig, MaxRing, nRing, nRot, log D and logP). However, these characteristics are common for natural origin derivatives that frequently fail to comply with Lipinski’s rule of five for drug-likeness [71].

The ADMET prediction profile indicates that AP5 has an acceptable Caco-2 permeability and high predicted intestinal absorption. Moreover, AP5 does not act as a P-gp substrate, an aspect particularly relevant in breast cancer models, where P-gp overexpression is associated with drug resistance [72,73], suggesting AP5 might evade the P-gp-mediated resistance. Additionally, the low predicted blood–brain barrier (BBB) penetration suggests that AP5 cannot reach therapeutic concentrations in the central nervous system. Still, the mechanism is more likely independent of the P-gp, a major constituent of the BBB [74]. However, the low penetration of the BBB is considered favorable in avoiding neurological toxicity, the fourth cause of medicinal product withdrawal worldwide [75], particularly relevant as AP5 was targeted at non-central nervous system malignancies.

The increased stability of AP5 in the presence of human liver microsomes (HLMs) suggests reduced hepatic metabolism, which is consistent with lower plasma clearance (Cl: 0.85 mL/min/kg) and increased systemic half-life (T_1/2_: 1.43 h) compared to Bet (Cl: 9.9 mL/min/kg, T_1/2_: 0.72 h). These findings are consistent with previous studies demonstrating that compounds with low stability are rapidly excreted. However, high microsomal stability, as predicted for AP5, does not always correlate with low clearance in vivo, due to the contribution of complementary metabolic and excretory pathways [76], suggesting these findings should be interpreted with caution and be further validated.

The toxicity predictions indicate Bet and AP5 have a moderate to high risk of drug-induced liver injury (DILI), genotoxicity, carcinogenicity, nephrotoxicity, and skin sensitization. However, until further validation, these should be interpreted with caution as computational models can overestimate the toxicity risks associated with complex structures such as natural-based compounds [77]. Contrary to the predicted toxicity, numerous studies have highlighted the anticancer effect of Bet in preclinical models [78], as well as protective effects in rodent models of hepatic and kidney injuries [79,80,81]. Additionally, an oleogel containing Bet showed good compatibility in the HaCaT cell line in vitro, reduced irritative potential in the HET-CAM assay and decreased erythema in a murine skin model, indicating favorable skin tolerance [82]. Therefore, as the lack of experimental validation for Bet predictions shows, the toxicity predicted for AP5 might also be overestimated in silico. Our preliminary assessment of AP5 on the HaCaT cell line showed that only the highest concentration tested (100 μM) induced slight cytotoxicity, indicating reduced toxicity in this context. Nonetheless, the toxicity risks predicted for AP5 would need to be experimentally validated to address the safety concerns.

## 3. Materials and Methods

### 3.1. Chemistry

#### 3.1.1. Reagents and Instruments

The reagents and the 60 F254 silica gel–aluminium coated plates used for the chemical synthesis and thin-layer chromatography were acquired from Merck (Darmstadt, Germany). The reagents were used without additional purification. The melting points were recorded using a Biobase melting point analyzer (Biobase Group, Jinan, China). The ^1^H and ^13^C NMR experiments were performed using a Bruker Avance NEO 400 MHz Spectrometer (Bruker, Karlsruhe, Germany) that was equipped with a 5 mm QNP direct detection probe and z-gradients. The spectra were recorded under standard conditions in either DMSO-d_6_ or CDCl_3_ and were referenced on the residual peak of the solvent (^1^H: 2.51 ppm for DMSO-d_6_ or 7.26 ppm for CDCl_3_; ^13^C: 39.5 ppm for DMSO-d_6_ or 77.0 for CDCl_3_), For the AP1, AP3 and AP5 derivatives, we first recorded the NMR spectra in DMSO-d_6_; then, in each NMR tube containing the solutions, a drop of trifluoroacetic acid (TFA) was added; the solutions were vortexed for 5 min at 500 rpm, and then the NMR experiments were recorded again. All the NMR experiments were recorded using standard parameter sets, as provided by Bruker. Fourier-transform infrared spectroscopy (FTIR) analysis was conducted using KBr pellets on a Shimadzu IR Affinity-1S spectrometer (400–4000 cm^−1^ range and a 4 cm^−1^ resolution).

#### 3.1.2. Synthesis of Bet-(ClAc)2

Bet-(ClAc)2 was obtained through a slightly modified Steglich-type esterification method [19] that involved the reaction between Bet (2.9 mmoles, 1.28 g) and chloroacetic acid (29 mmoles, 2.74 g) in 60 mL dichloromethane (DCM), in the presence of dicyclohexylcarbodiimide (DCC, 29 mmoles, 5.98 g), used as coupling agent, and 4-dimethylaminopyridine (DMAP, 4.64 mmoles, 0.566 g), used as catalytic agent, at room temperature in an inert atmosphere for 24 h. The reaction mixture was initially filtered to eliminate the solid byproduct formed (dicyclohexylurea), and then Bet-(ClAc)2 was separated from the filtrate by column chromatography using a mixture of chloroform and ethyl acetate in a volume ratio of 100:1.

Betulin-3,28-O-di(chloroacetate) (Bet-(ClAc)2), white powder, 1.3 g, 2.185 mmoles obtained, yield 75%, m.p. 87–94 °C, ^1^H NMR (CDCl_3_, 400.13 MHz, δ, ppm): 4.69 (s, 1H, H29a), 4.59 (s, 1H, H29b), 4.56 (dd, J = 6.0 Hz, J = 10.7 Hz, 1H, H3), 4.40 (d, J = 10.8 Hz, 1H, H28a), 4.08 (s, 2H, H34), 4.05 (d, J = 2.5 Hz, 2H, H32), 3.95 (d, J = 11.0 Hz, H28b), 2.46–2.41 (m, 1H, H19), 1.99–0.78 (betulinic protons). ^13^C NMR (CDCl_3_, 100.6 MHz, δ, ppm): 167.8 (C33), 167.1 (C31), 149.9 (C20), 110.0 (C29), 83.3 (C3), 64.8 (C28), 55.3 (C5), 50.2 (C9), 48.8 (C18), 47.7 (C19), 46.5 (C17), 43.4 (C14), 42.7 (C8), 41.2–41.0 (C32,34), 38.3 (C1), 38.0 (C4), 37.6 (C13), 37.1 (C10), 34.4 (C7), 34.1 (C16), 29.6 (C21), 29.5 (C15), 27.9 (C23), 27.0 (C12), 25.1 (C2), 23.5 (C22), 20.8 (C11), 19.1 (C30), 18.1 (C6), 16.4 (C24), 16.1 (C27), 16.0 (C25), 14.7 (C26). FTIR [KBr] (cm^−1^) relevant peaks: 2943, 2877 (aliphatic C-H stretching); 1757, 1726 (ester C=O stretching) 1176, 1045, 1006 (C-O/C-O-C ester stretching);

#### 3.1.3. General Procedure for the Synthesis of AP1–5

For the synthesis of AP1–5 derivatives, Bet-(ClAc)2 (0.5 mmoles, 297 mg) and anhydrous K_2_CO_3_ (5 mmoles, 691 mg) were added in DMF (10 mL) and stirred for 10 min at room temperature. After the addition of the 5-substituted-1,2,4-triazole-3-thiols (1.25 mmoles), the reaction mixture was stirred for an additional 48 h at room temperature. After completion, the reaction mixture was diluted with water and extracted with ethyl acetate. The organic mixture was subsequently washed with water and brine, dried over anhydrous Na_2_SO_4_, and cromatographed over silica using various ratios of hexane:ethyl acetate.

Betulin-3,28-O-diyl-bis[5-(4-chlorophenyl)-1H-1,2,4-triazol-3-yl)sulfanylacetate]) (AP1), white powder, 161 mg, 0.17 mmoles obtained, yield 34%, m.p. 147–154 °C, ^1^H NMR (DMSO-d_6_ + TFA, 400.13 MHz, δ, ppm): 7.94–7.91 (m, 4H, H38,42,46,50), 7.53–7.50 (m, 4H, H39,41,47,49) 4.63 (s, 1H, H29a), 4.49 (s, 1H, H29b), 4.37–4.32 (m, 1H, H3), 4.15 (d, J = 10.9 Hz, 1H, H28a), 4.08–4.03 (m, 4H, H32,34), 3.71 (d, J = 10.9 Hz, H28b), 2.38–2.33 (m, 1H, H19), 1.84–0.63 (betulinic protons). 13C NMR (DMSO-d6 + TFA, 400.13 MHz, δ, ppm): 169.2 (C33), 168.7 (C31), 157.1 (C36,44), 156.6 (C35,43), 150.2 (C20), 134.9 (C40,48), 129.5 (C39,41,47,49), 128.1–128.0 (C38,42,46,50), 127.7 (C37,45), 110.3 (C29), 81.9 (C3), 63.6 (C28), 54.9 (C5), 49.8 (C9), 48.5 (C18), 47.5 (C19), 46.3 (C17), 42.5 (C14), 40.6 (C8), 38.0 (C1), 37.9 (C4), 37.4 (C13), 36.9 (C10), 34.4 (C32,34), 34.2 (C30), 33.9 (C7), 33.5 (C16), 29.3 (C21,15), 27.8 (C23), 26.5 (C12), 24.6 (C2), 23.2 (C22), 19.6 (C11), 18.1 (C6), 16.6 (C24), 15.8 (C27), 15.6 (C25), 14.8 (C26). FTIR [KBr] (cm^−1^) relevant peaks: 3236 (N-H/heteroaromatic stretching); 2945, 2872 (aliphatic C-H stretching); 1728 (ester C=O stretching) 1275, 1146, 1092, 1015 (C-O/C-O-C ester stretching); 1641, 1607 (C=C/C=N stretching from triazole and aromatic systems).

Betulin-3,28-O-diyl-bis[5-(4-methoxyphenyl)-1H-1,2,4-triazol-3-yl)sulfanylacetate]) (AP2), white powder, 164 mg, 0.175 mmoles obtained, yield 35%, m.p. 152–158 °C, ^1^H NMR (CDCl_3_, 400.13 MHz, δ, ppm): 7.89–7.86 (m, 4H, H38,42,47,51), 6.91 (d, J = 8.4 HZ, 4H, H39,41,48,50), 4.65 (s, 1H, H29a), 4.56 (s, 2H, H29b, H3), 4.50 (dd, J = 5.6 Hz, J = 9.8 Hz, 1H, H3), 4.32 (d, J = 10.9 Hz, 1H, H28a), 3.98–3.87 (m, 5H, H28b, H32,34), 3.82–3.81 (m, 6H, H43,52), 2.37–2.35 (m, 1H, H19), 1.93–0.37 (betulinic protons). ^13^C NMR (CDCl_3_, 100.6 MHz, δ, ppm): 170.1 (C33), 169.6 (C31), 161.2–161.1 (C40,49), 158.6–158.3 (C36,46), 156.8–156.5 (C35,46), 149.9 (C20), 128.0 (C38,42,47,51), 120.5–120.3 (C37,C46), 114.2–113.8 (C39,41, 48,50), 109.9 (C29), 83.1 (C3), 65.8 (C28), 55.3 (C43,52,C5), 50.1 (C9), 48.7 (C18), 47.7 (C19), 46.4 (C17), 42.6 (C14), 40.7 (C8), 38.2 (C32,34), 37.8 (C1), 37.5 (C4), 36.9 (C13), 34.8 (C10), 34.5 (C7), 34.4 (C16), 29.5 (C21), 28.4 (C15), 27.8 (C23), 26.9 (C12), 25.1 (C2), 23.6 (C22), 20.7 (C11), 19.1 (C30), 18.0 (C6), 16.4 (C24), 15.8 (C27), 15.2 (C25), 14.7 (C26). FTIR [KBr] (cm^−1^) relevant peaks: 3198 (N-H/heteroaromatic stretching); 2943, 2872 (aliphatic C-H stretching); 1730 (ester C=O stretching) 1254, 1177, 1146, 1030, 1007 (C-O/C-O-C ester stretching); 1641, 1614, 1582 (C=C/C=N stretching from triazole and aromatic systems).

Betulin-3,28-O-diyl-bis[5-(phenyl)-1H-1,2,4-triazol-3-yl)sulfanylacetate]) (AP3), white powder, 166 mg, 0.19 mmoles obtained, yield 38%, m.p. 141–147 °C, ^1^H NMR (DMSO-d_6_ + TFA, 400.13 MHz, δ, ppm): 7.93–7.91 (m, 4H, H38,42,46,50), 7.51–7.45 (m, 6H, H39–41,47–49) 4.65 (s, 1H, H29a), 4.52 (s, 1H, H29b), 4.37 (dd, J = 5.5 Hz, J = 10.8 Hz, 1H, H3), 4.21 (d, J = 10.9 Hz, 1H, H28a), 4.10–4.04 (m, 4H, H32,34), 3.76 (d, J = 10.9 Hz, H28b), 2.43–2.36 (m, 1H, H19), 1.89–0.70 (betulinic protons). ^13^C NMR (DMSO-d_6_ + TFA, 400.13 MHz, δ, ppm): 168.90 (C33), 168.3 (C31), 156.9 (C35,36,43,44), 149.7 (C20), 145.9 (C36,38), 130.0–129.9 (C40,49), 129.0–128.9 (C39,41,47,49), 127.8 (CC37,45), 125.9 (C38,42,46,50), 109.9 (C29), 81.3 (C3), 63.0 (C28), 54.5 (C5), 49.4 (C9), 48.1 (C18), 47.0 (C19), 46.1 (C17), 42.2 (C14), 40.2 (C8), 37.6 (C1), 37.4 (C4), 37.0 (C13), 36.5 (C10), 33.9 (C32,34), 33.7 (C30), 33.5 (C7), 33.2 (C16), 28.9 (C21,15), 27.4 (C23), 26.5 (C12), 24.6 (C2), 23.2 (C22), 20.1 (C11), 18.7 (C6), 16.2 (C24), 15.7 (C27), 15.4 (C25), 14.4 (C26). FTIR [KBr] (cm^−1^) relevant peaks: 3227 (N-H/heteroaromatic stretching); 2943, 2872 (aliphatic C-H stretching); 1728 (ester C=O stretching) 1146, 1072, 1005 (C-O/C-O-C ester stretching); 1641, 1553 (C=C/C=N stretching from triazole and aromatic systems).

Betulin-3,28–O-diyl-bis[5-(4-dimethylaminophenyl)-1H-1,2,4-triazol-3-yl)sulfanylacetate]) (AP4), white powder, 150 mg, 0.156 mmoles obtained, yield 31%, m.p. 143–150 °C, ^1^H NMR (CDCl_3_, 400.13 MHz, δ, ppm): 8.85 (d. J = 8.9 Hz, 4H, H38,42,48,52), 6.73–6.71 (m, 4H, H39,41,49,51), 4.84–4.80 (m, 4H, H32,34), 4.70 (s, 1H, H29a), 4.60–4.57 (m, 2H, H29b, H3), 4.40 (d, J = 10.8 Hz, 1H, H28a), 3.97 (d, J = 10.9 Hz, 1H, H28a), 3.07 (s, 12H, H43,44,53,54), 2.46–2.41 (m, 1H, H19), 2.01–0.79 (betulinic protons). ^13^C NMR (CDCl_3_, 100.6 MHz, δ, ppm): 168.8 (C33), 168.1 (C31), 166.1 (C35,36,45,46), 153.3–153.2 (C40,50), 150.0 (C20), 131.7 (C38,42,48,52), 116.7–116.5 (C37,C47), 111.2–111.1 (C39,41, 49,51), 109.9 (C29), 82.2 (C3), 63.6 (C28), 61.0–60.8 (C32,34), 55.3 (C5), 50.2 (C9), 48.8 (C18), 47.7 (C19), 46.5 (C17), 42.7 (C14), 40.9 (C8), 40.3 (C43,44,53,54), 38.3 (C1), 37.8 (C4), 37.6 (C13), 37.0 (C10), 34.4 (C7), 34.0 (C16), 29.6 (C21), 29.5 (C15), 27.9 (C23), 27.0 (C12), 25.1 (C2), 23.6 (C22), 20.8 (C11), 19.1 (C30), 18.1 (C6), 16.4 (C24), 16.1 (C27), 16.0 (C25), 14.7 (C26). FTIR [KBr] (cm^−1^) relevant peaks: 3196 (N-H/heteroaromatic stretching); 2941, 2872 (aliphatic C-H stretching); 1730 (ester C=O stretching) 1253, 1176, 1145, 1029 (C-O/C-O-C ester stretching); 1643, 1614, 1581 (C=C/C=N stretching from triazole and aromatic systems).

Betulin-3,28-O-diyl-bis[5-(1H-1,2,4-triazol-3-yl)sulfanylacetate] (AP5), white powder, 108 mg, 0.15 mmoles obtained, yield 30%, m.p. 105–112 °C, ^1^H NMR (DMSO-d_6_ + TFA, 400.13 MHz, δ, ppm): 8.45–8.44 (m, 2H, H36,38), 4.68 (s, 1H, H29a), 4.54 (s, 1H, H29b), 4.36 (dd, J = 5.2 Hz, J = 11.1 Hz, 1H, H3), 4.26 (d, J = 10.9 Hz, 1H, H28a), 4.04–3.98 (m, 4H, H32,34), 3.77 (d, J = 11.0 Hz, H28b), 2.46–2.40 (m, 1H, H19), 1.88–0.72 (betulinic protons). ^13^C NMR (DMSO-d_6_ + TFA, 400.13 MHz, δ, ppm): 169.0 (C33), 168.4 (C31), 156.5 (C35,37), 149.8 (C20), 145.9 (C36,38), 109.9 (C29), 81.3 (C3), 62.8 (C28), 54.6 (C5), 49.5 (C9), 48.2 (C18), 47.0 (C19), 46.1 (C17), 42.2 (C14), 40.4 (C8), 37.6 (C1), 37.5 (C4), 37.0 (C13), 36.5 (C10), 33.9 (C32,34), 33.6 (C30), 33.5 (C7), 33.4 (C16), 30.9 (C21), 28.9 (C15), 27.4 (C23), 26.5 (C12), 24.7 (C2), 23.2 (C22), 20.3 (C11), 18.7 (C6), 16.2 (C24), 15.7 (C27), 15.6 (C25), 14.4 (C26). FTIR [KBr] (cm^−1^) relevant peaks: 3125 (N-H/heteroaromatic stretching); 2943, 2872 (aliphatic C-H stretching); 1726 (ester C=O stretching) 1279, 1159, 1078, 1005 (C-O/C-O-C ester stretching); 1641 (C=C/C=N stretching from triazole and aromatic systems).

### 3.2. Biological Assessment

#### 3.2.1. Cell Culture

The cells selected for this study were human keratinocytes—HaCaT (purchased from CLS Cell Lines Services GmbH, Eppelheim, Germany), human malignant melanoma cells—A375, human breast cancer cells—MCF-7 and human pancreatic cells—PANC-1 (acquired from American Type Culture Collection, ATTC, Lomianki, Poland). HaCaT, A375 and PANC-1 were propagated in Dulbecco’s Modified Eagle Medium (DMEM) high glucose, supplemented with 10% fetal bovine serum (FBS) and 1% mixture of penicillin/streptomycin (100 IU/mL). MCF-7 cells were cultured in Eagle’s Minimum Essential Medium (EMEM) supplemented with 10% FBS, 1% antibiotic mixture and 0.01 mg/mL human recombinant insulin. All cells were maintained in a humidified incubator at 37 °C with 5% CO_2_.

#### 3.2.2. Cell Viability Assessment

The cell viability of HaCaT, A375, MCF-7 and PANC-1 was measured by means of Alamar blue. Briefly, the cells (1 × 10^4^ cells/well) were seeded onto 96-well plates until they reached 80–85% confluence. After reaching the desired confluence, the old medium was removed using an aspiration station and replaced with fresh medium containing increasing concentrations (1, 5, 10, 50 and 100 μM) prepared from 10 mM stocks for each tested compound. After 48 h, the plates were incubated with 20 μL/well of Alamar Blue reagent for 3 h at 37 °C. The absorbance was measured using the xMark™ Microplate Spectrophotometer, Bio-Rad (Hercules, CA, USA), at two wavelengths, 570 nm and 600 nm. All experiments were performed in triplicate.

#### 3.2.3. Cell Morphology Assessment

HaCat, A375, MCF-7 and PANC-1 were seeded onto 24-well plates (10^5^ cells/well) until they reached 80–85% confluence. After reaching the desired confluence, the cells were treated for 48 h with the highest tested concentration for AP1-AP4 (100 μM), and with the corresponding IC_50_ values for AP5. Separately, some wells were treated with Staurosporine (5 μM) as a positive control for necrosis. Then the cells were fixed with methanol for 15 min, permeabilized with Triton X 0.01% in PBS for another 15 min and blocked with bovine serum albumin (BSA) 3% at room temperature for 30 min. Subsequently, the cells were stained with beta-actin monoclonal antibody (Thermo Fisher Scientific, Inc., Waltham, MA, USA) at a 1:2000 dilution in BSA 3% for 1 h at room temperature, and next they were treated with Alexa fluor Goat-anti-Mouse Secondary Antibody (Thermo Fisher Scientific, Inc., Waltham, MA, USA) at a 1:1000 dilution in BSA 3% for 30 min in the dark. Finally, the cells were colored with Hoechst 33258 solution for 5 min in the dark. The nuclear morphological changes were analyzed using the EVOS™ M5000 Imaging System equipped with a highly sensitive CMOS camera (Thermo Fisher Scientific, Inc., Waltham, MA, USA).

#### 3.2.4. Caspase-3/7 Assay

The apoptosis analysis was developed in accordance with the manufacturer’s recommendations [83] and was previously described [84]. Briefly, the stained cells treated previously with AP1, AP2, AP3, AP4 and AP5, utilizing their IC_50_ values/100 μM for 24 h, were analyzed live employing an automated fluorescent cell counter equipped with a GFP filter set (Countess™ 3 FL Automated Cell Counter, Thermo Fisher Scientific, Inc., Waltham, MA, USA). This detection method involves the cleavage of a four-amino acid peptide from the nucleic acid-binding dye that further allows the dye to bind to DNA in the nucleus and produce a fluorescent signal in apoptotic cells.

#### 3.2.5. DCFDA/H_2_DCDFA Assay

The production of ROS in all cells treated with AP1–5 was determined using the 2′,7′-dichlorodihydrofluorescein diacetate (DCFDA/H_2_DCFDA) kit (ab113851, Abcam, Cambridge, MA, USA). The DCFDA protocol was performed in accordance with the manufacturer’s recommendations [85]. Briefly, cells were seeded in 96-well plates until reaching 80–85% confluence and treated with AP1–5 (IC_50_ values/100 μM) for 24 h. Following the treatment period, the culture medium was removed, and cells were washed and then incubated with DCFDA (20 µM) working solution for 30–45 min at 37 °C in the dark to allow intracellular deacetylation of the probe. The DCFDA solution was then removed, and cells were washed with ROS buffer to eliminate excess dye. Fluorescence intensity was measured at 485/535 nm using the Synergy HTX Multi-Mode Reader microplate reader (Agilent Technologies, Santa Clara, CA, USA).

### 3.3. Statistical Analysis

One-way ANOVA followed by Dunnett’s post hoc test (GraphPad Prism version 6.0.0, GraphPad Software, San Diego, CA, USA) was used for the statistical analysis. The differences between the groups were considered statistically significant if *p* < 0.05, as follows: * *p* < 0.05, ** *p* < 0.01, and *** *p* < 0.001.

### 3.4. Network Pharmacology

#### 3.4.1. Ligand Preparation

The 2D structures of Bet and AP1–5 were initially sketched in BioviaDraw (v.2026, Dassault Systèmes), and then imported into Avogadro (v. 1.103.0) [86]. After the addition of hydrogen atoms, geometric optimization was performed using the Auto-Optimization Tool, employing the Universal Force Field and a Steepest Descent algorithm to ensure a relevant 3D conformation for subsequent target prediction.

#### 3.4.2. Reverse Pharmacophore Mapping (PharmaMapper)

The 3D structures of Bet and AP1–5 were subjected to reverse pharmacophore mapping for human protein targets using the PharmMapper server [42]. Then, to identify the targets with the highest complementarity, two criteria were employed: fit score > 3.0 and z’-score > 1.0. Moreover, to ensure data consistency across multiple platforms, we retrieved the UniProt IDs from the UniProt database [87]. The list of targets meeting the selection criteria is provided in the Supplementary Material.

#### 3.4.3. Gene Selection

Relevant genes in humans (*Homo sapiens*) for melanoma, breast and pancreatic cancer were identified using the GeneCards database [88] by searching the keywords “melanoma”, “breast cancer” and “pancreatic cancer”. For each type of cancer, the top 300 targets according to relevance score were analyzed and further refined to exclude RNA fragments, functional elements, genetic loci, and pseudogenes. The refined gene lists for each type of cancer were first translated into their corresponding encoded proteins using UniProt identifiers and then cross-referenced with the targets identified for Bet and AP1–5 using the Interactivenn tool [89]. To construct the Venn diagrams, we initially eliminated targets identified for multiple derivatives to ensure that each target is counted once, regardless of its connection with multiple compounds.

#### 3.4.4. Global Pathway Enrichment (Metascape)

The potential targets of Bet and AP1–5 in melanoma, breast and pancreatic cancer were subjected to functional enrichment analysis using the Metascape (v3.5) platform [43]. An express analysis was conducted to identify significant enriched pathways across Gene Ontology Biological Processes, Reactome Gene sets, KEGG and WikiPathways databases.

#### 3.4.5. Network Construction (Cytoscape) and Hub Target Identification (CytoHubba)

Cytoscape (v3.10.4) [90] was used to construct the compound–target network, employing a discrete mapping strategy to differentiate the nodes; hexagons were assigned to the compounds (Bet and AP1–5), and ellipses were used to represent protein targets. Additionally, a dual continuous mapping strategy was applied to the network edges to visualize both chemical affinity and biological relevance; width was scaled proportionally to the PharmMapper Fit Score, and a color gradient from yellow to violet was applied proportionally to the GeneCards relevance score for breast cancer, the most sensitive cancer type from our preliminary cytotoxicity assessment. A radial layout was subsequently applied to organize the network. Finally, to identify the hub targets, the cytoHubba plugin was used to rank the nodes based on their topological network significance. The top 15 nodes were extracted, comprising the 6 compounds (Bet, AP1–5) and 9 hub protein targets.

#### 3.4.6. Protein–Protein Interaction and Enrichment Cross-Validation (STRING, Enrichr)

To validate the functional and biological interactions among the 9 hub protein targets, a dual validation approach was used. Initially, PPI was employed using the STRING database [91], analysis restricted to *Homo sapiens* and applying a high confidence threshold (>0.700) to ensure the PPI network is robust. Within STRING, the top 10 significantly enriched pathways from WikiPathways and KEGG were extracted to cross-reference the functional clusters. Subsequently, an enrichment analysis was performed in the Enrichr platform [92,93,94] across WikiPathways 2024, Elsevier Pathway Collection and KEGG 2026 databases, selecting the top 10 pathways ranked by their adjusted *p*-value for further analysis.

### 3.5. Molecular Docking and Molecular Dynamics Simulation

Molecular docking was performed with PyRx (version 0.8) [95], using AutoDock Vina’s scoring function (v1.1.2). Protein target files corresponding to ESR1, IGF1, IL2, KDR, MAP2K1, MDM2, MMP2, and PGR were selected according to the PharmaMapper server and subsequently downloaded from the RCSB Protein Data Bank [96]. Docking protocol validation was achieved by redocking the native ligand into each target’s original binding site and comparing the resulting pose with the experimental conformation. The validation threshold between docked and experimental RMSD of each native ligand pose was set to 2 Å. The mol structure file of AP5 was converted to a 3D conformation by the Open Babel Module within PyRx, using the Uff force field. Subsequently, the structure was converted to PDBQT format. The docking parameters, as well as the protein files submitted to the task, are presented in Table 6. For the case of PD entry 1HOV, which is an MRN-resolved structure, model 1 was used, since the literature suggested that no relevant differences in docking results were recorded when all 11 models were used [97]. Docking-related images were created using Discovery Studio Visualizer v20.1.0.19295 (Dassault Systèmes, Paris, France).

Molecular dynamics simulation of the AP5–1HOV protein–ligand complex was performed using the Desmond package implemented in Schrödinger (Schrödinger Release 2022-4, Schrödinger, LLC, New York, NY, USA). The selected docked complex was prepared using the System Builder module and solvated in an orthorhombic TIP3P water box with a 9 Å buffer distance. The system was neutralized by adding Na+ and Cl− ions. Energy minimization and equilibration were carried out using the default Desmond relaxation protocol. The production MD simulation was performed for 100 ns under NPT ensemble conditions at 300 K and 1 atm without restraints. Long-range electrostatic interactions and trajectory analyses were performed using Maestro Simulation Interaction Diagram tools. The stability of the complex was evaluated through RMSD and RMSF analyses of the protein backbone and ligand throughout the simulation time.

Binding free energies (ΔG_bind) were estimated using Prime MM-GBSA (Schrödinger Release 2022-4, Schrödinger, LLC, New York, NY, USA) for the AP5–1HOV protein–ligand complex. The docked protein–ligand complex obtained using Glide SP with post-docking minimization was used for the MM-GBSA calculations. Prime MM-GBSA calculations were performed using the OPLS4 force field and the VSGB implicit solvent model. Local minimization was applied to the ligand and protein residues within 5 Å of the ligand, while the remaining protein structure was kept fixed. The total binding free energy (ΔG_bind) together with the main energetic contributions (vdW, Coulomb, H-bond, Lipo, Packing, and Solv_GB) and ligand efficiency (LE) were evaluated.

### 3.6. In Silico ADMET Predictions

The ADMETlab 3.0 online platform was used to predict the physicochemical and ADMET parameters for Bet and AP5, imported through their SMILES strings [66].

## 4. Conclusions

The study presents the synthesis and evaluation of a novel series of C3/C28-bis-1,2,4-triazolyl-sulfanylacetate-Bet derivatives, AP1–5, as potential anticancer agents. Among the synthesized derivatives, AP5, containing two unsubstituted 1,2,4-triazoles, emerged as the lead compound, which showed selective cytotoxicity against three cell lines, namely MCF-7 (IC_50_: 7.41 μM), A375 (IC_50_: 20.36 μM), and PANC-1 (IC_50_: 20.92 μM), while the phenyl-substituted derivatives, AP1–4, displayed reduced potency, indicating that the triazole substituents influence biological activity. Among the tested cell lines, AP5 showed superior cytotoxicity to doxorubicin only in PANC-1 cells, while showing lower cytotoxicity toward the non-cancerous HaCaT cell line. The AP1–5 derivatives induce apoptosis, as shown by the presence of morphological hallmarks and activation of caspases-3/7, through a ROS-decreasing mechanism in vitro. Moreover, the network pharmacology and pathway enrichment analyses suggest that AP5 may interact with protein targets involved in PI3K/Akt signaling pathways, such as MAP2K1, MDM2, IGF1, JAK2, IL2 and FGFR1, as well as specific breast cancer and melanoma pathways, suggesting potential mechanisms that should be further investigated. Molecular docking identified MMP-2 as a potential target for AP5; MM-GBSA and the molecular dynamics simulations (100 ns) supported the predicted AP5–MMP-2 interaction during the analyzed trajectory, although longer or independent simulations would provide a more complete assessment of its long-term stability. The ADMET predictions for AP5 suggest favorable drug-like properties, including acceptable intestinal absorption, non-P-glycoprotein substrate, and reduced hepatic clearance compared to Bet. However, the predicted toxicity risks such as DILI, genotoxicity, carcinogenicity and skin sensitization need to be interpreted with caution, as computational models frequently overestimate toxicity for complex structures. Our preliminary assessment on HaCaT cells suggests reduced toxicity of AP5, but further experiments are needed to fully assess the risks. Collectively, these results support the further development of AP5 as a drug candidate in breast cancer models, with future studies focusing primarily on the toxicity evaluation in vivo and further improving anticancer potency through incorporation into nanocarriers. Furthermore, the synthesis and evaluation of additional Bet-bistriazole analogues with alternative linkers, including sulfur-free derivatives, could provide valuable insights into their structure–activity relationships and guide the rational design of more potent derivatives.

## Figures and Tables

**Table 1:** HaCaT, MCF-7, A375 and PANC-1 obtained IC_50_ values (μM) of AP5 and doxorubicin after stimulation for 48 h.

Compound	HaCaT	MCF-7	A375	PANC-1
AP5	>100	7.41	20.36	20.92
Doxorubicin	14.73	1.89	2.38	49.74

**Table 2:** Target counts per compound per cancer type identified via PharmMapper.

Compound	Total Targets (Fit Score > 3, z’-Score > 1)	Melanoma	Breast Cancer	Pancreatic Cancer
Bet	74	5	8	5
AP1	64	6	9	7
AP2	66	7	10	7
AP3	68	5	8	6
AP4	58	5	10	7
AP5	104	7	10	8

**Table 3:** Docking scores of AP5 and native ligands against the selected protein targets.

Compound ID	MMP-2 1HOV	IL-2 1QVN	MEK1 1S9J	MDM2/HDM2 1T4E	ERα 1XP9	PR 1E3K
AP5	−9.1	−7.2	−6.9	−7.4	−3.0	17.0
Native ligand	−8.4	−9.9	−8.9	−9.7	−11.8	−11.2

**Table 4:** Prime MM-GBSA binding free energy and main energetic contributions for the AP5–MMP-2 complex. Energy values are expressed in kcal/mol. Negative values indicate favorable energetic contributions, whereas positive values indicate energetic penalties.

MM-GBSA Parameter	Value (kcal/mol)
ΔGbind total	−43.81
vdW	−28.81
Coulomb	−25.58
H-bond	−2.23
Lipophilic	−9.27
Packing	−3.22
Solv_GB	+26.10
Ligand strain energy	+6.48
Ligand efficiency	−0.88

**Table 5:** Key predicted ADMET parameters of Bet and AP5, according to ADMETlab 3.0. HIA: human intestinal absorption; P-gp: P-glycoprotein; BBB: blood–brain barrier; PPB: plasma protein binding; HLM: human liver microsomal stability; CYP: cytochrome P450; CL: plasma clearance; T½: half-life; hERG: human ether-à-go-go-related gene; DILI: drug-induced liver injury; AMES: Ames mutagenicity test.

Property	Bet	AP5
Absorption		
Caco-2 permeability	acceptable	acceptable
HIA	high	high
P-gp substrate	substrate	inhibitor
Distribution		
BBB penetration	high	low
PPB (%)	high	high
Metabolism		
HLM stability	moderate stability	high stability
CYP2C19 substrate	yes	weak
CYP3A4 substrate	no	yes
Excretion		
Cl plasma	moderate	low
T½	very short	short
Toxicity		
hERG blocker	low risk	low risk
DILI risk	moderate risk	high risk
AMES mutagenicity	borderline	low risk
Carcinogenicity	high risk	high risk
Genotoxicity	moderate risk	high risk
Nephrotoxicity	moderate risk	high risk
Skin sensitization	high risk	high risk
Hepatotoxicity	moderate risk	borderline
Rat oral acute toxicity	low	low

**Table 6:** Protein targets and docking grid parameters used for AP5 molecular docking.

Protein Target	PDB ID	Center Coordinates (x, y, z)	Grid Box Size (x, y, z)
MMP-2	1HOV	(11.1020, 21.2199, 15.0563)	(25.0000, 25.0000, 25.0000)
IL-2	1QVN	(49.7095, 12.7484, 3.9279)	(15.9720, 28.8297, 17.0339)
MEK1	1S9J	(31.2736, 28.0970, 38.7506)	(18.5627, 14.2806, 14.2806)
MDM2/HDM2	1T4E	(43.0832, 12.2686, 30.8779)	(18.2618, 20.1924, 19.2600)
ERα	1XP9	(30.7472, −1.7108, 24.7800)	(18.5627, 14.2806, 16.4588)
PR	1E3K	(28.8373, −8.8372, 8.3729)	(20.4711, 20.4711, 20.4711)

## Data Availability

The original contributions presented in this study are included in the article/Supplementary Material. Further inquiries can be directed to the corresponding author.

## Supplementary Materials

The following supporting information can be downloaded at https://www.mdpi.com/article/10.3390/ijms27135960/s1.
