# Supplementary material for: Novel C3/C28-bis-1,2,4-Triazolyl-sulfanylacetate-betulin Derivatives: Synthesis and Evaluation of Anticancer Potential
Source: Int J Mol Sci. 2026 Jul 2;27(13):5960. doi: 10.3390/ijms27135960 (PMC13361304; doi:10.3390/ijms27135960)
Supplement: Supplementary file 1 [file ijms-27-05960-s001.zip › ijms-4388316-supplementary.pdf]

SUPPLEMENTARY FILE

Novel C3/C28-bis-1,2,4-triazolyl-sulfanylacetate-betulin derivatives: synthesis and evaluation of anticancer potential

Authors: Alexandra Prodea[1,2], Marius Mioc[1,2], Andreea Munteanu[1,2], Alexandra Mioc[1,2], Nicoleta Anamaria Paşcalău[3,*], Bogdan-Ionuț Mara[1,2,4], Elisabeta Atyim[1,2], Mihaela Balan-Porcarasu[5], Roxana Racoviceanu[1,2], Codruța Șoica[1,2]

Affiliation

[1] Faculty of Pharmacy, "Victor Babes" University of Medicine and Pharmacy, Eftimie Murgu Square, No. 2, 300041 Timisoara, Romania

[2] Research Center for Experimental Pharmacology and Drug Design (X-Pharm Design), "Victor Babes" University of Medicine and Pharmacy, Eftimie Murgu Square, No. 2, 300041 Timișoara, Romania

[3] Department of Psycho Neuroscience and Recovery, Faculty of Medicine and Pharmacy, University of Ora-12 dea, 410087 Oradea, Romania: N.A.P (nicoleta.pascalau@didactic.uoradea.ro)

[4] Coriolan Dragulescu Institute of Chemistry, Romanian Academy, Bv. M. Viteazu, No. 24, 300223 Timisoara, Romania

[5] Institute of Macromolecular Chemistry 'Petru Poni', 700487 Iasi, Romania

Contents

List of figures

Figure S1. ^{1}H NMR spectrum of betulin-3,28-O-di(chloroacetate) (Bet-(ClAc)2)
Figure S2. ^{13}C NMR spectrum of Bet-(ClAc)2
Figure S3. FTIR spectrum of (Bet-(ClAc)2
Figure S4. ^{1}H NMR spectrum of betulin-3,28-O-diyl-bis[5-(4-chlorophenyl)-1H-1,2,4-triazol-3-yl)sulfanylacetate]) (AP1)
Figure S5. ^{13}C NMR spectrum of AP1
Figure S6. FTIR spectrum of AP1
Figure S7. ^{1}H NMR spectrum of betulin-3,28-O-diyl-bis[5-(4-methoxyphenyl)-1H-1,2,4-triazol-3-yl)sulfanylacetate]) (AP2)
Figure S8. ^{13}C NMR spectrum of AP2
Figure S9. FTIR spectrum of AP2
Figure S10. ^{1}H NMR spectrum of betulin-3,28-O-diyl-bis[5-(phenyl)-1H-1,2,4-triazol-3-yl)sulfanylacetate]) (AP3)
Figure S11. ^{13}C NMR spectrum of AP3
Figure S12. FTIR spectrum of AP3
Figure S13. ^{1}H NMR spectrum of betulin-3,28–O-diyl-bis[5-(4-dimethylaminophenyl)-1H-1,2,4-triazol-3-yl)sulfanylacetate]) (AP4)
Figure S14. ^{13}C NMR spectrum of AP4
Figure S15. FTIR spectrum of AP4
Figure S16. ^{1}H NMR spectrum of betulin-3,28-O-diyl-bis[5-(1H-1,2,4-triazol-3-yl)sulfanylacetate] (AP5)
Figure S17. ^{13}C NMR spectrum of AP5
Figure S18. FTIR spectrum of AP5
Figure S19. HaCaT cell viability after 48h treatment with AP1-AP5 (1, 5, 10, 50 and 100 μM) and doxorubicin.
Figure S20. MCF-7 cell viability after 48h treatment with AP1-AP5 (1, 5, 10, 50 and 100 μM) and doxorubicin.
Figure S21. A375 cell viability after 48h treatment with AP1-AP5 (1, 5, 10, 50 and 100 μM) and doxorubicin.
Figure S22. PANC-1 cell viability after 48h treatment with AP1-AP5 (1, 5, 10, 50 and 100 μM) and doxorubicin.
Figure S23. The effects of AP1, AP2 (A), AP3, AP4 and AP5 (B) on HaCaT cells' morphology at both 0 h and 48 h after stimulation.
Figure S24. The effects of AP1, AP2 (A), AP3, AP4 and AP5 (B) on A375 cells' morphology at both 0 h and 48 h after stimulation.
Figure S25. The effects of AP1, AP2 (A), AP3, AP4 and AP5 (B) on MCF-7 cells' morphology at both 0 h and 48 h after stimulation.
Figure S26. The effects of AP1, AP2 (A), AP3, AP4 and AP5 (B) on PANC-1 cells' morphology at both 0 h and 48 h after stimulation.
Figure S27. Enlarged Figure 3, Panel A (Ctr, STZ, AP1, AP2)

Figure S28. Enlarged Figure 3, Panel B (AP3, AP4 and AP5)
Figure S29. Enlarged Figure 4, Panel A (Ctr, STZ, AP1, AP2)
Figure S30. Enlarged Figure 4, Panel B (AP3, AP4 and AP5)
Figure S31. Enlarged Figure 5, Panel A (Ctr, STZ, AP1, AP2)
Figure S32. Enlarged Figure 5, Panel B (AP3, AP4 and AP5)
Figure S33. Enlarged Figure 6, Panel A (Ctr, STZ, AP1, AP2)
Figure S34. Enlarged Figure 6, Panel B (AP3, AP4 and AP5)

List of tables

Table S1. Targets identified through the Pharmamapper platform for Bet and AP1-5
Table S2. Protein coding targets for melanoma, breast and pancreatic cancer identified through the Genecards database.
Table S3. Pharmacologically predicted common targets of Bet and AP1–5 overlapping with cancer-specific targets
Table S4. STRING database interaction scores for cancer-associated predicted targets
Table S5. String enrichment analysis results
Table S6. Enrichr enrichment analysis results
Table S7. Physicochemical and ADMET predictions for Bet and AP5 according to ADMETlab 3.0.

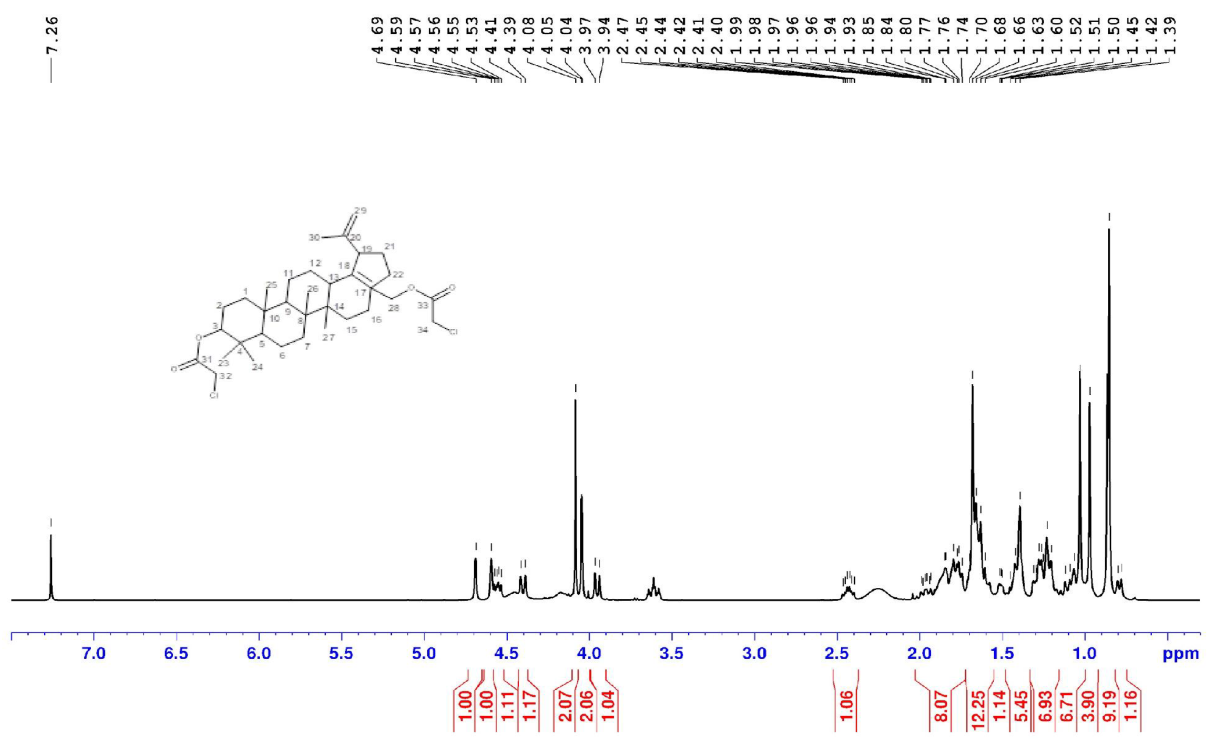

Figure S1. ^{1}H NMR spectrum of betulin-3,28-O-di(chloroacetate) (Bet-(ClAc)2)

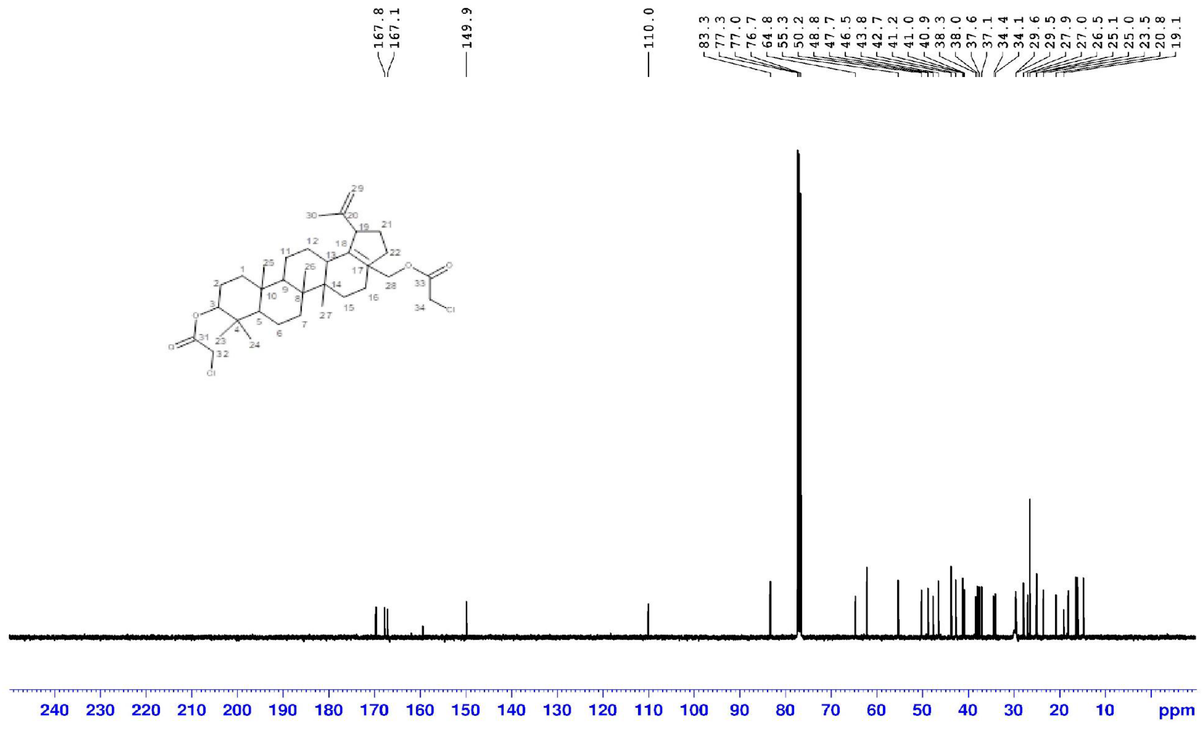

Figure S2.^{13}C NMR spectrum of Bet-(ClAc)2

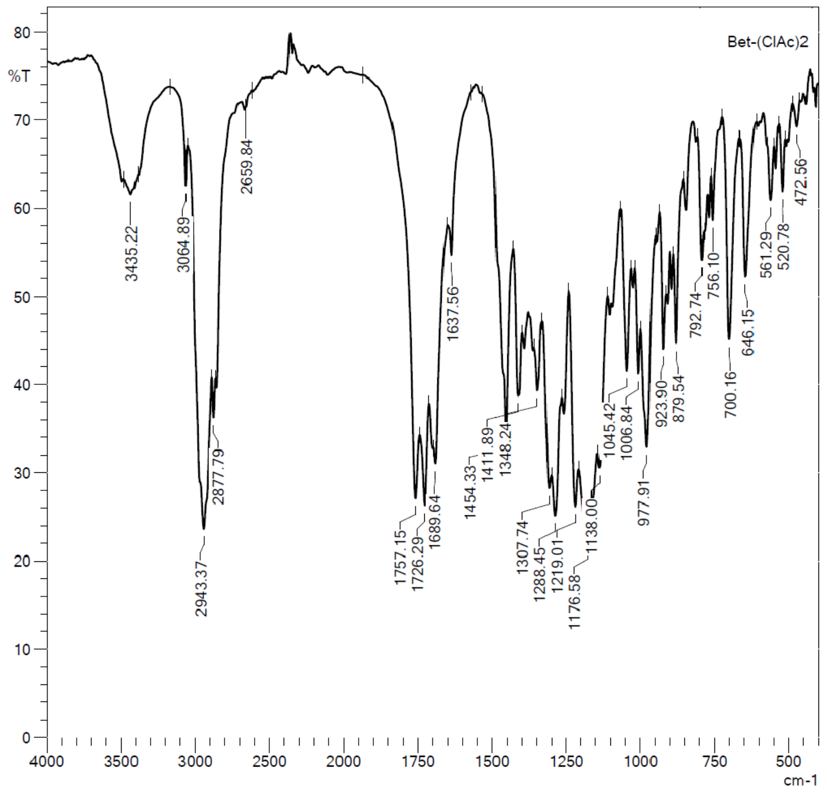

Figure S3. FTIR spectrum of Bet-(ClAc)2

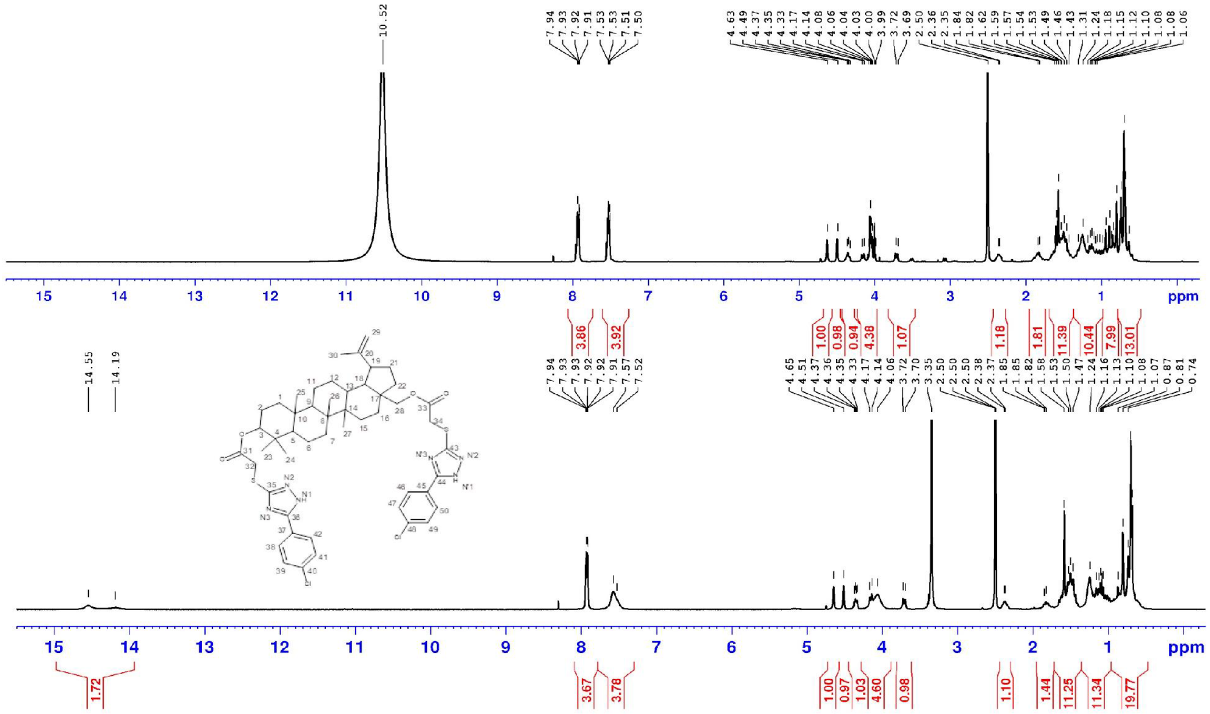

Figure S4. ^{1}H NMR spectrum of betulin-3,28-O-diyl-bis[5-(4-chlorophenyl)-1H-1,2,4-triazol-3-yl)sulfanylacetate]) (AP1)

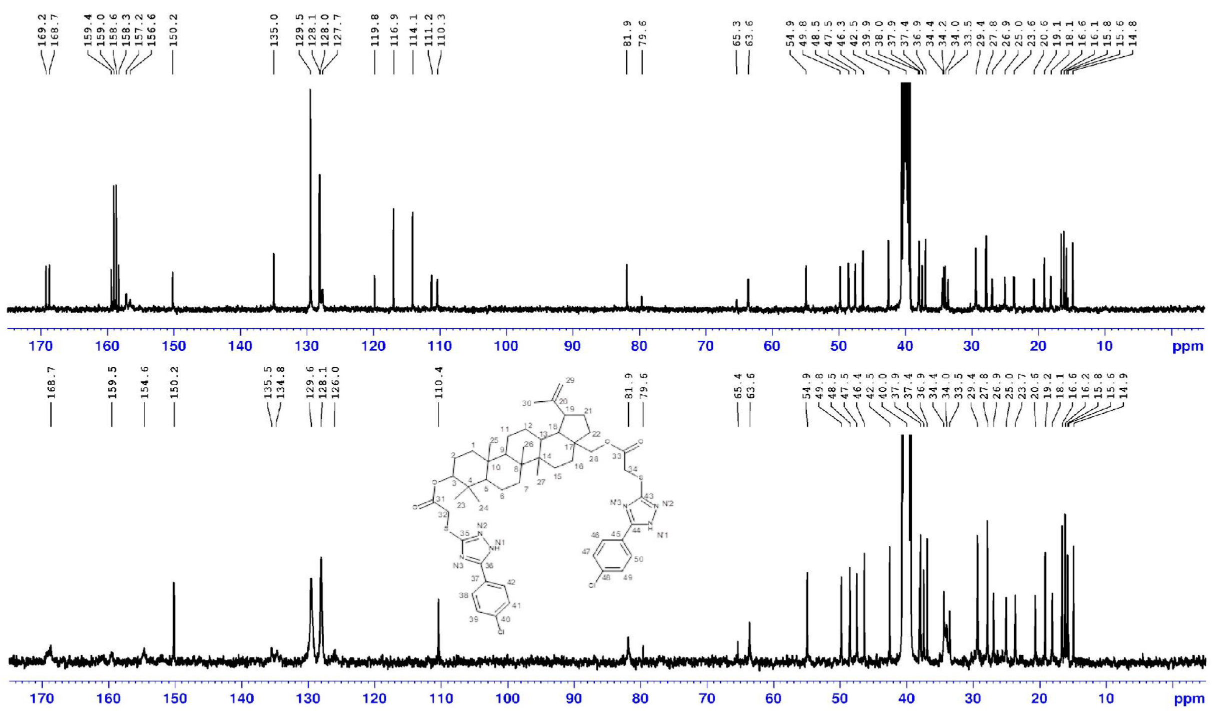

Figure S5.^{13}C NMR spectrum of AP1

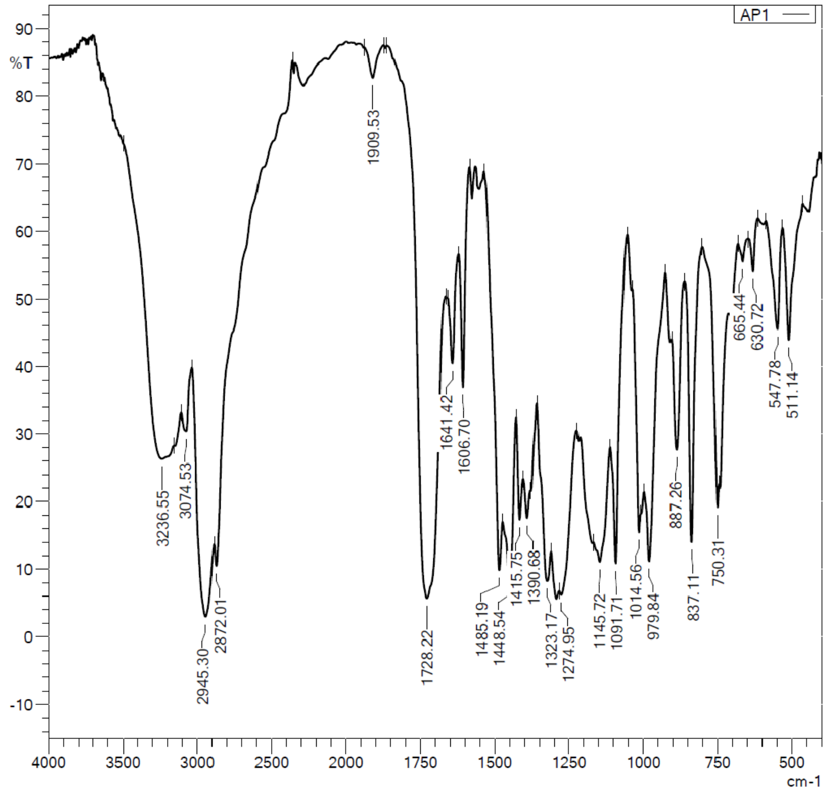

Figure S6. FTIR spectrum of AP1

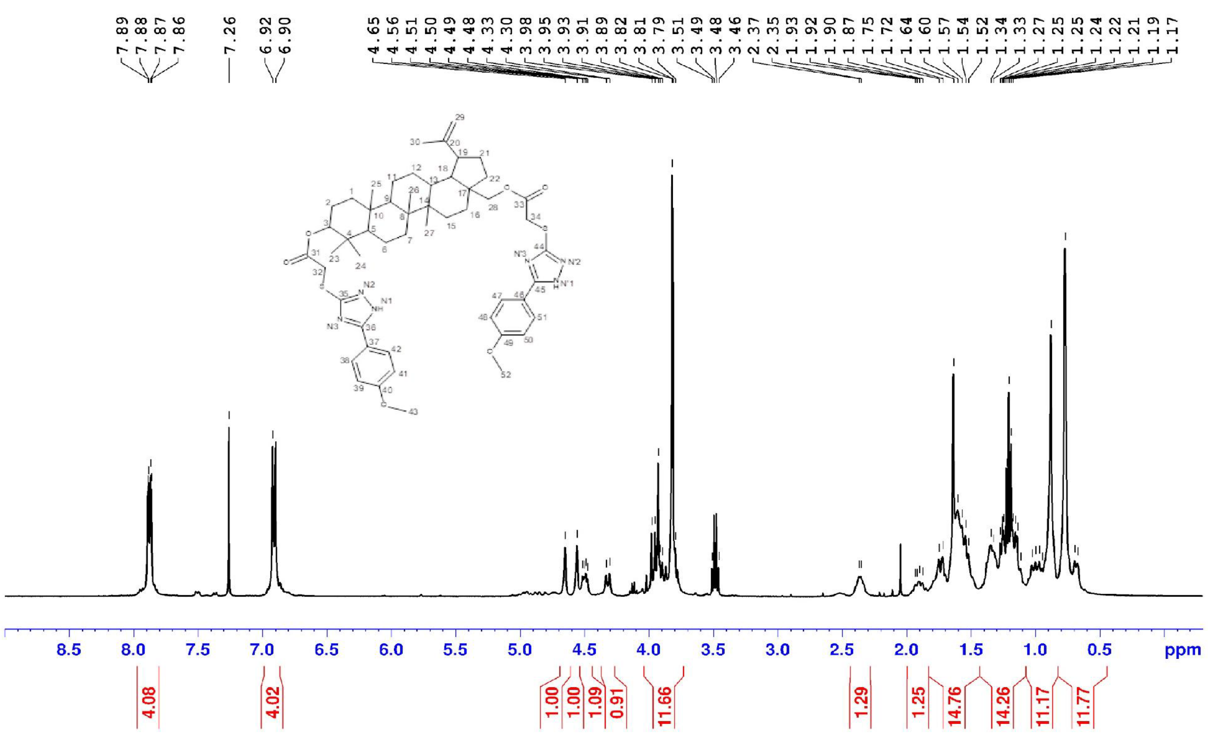

Figure S7. ^{1}H NMR spectrum of betulin-3,28-O-diyl-bis[5-(4-methoxyphenyl)-1H-1,2,4-triazol-3-yl)sulfanylacetate]) (AP2)

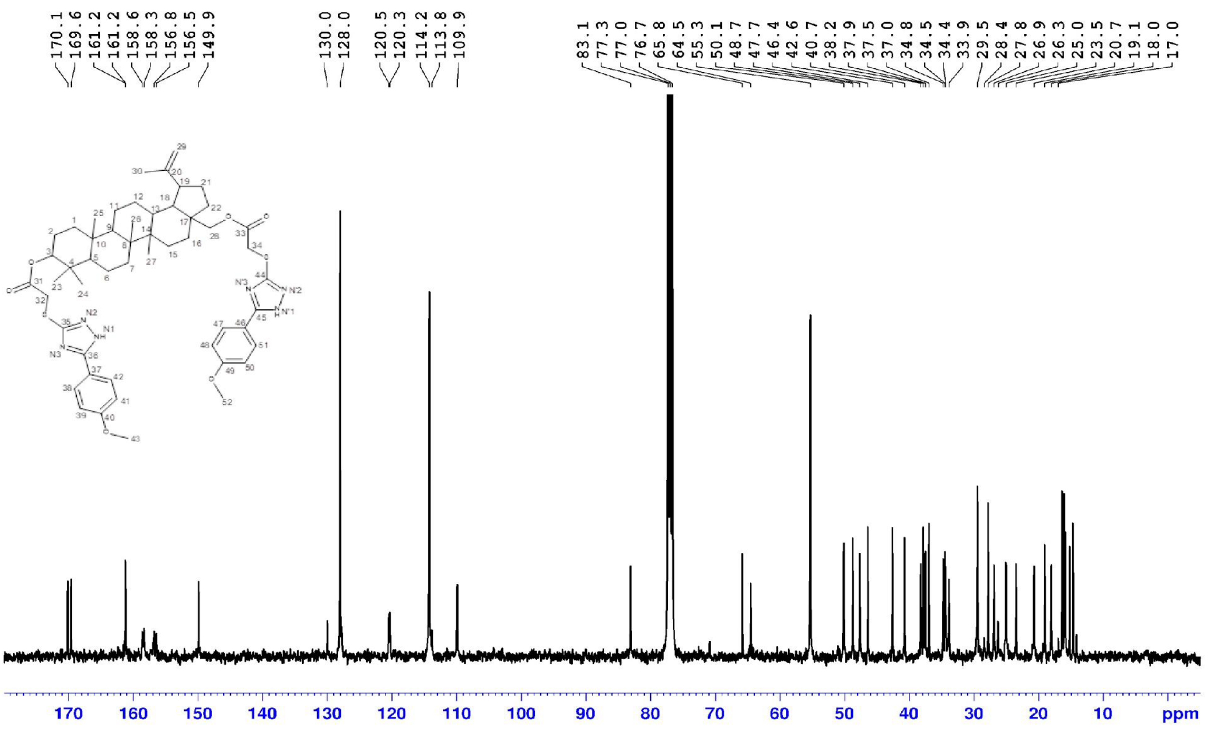

Figure S8.^{13}C NMR spectrum of AP2

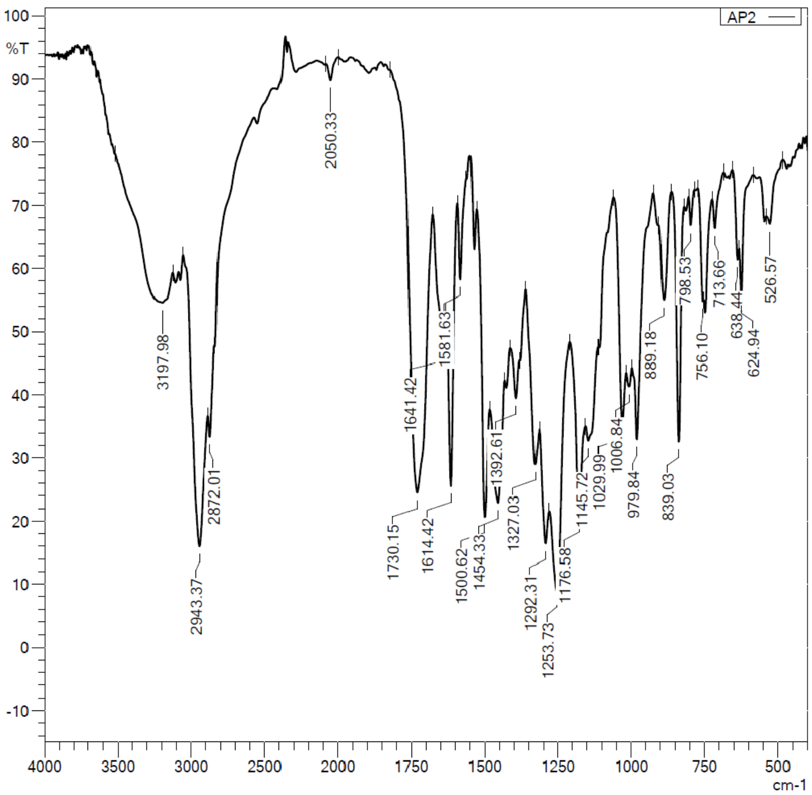

Figure S9. FTIR spectrum of AP2

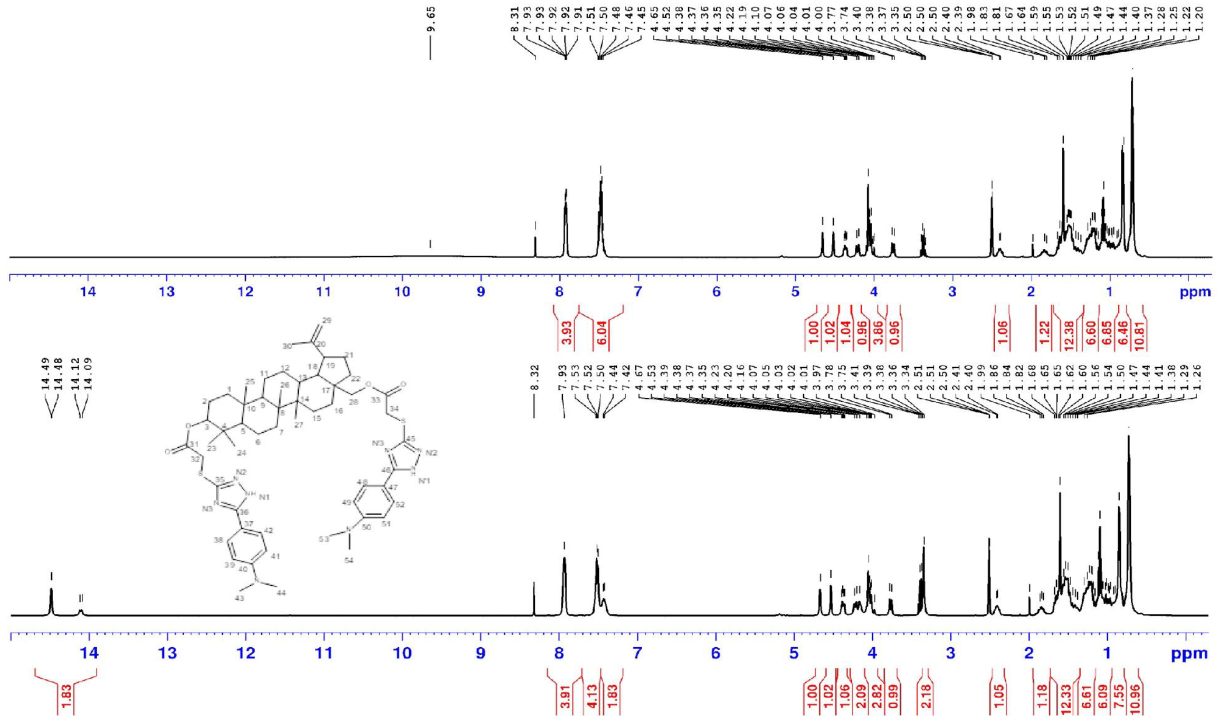

Figure S10. ^{1}H NMR spectrum of betulin-3,28-O-diyl-bis[5-(phenyl)-1H-1,2,4-triazol-3-yl)sulfanylacetate]) (AP3)

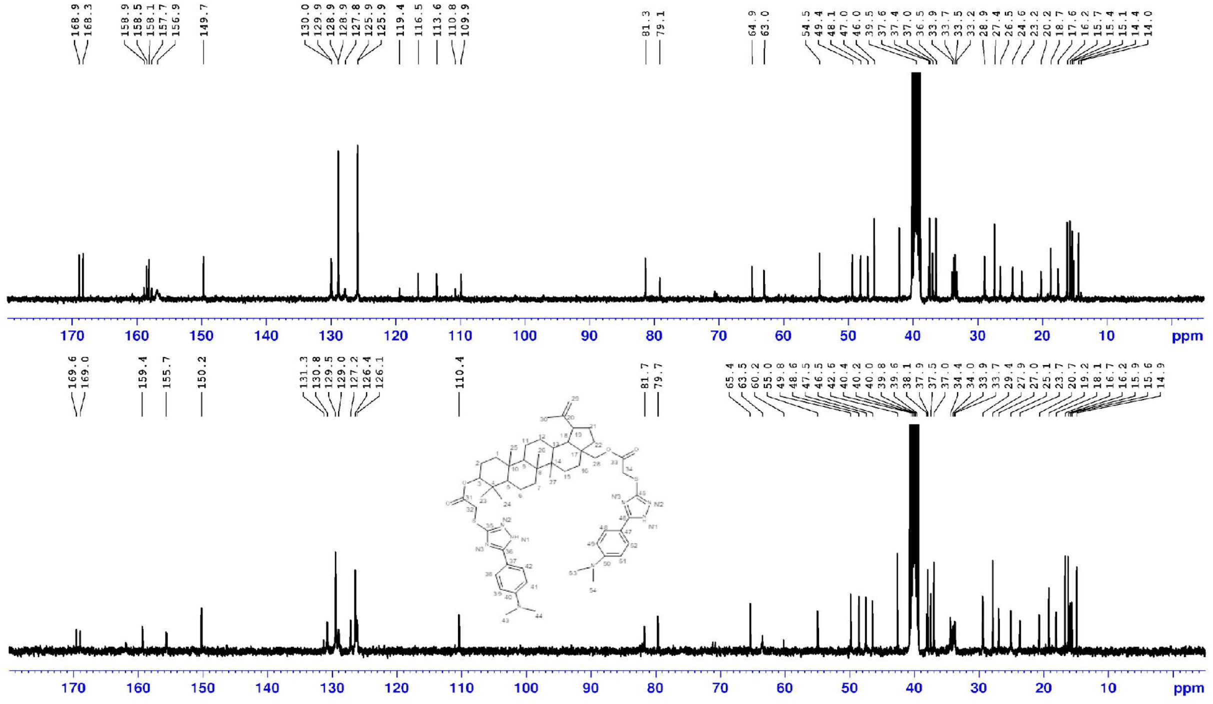

Figure S11.^{13}C NMR spectrum of AP3

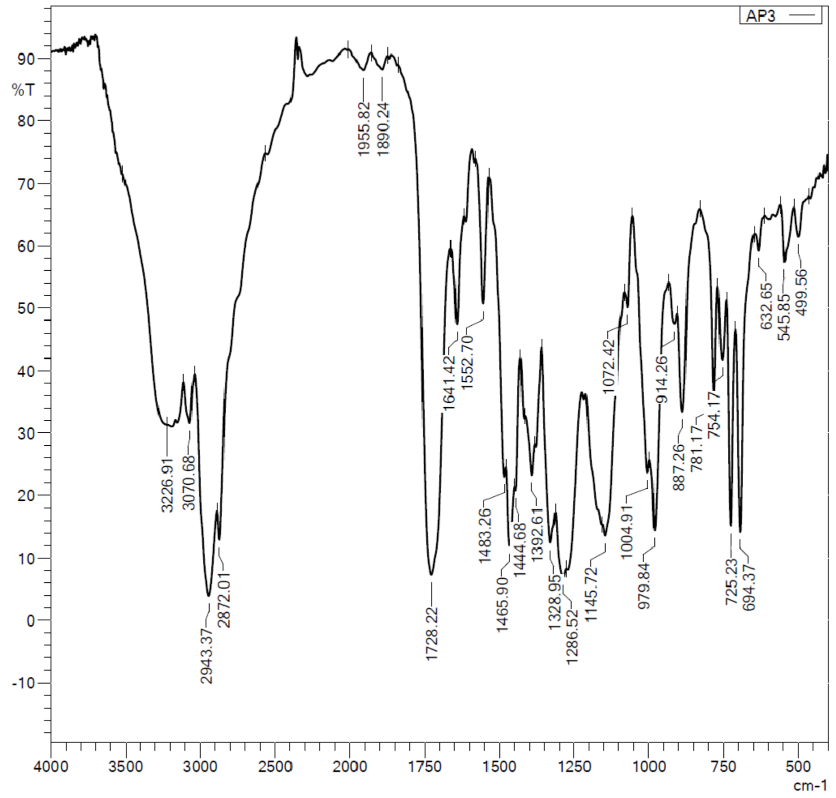

Figure S12. FTIR spectrum of AP3

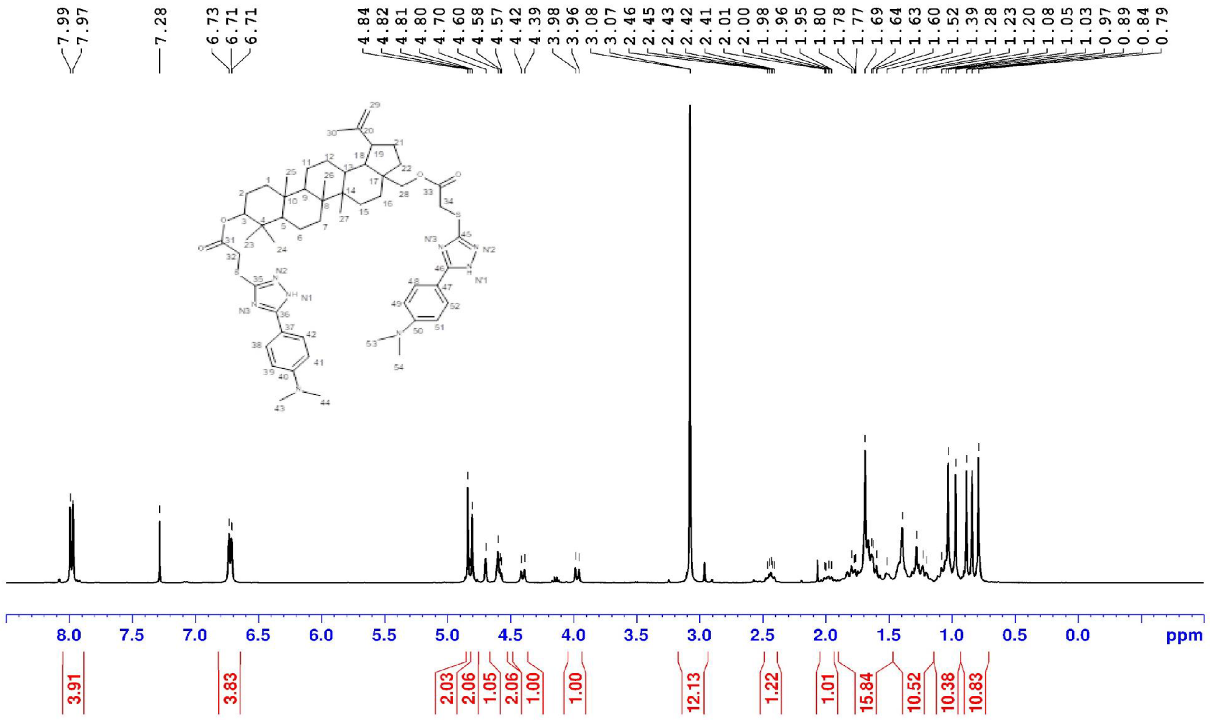

Figure S13. ^{1}H NMR spectrum of betulin-3,28–O-diyl-bis[5-(4-dimethylaminophenyl)-1H-1,2,4-triazol-3-yl)sulfanylacetate]) (AP4)

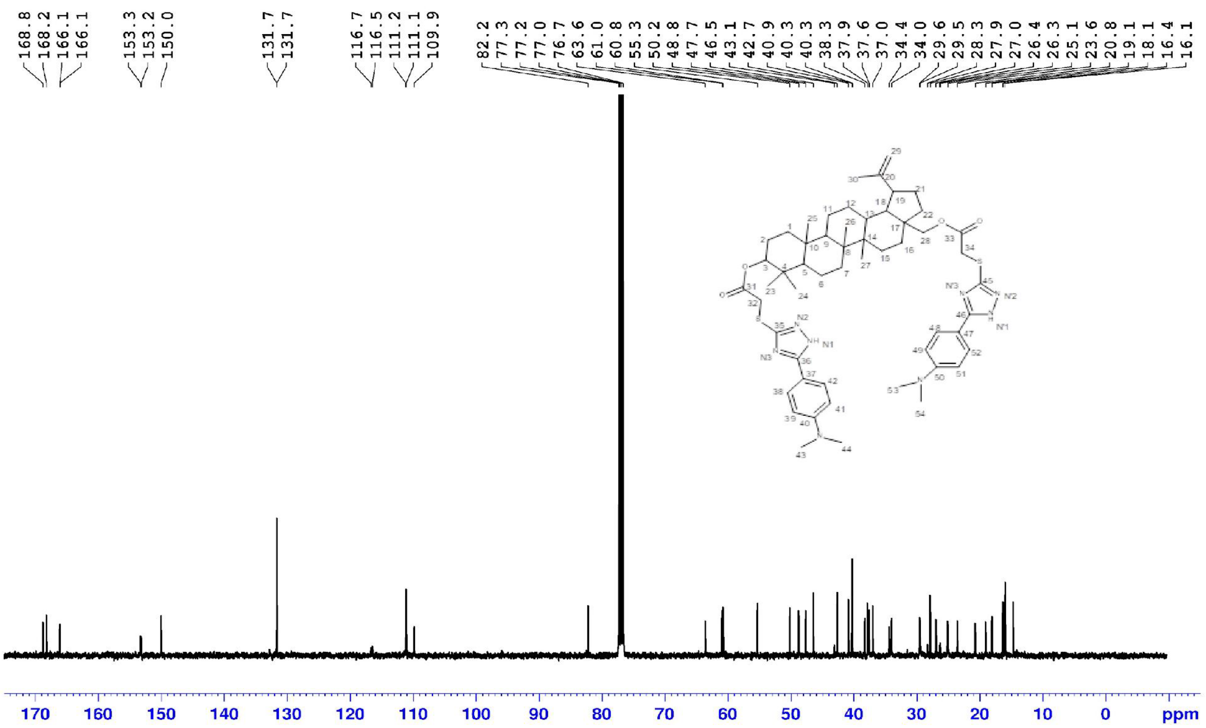

Figure S14.^{13}C NMR spectrum of AP4

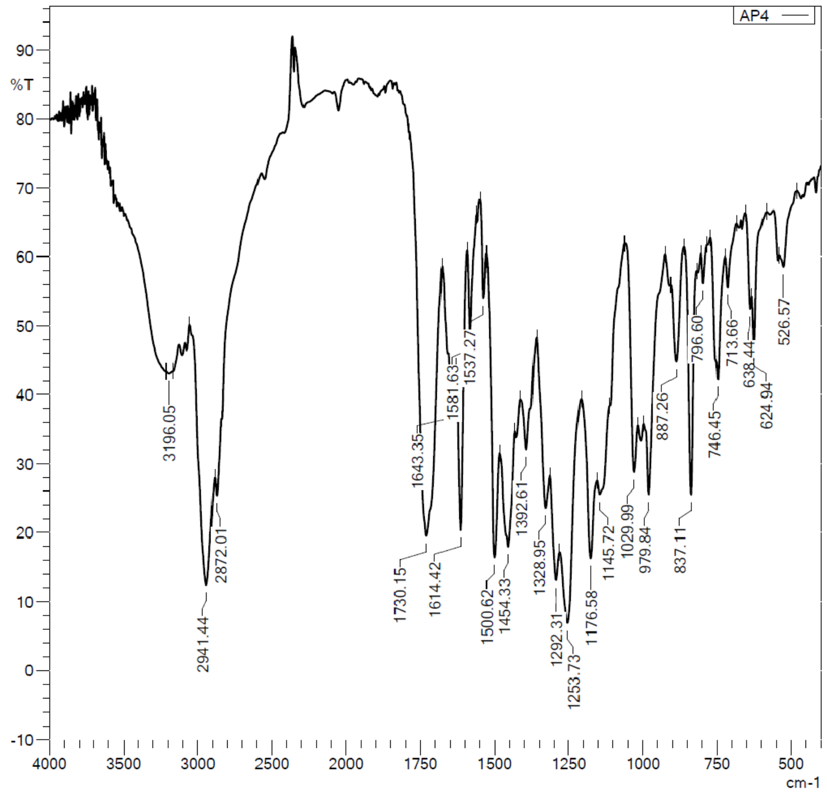

Figure S15. FTIR spectrum of AP4

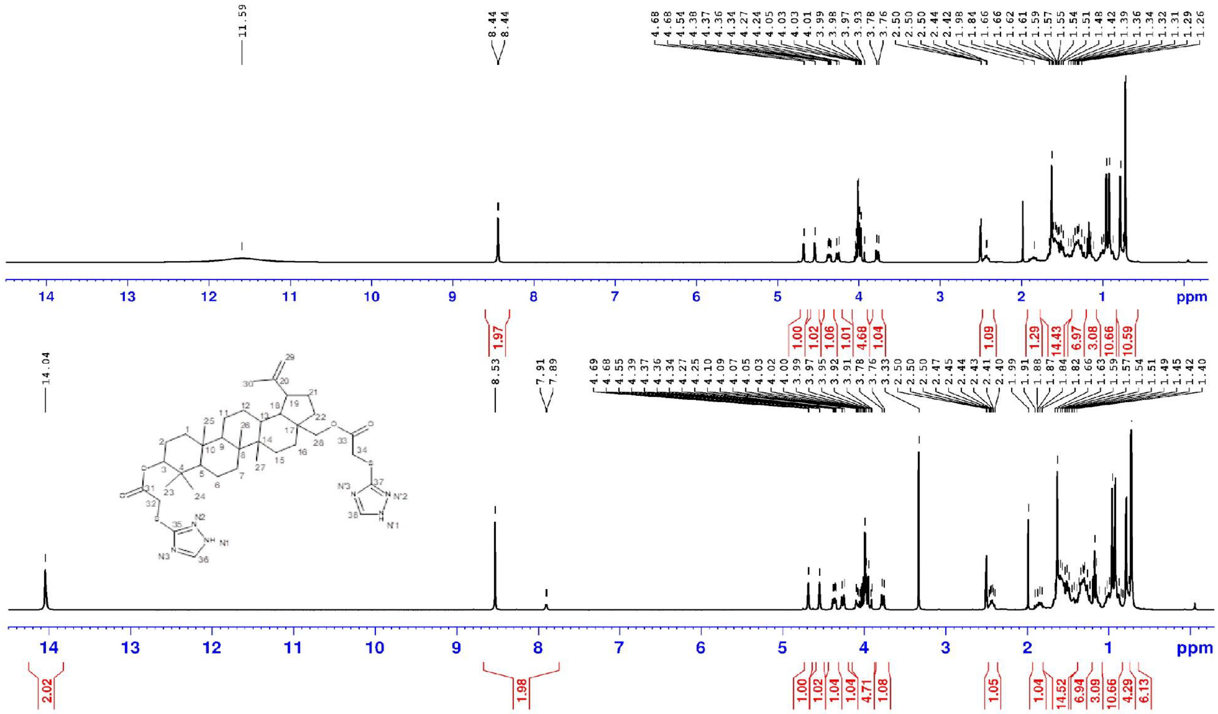

Figure S16. ^{1}H NMR spectrum of betulin-3,28-O-diyl-bis[5-(1H-1,2,4-triazol-3-yl)sulfanylacetate] (AP5)

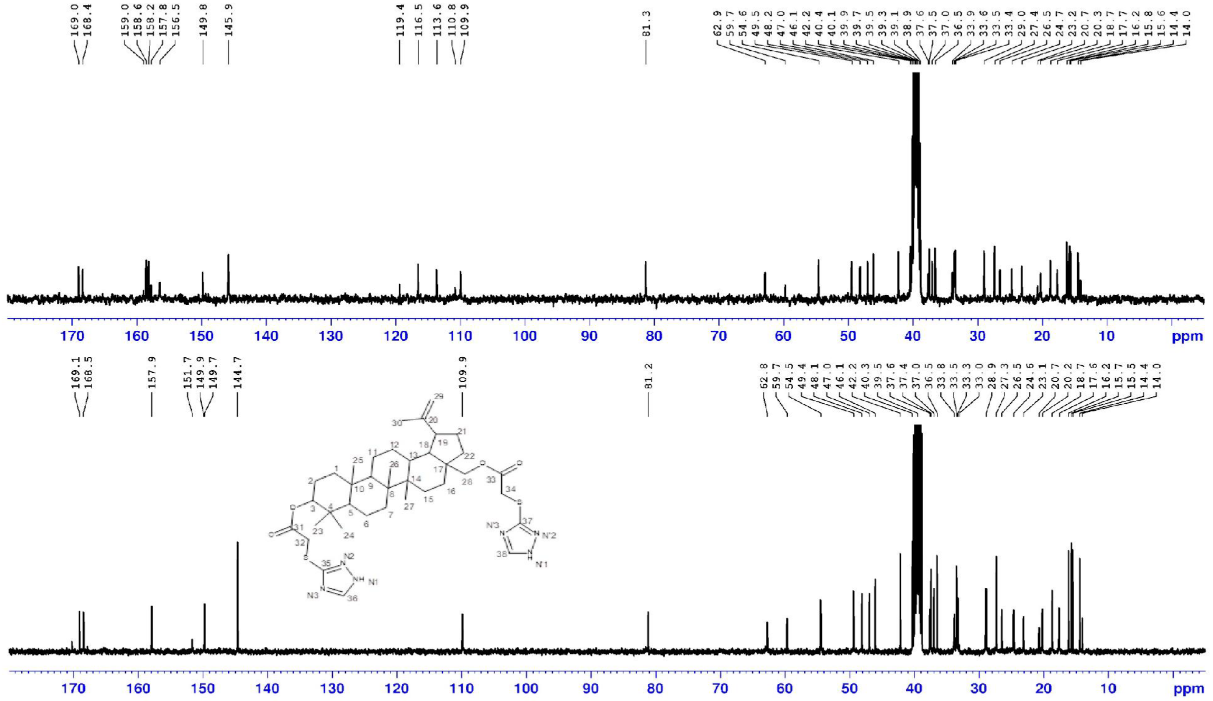

Figure S17.^{13}C NMR spectrum of AP5

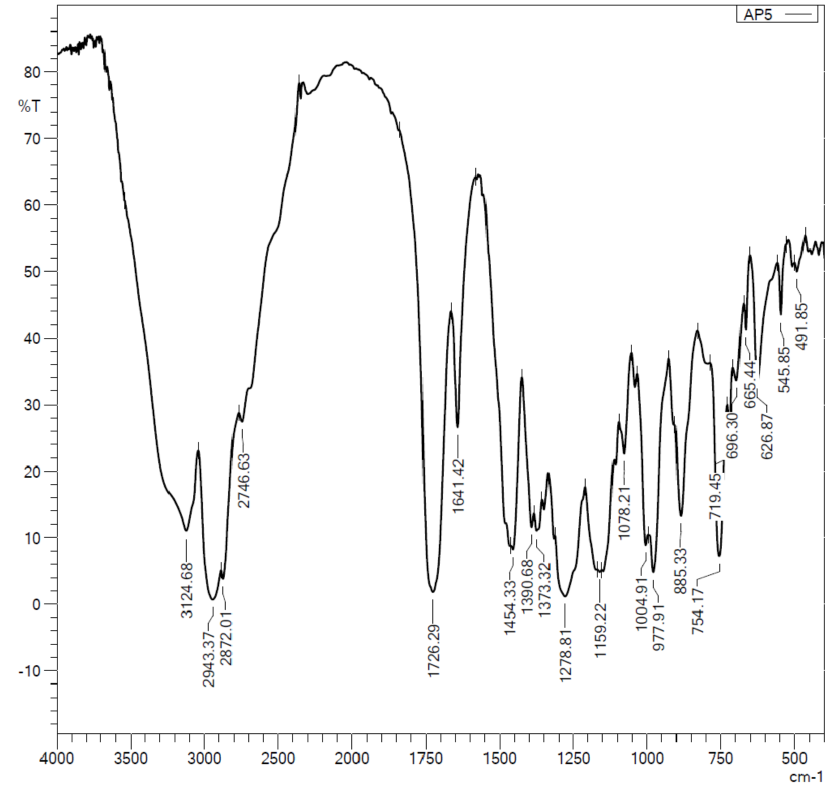

Figure S18. FTIR spectrum of AP5

HaCaT

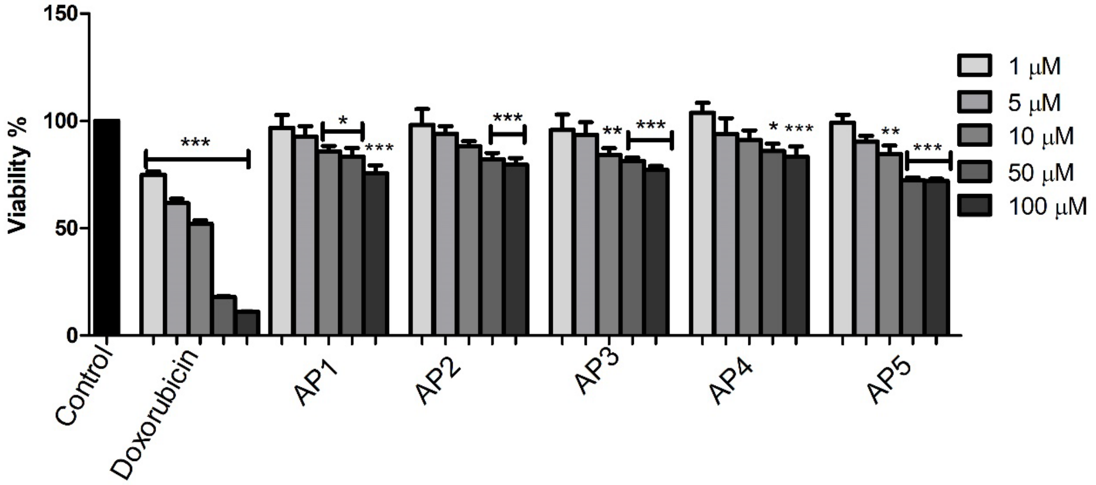

Figure S19. HaCaT cell viability after 48h treatment with AP1-AP5 (1, 5, 10, 50 and 100 μM) and doxorubicin. The results are expressed as percentages of viability relative to the control (100%). The data represent mean values ± SD of three independent experiments performed in triplicate. The statistical differences vs control were determined using the one-way ANOVA analysis followed by Dunnett's comparison post-test (* $p < 0.05$, ** $p < 0.01$, and *** $p < 0.001$).

MCF-7

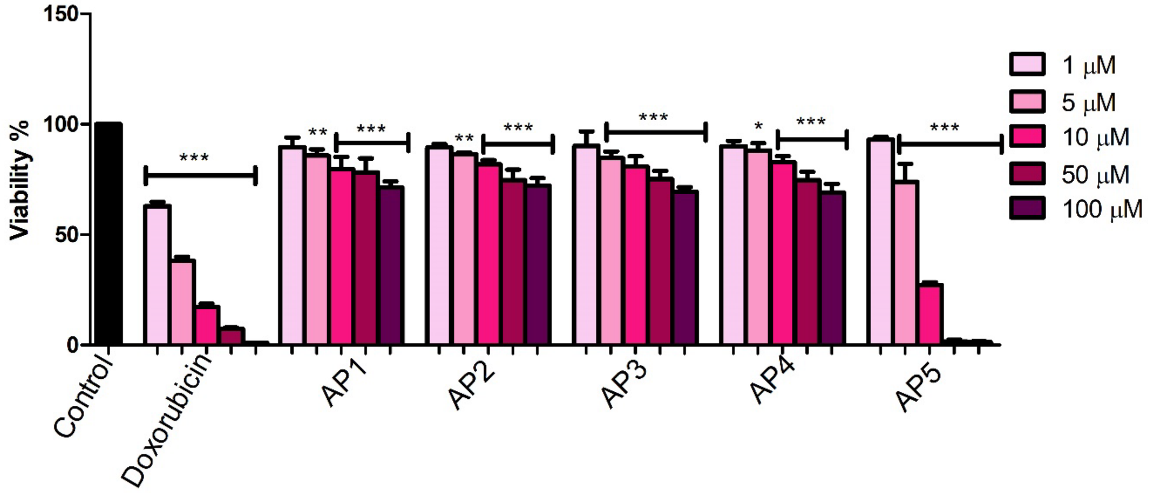

Figure S20. MCF-7 cell viability after 48h treatment with AP1-AP5 (1, 5, 10, 50 and 100 μM) and doxorubicin. The results are expressed as percentages of viability relative to the control (100%). The data represent mean values ± SD of three independent experiments performed in triplicate. The statistical differ-ences vs control were determined using the one-way ANOVA analysis followed by Dunnett's comparison post-test (* $p < 0.05$, ** $p < 0.01$, and *** $p < 0.001$).

A375

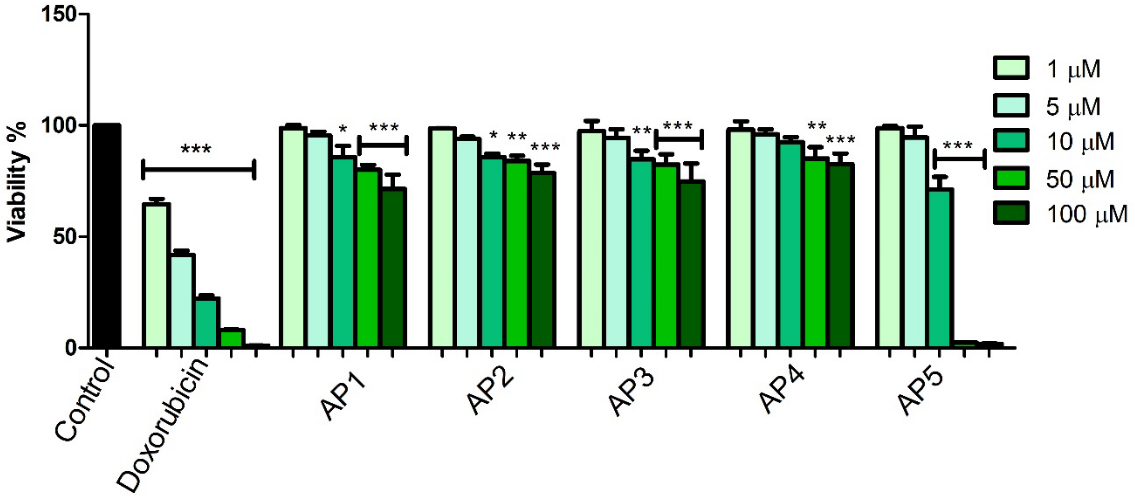

Figure S21. A375 cell viability after 48h treatment with AP1-AP5 (1, 5, 10, 50 and 100 μM) and doxorubicin. The results are expressed as percentages of viability relative to the control (100%). The data represent mean values ± SD of three independent experiments performed in triplicate. The statistical differ-ences vs control were determined using the one-way ANOVA analysis followed by Dunnett's comparison post-test (* $p < 0.05$, ** $p < 0.01$, and *** $p < 0.001$).

PANC-1

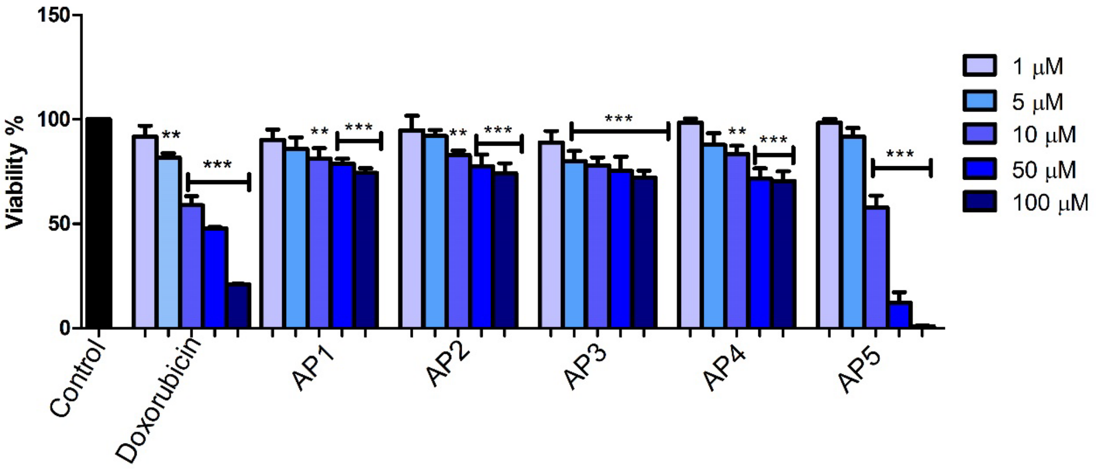

Figure S22. PANC-1 cell viability after 48h treatment with AP1-AP5 (1, 5, 10, 50 and 100 μM) and doxorubicin. The results are expressed as percentages of viability relative to the control (100%). The data represent mean values ± SD of three independent experiments performed in triplicate. The statistical differences vs control were determined using the one-way ANOVA analysis followed by Dunnett's comparison post-test (* $p < 0.05$, ** $p < 0.01$, and *** $p < 0.001$).

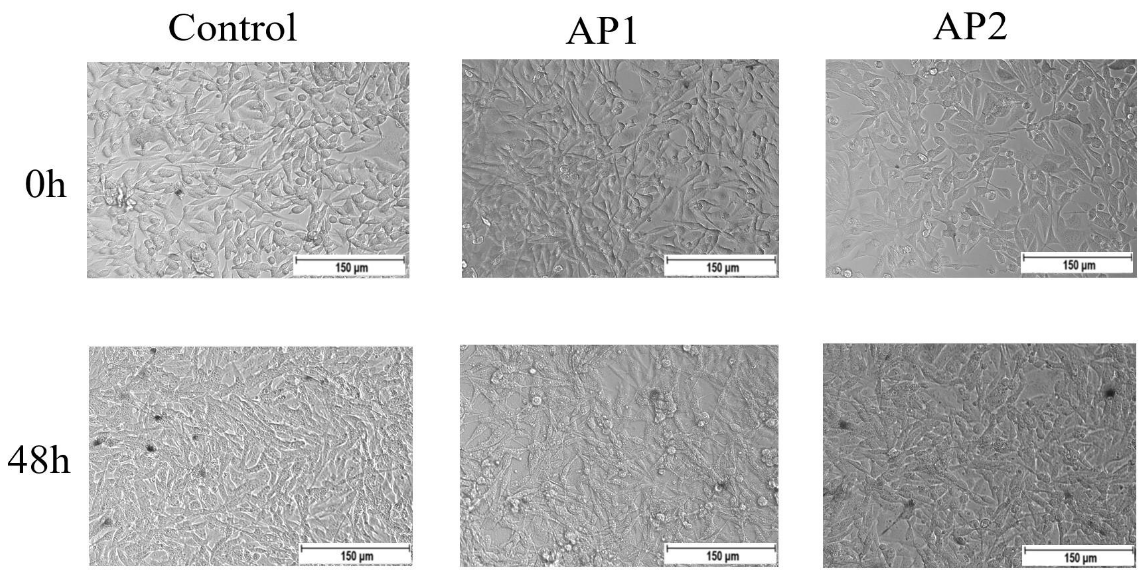

(A)

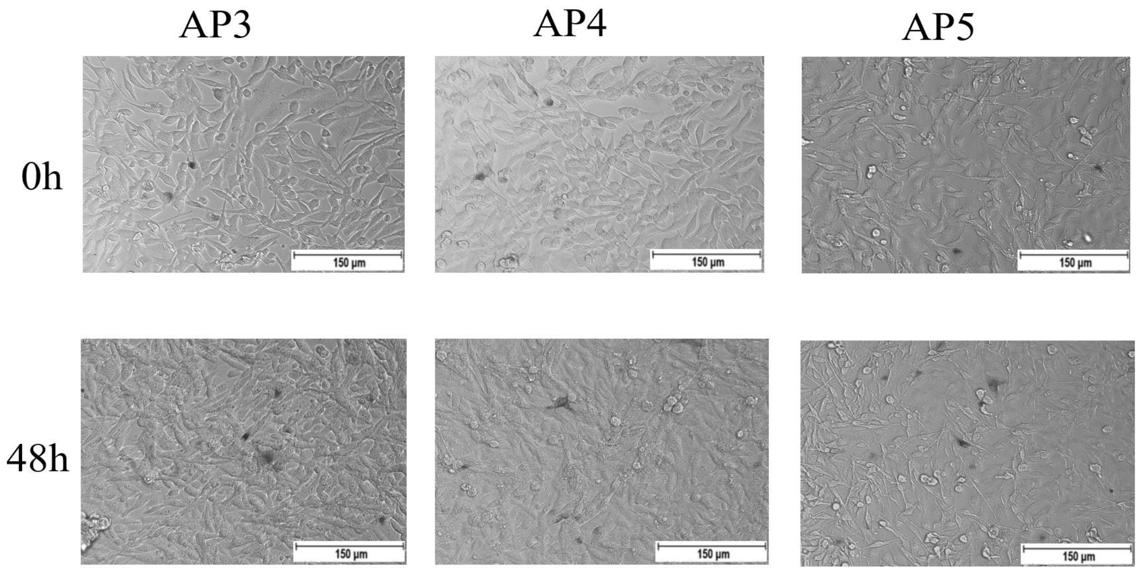

(B)

Figure S23. The effects of AP1, AP2 (A), AP3, AP4 and AP5 (B) on HaCaT cells' morphology at both 0 h and 48 h after stimulation; the scale bar was 150 µm.

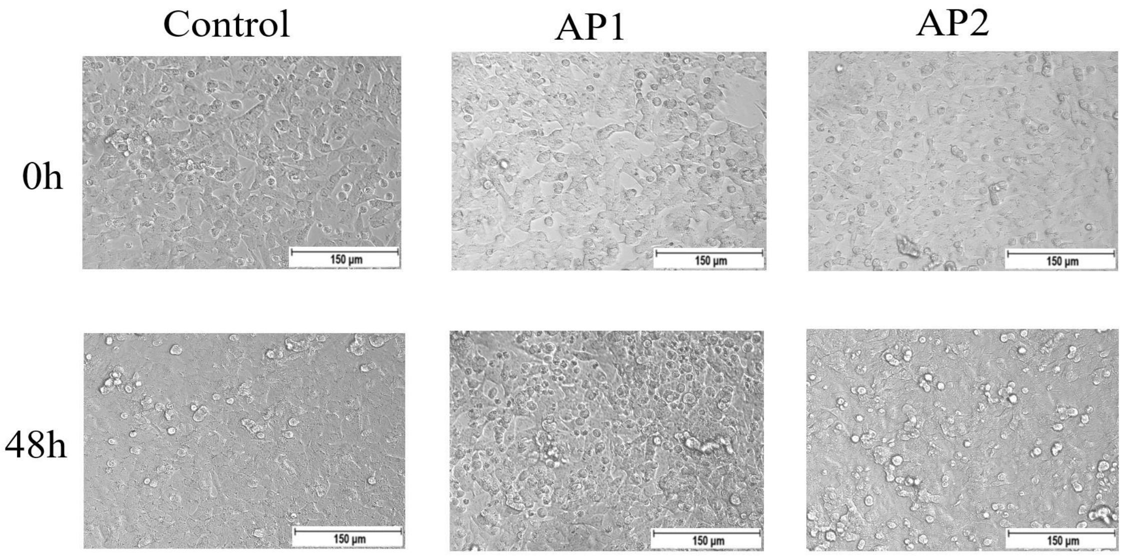

(**A**)

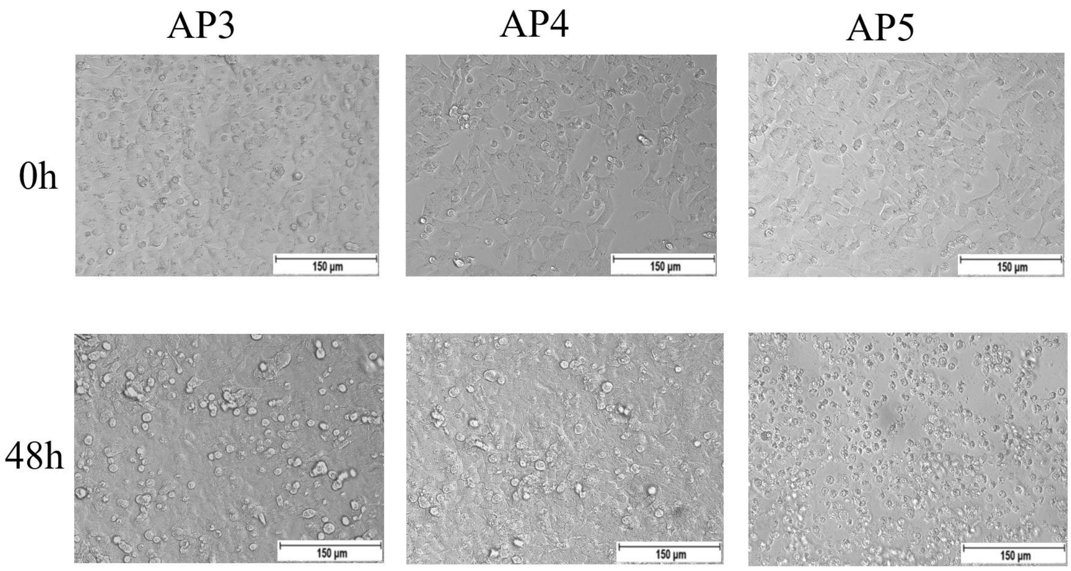

(**B**)

Figure S24. The effects of AP1, AP2 (A), AP3, AP4 and AP5 (B) on A375 cells' morphology at both 0 h and 48 h after stimulation; the scale bar was 150 μm.

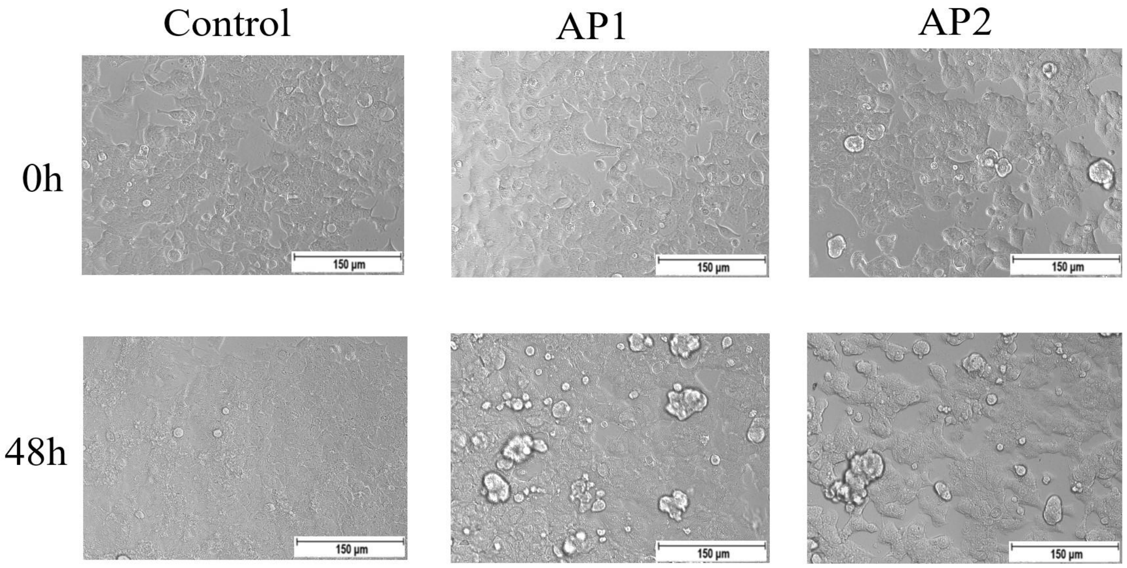

(A)

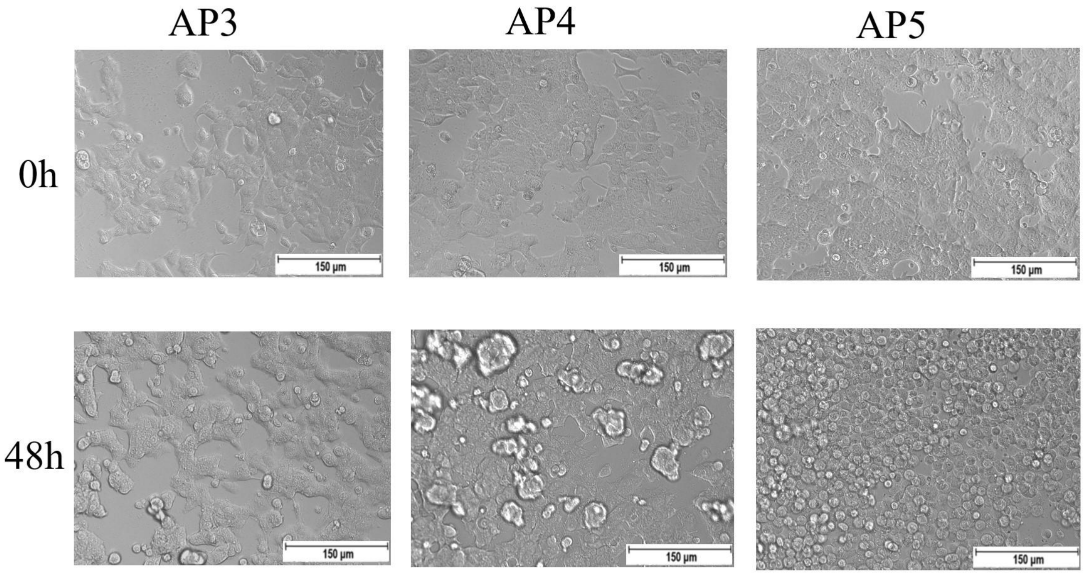

(B)

Figure S25. The effects of AP1, AP2 (A), AP3, AP4 and AP5 (B) on MCF-7 cells' morphology at both 0 h and 48 h after stimulation; the scale bar was 150 µm.

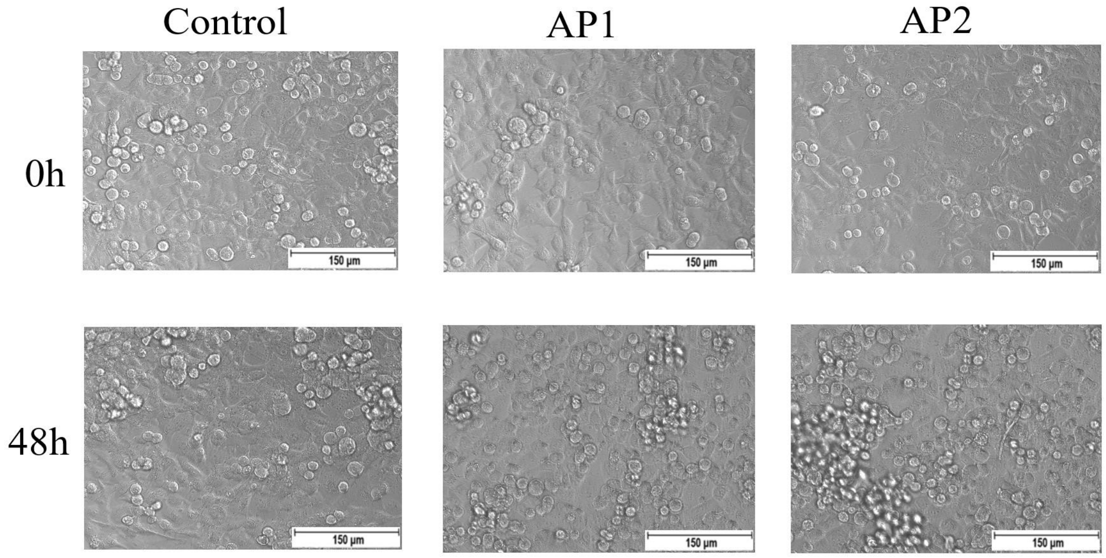

(**A**)

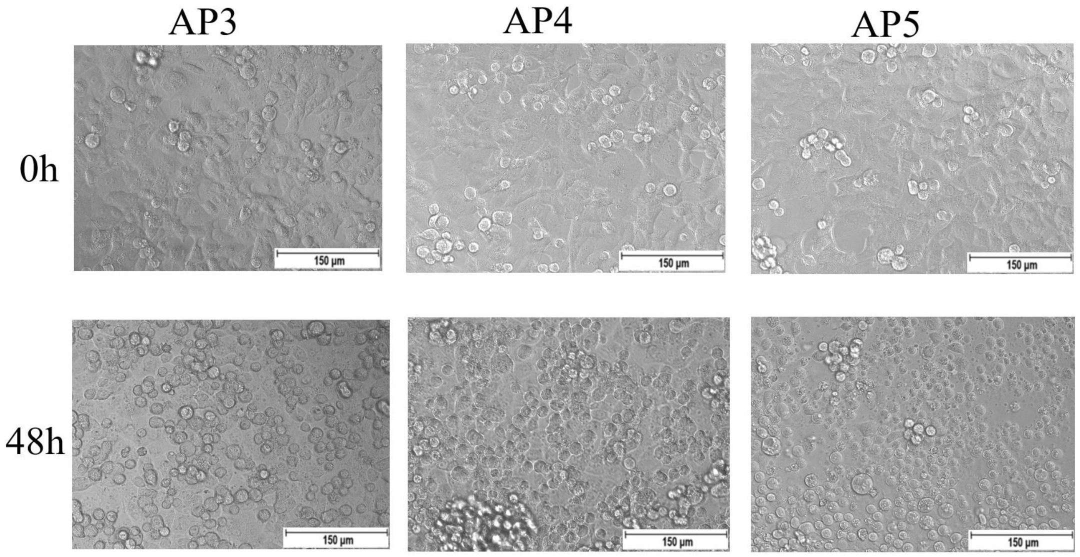

(**B**)

Figure S26. The effects of AP1, AP2 (A), AP3, AP4 and AP5 (B) on PANC-1 cells' morphology at both 0 h and 48 h after stimulation; the scale bar was 150 µm.

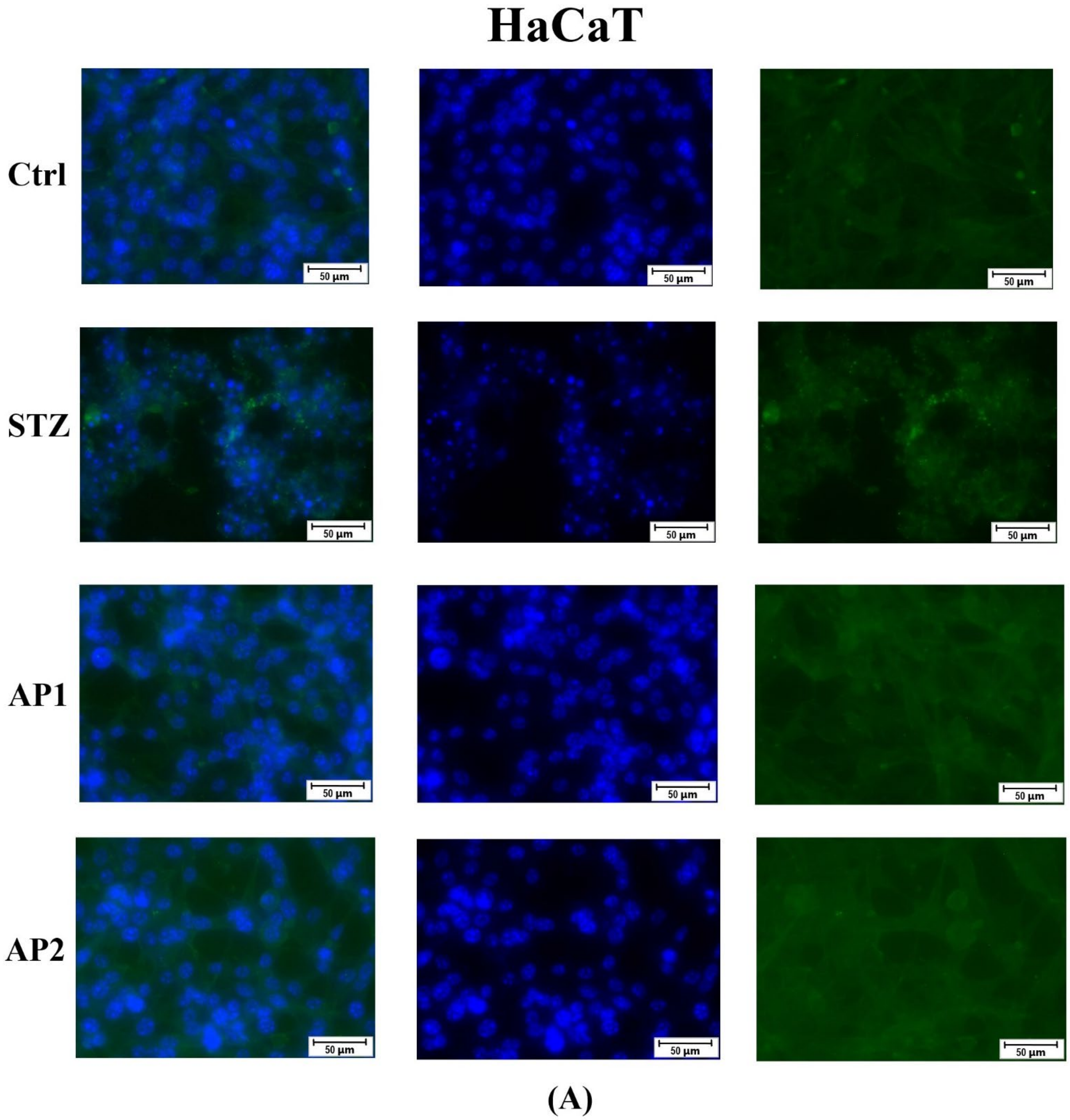

Figure S27. Enlarged Figure 3, Panel A (Ctr, STZ, AP1, AP2)

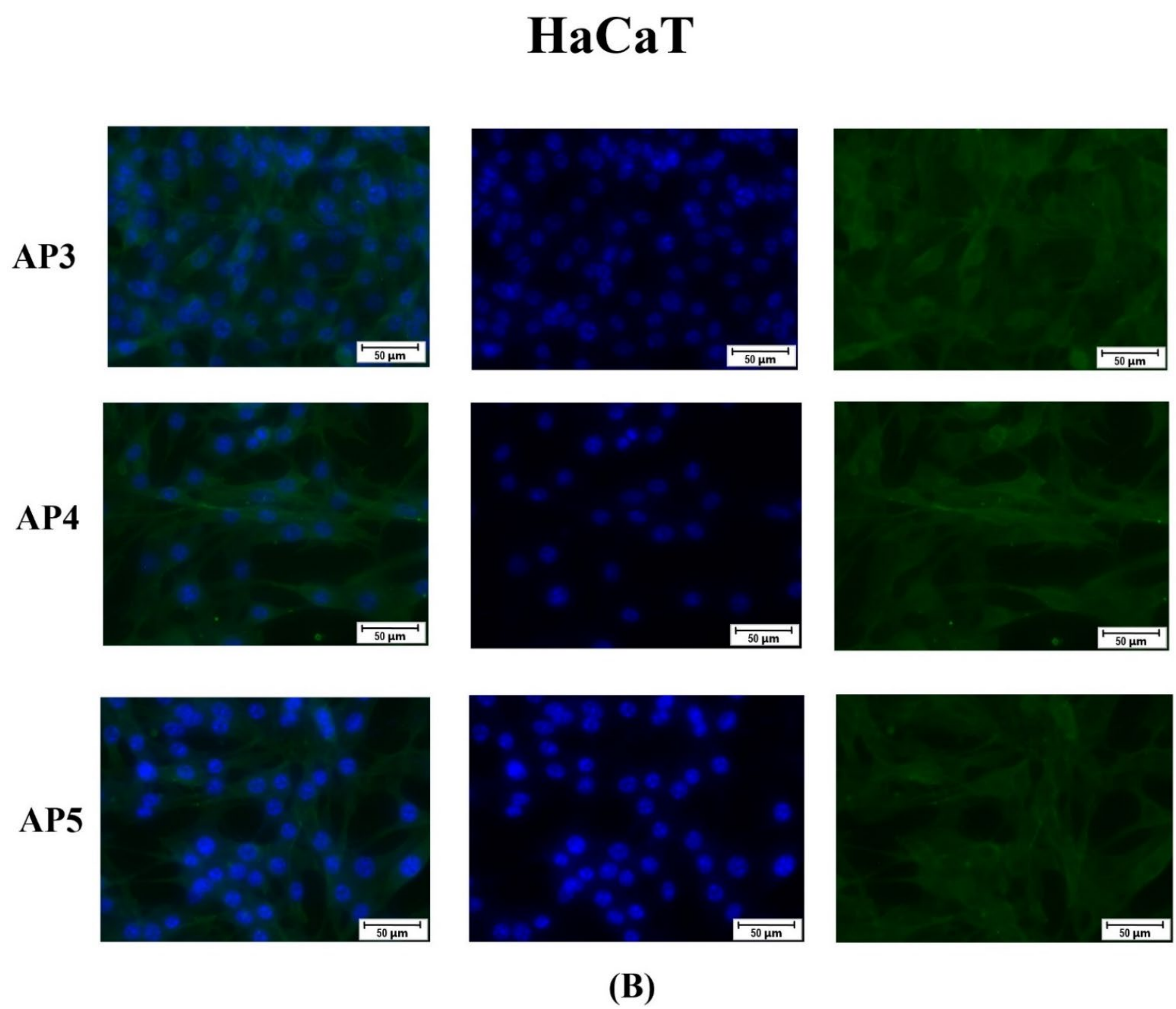

Figure S28. Enlarged Figure 3, Panel B (AP3, AP4 and AP5)

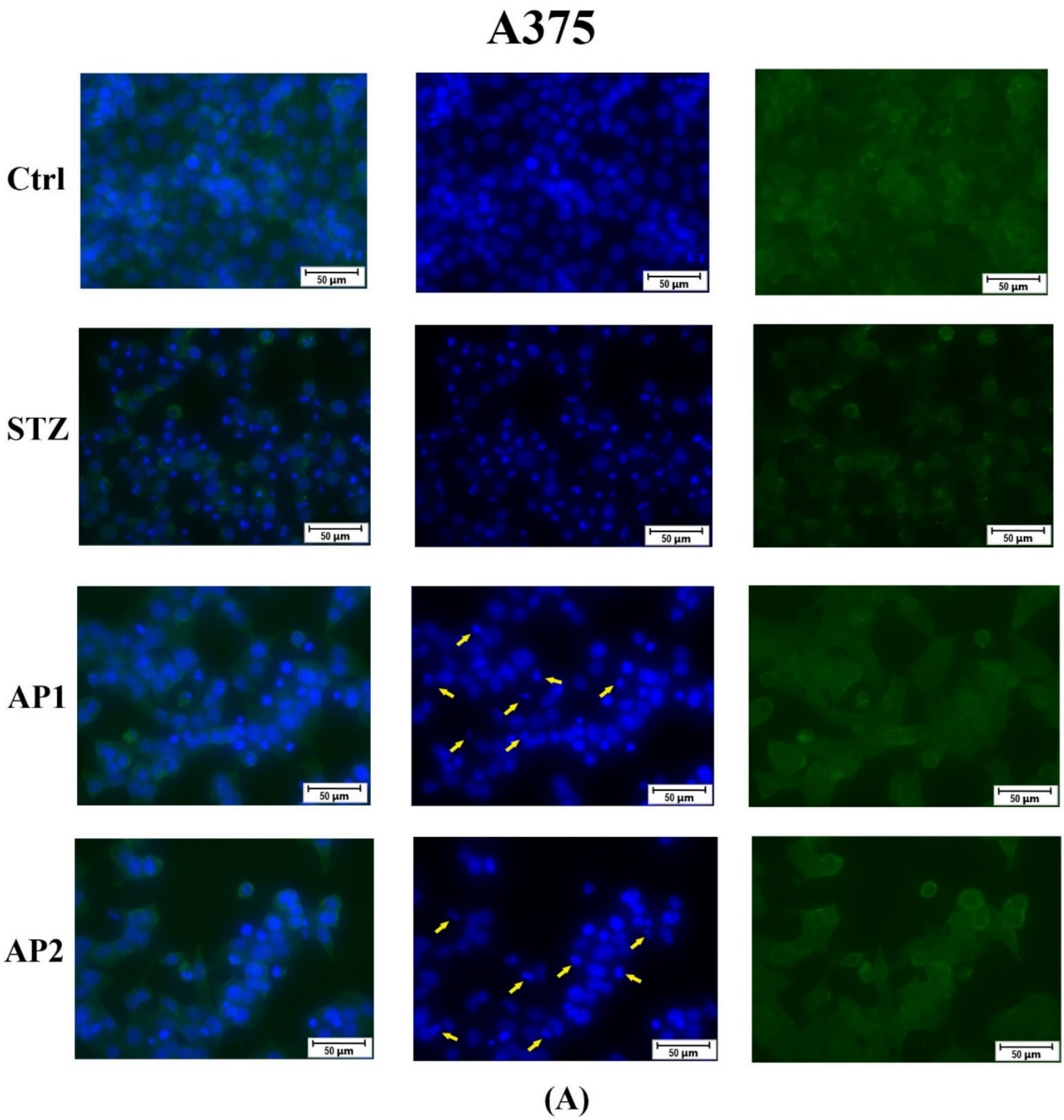

Figure S29. Enlarged Figure 4, Panel A (Ctr, STZ, AP1, AP2)

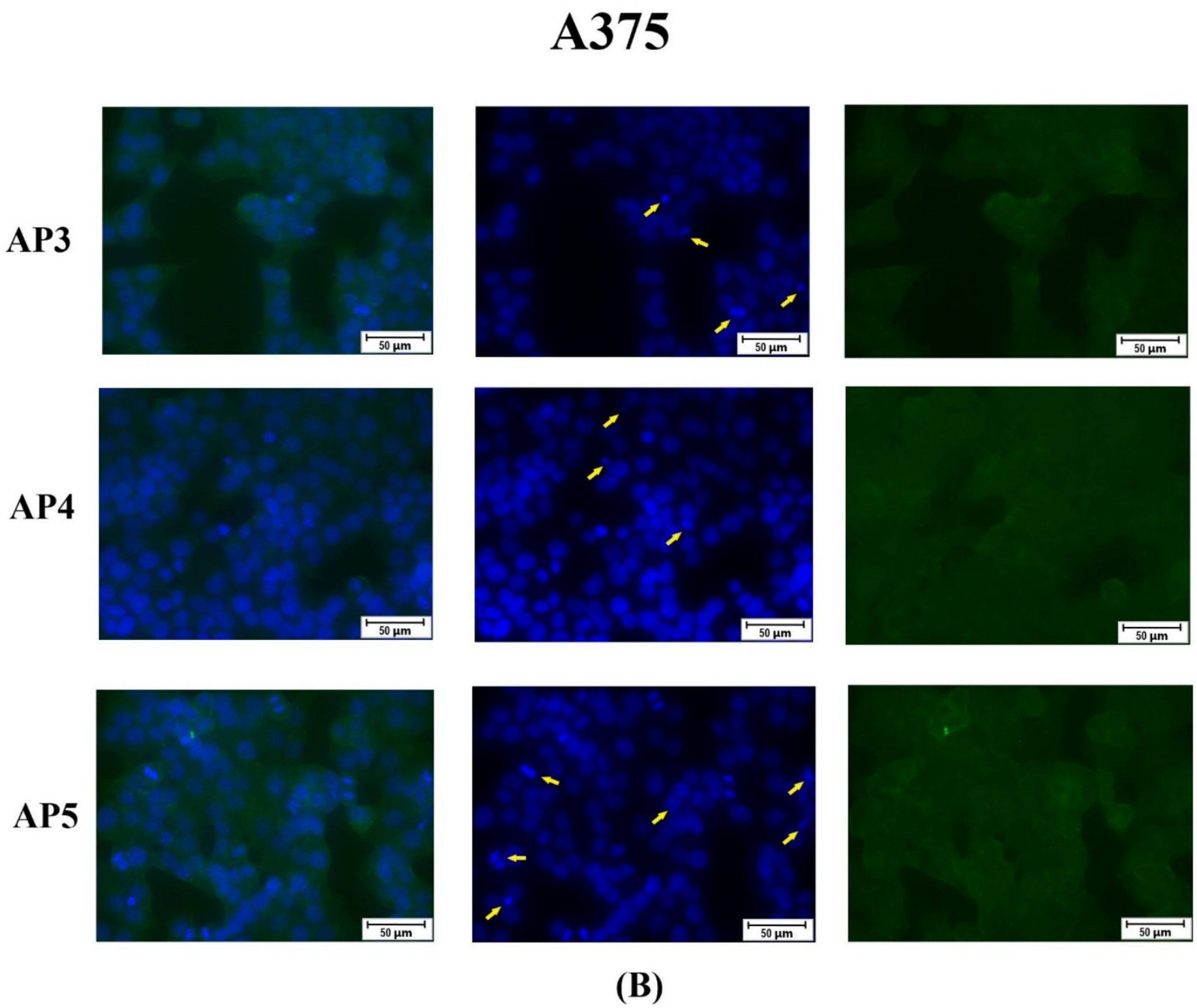

Figure S30. Enlarged Figure 4, Panel B (AP3, AP4 and AP5)

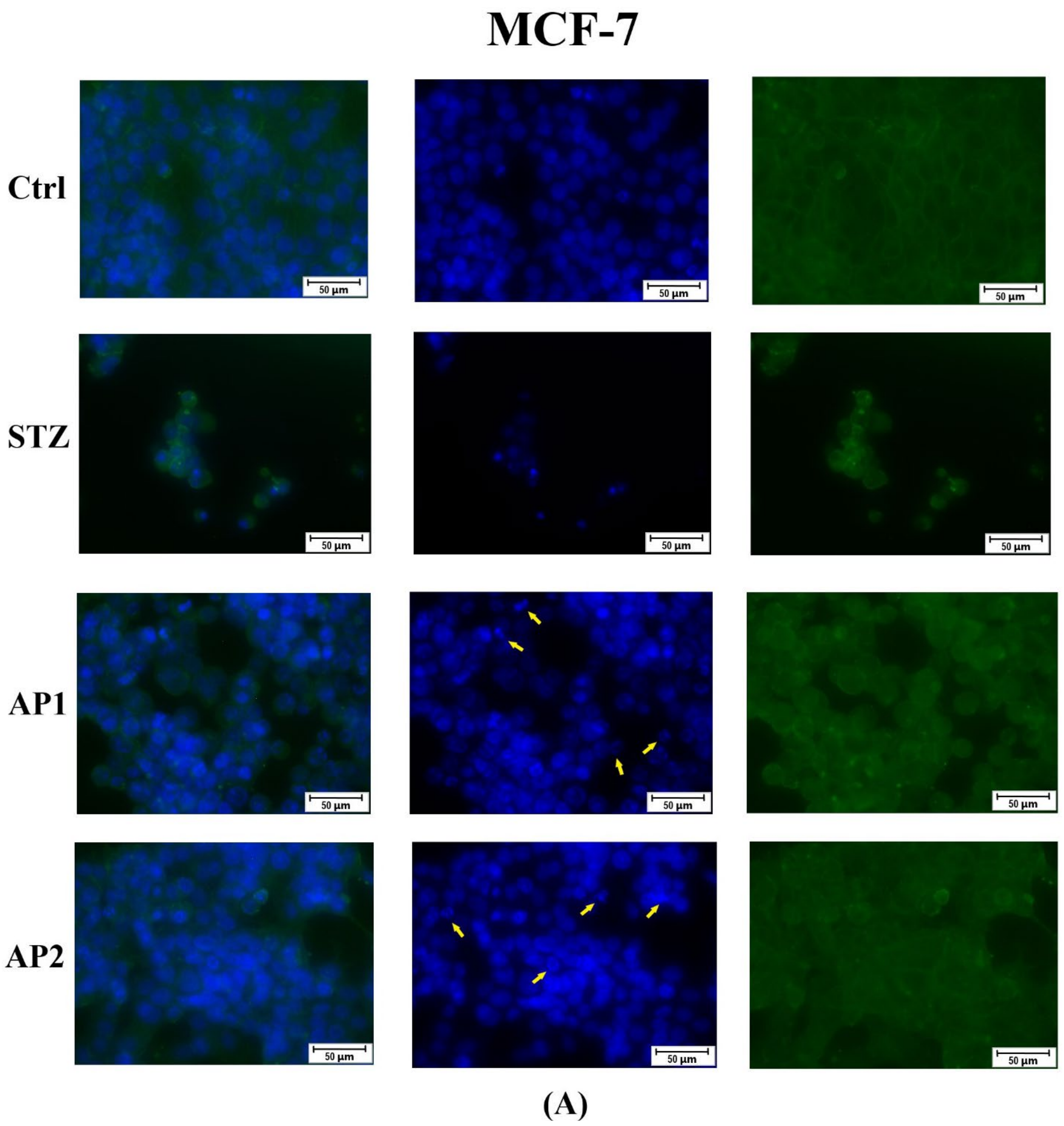

Figure S31. Enlarged Figure 5, Panel A (Ctr, STZ, AP1, AP2)

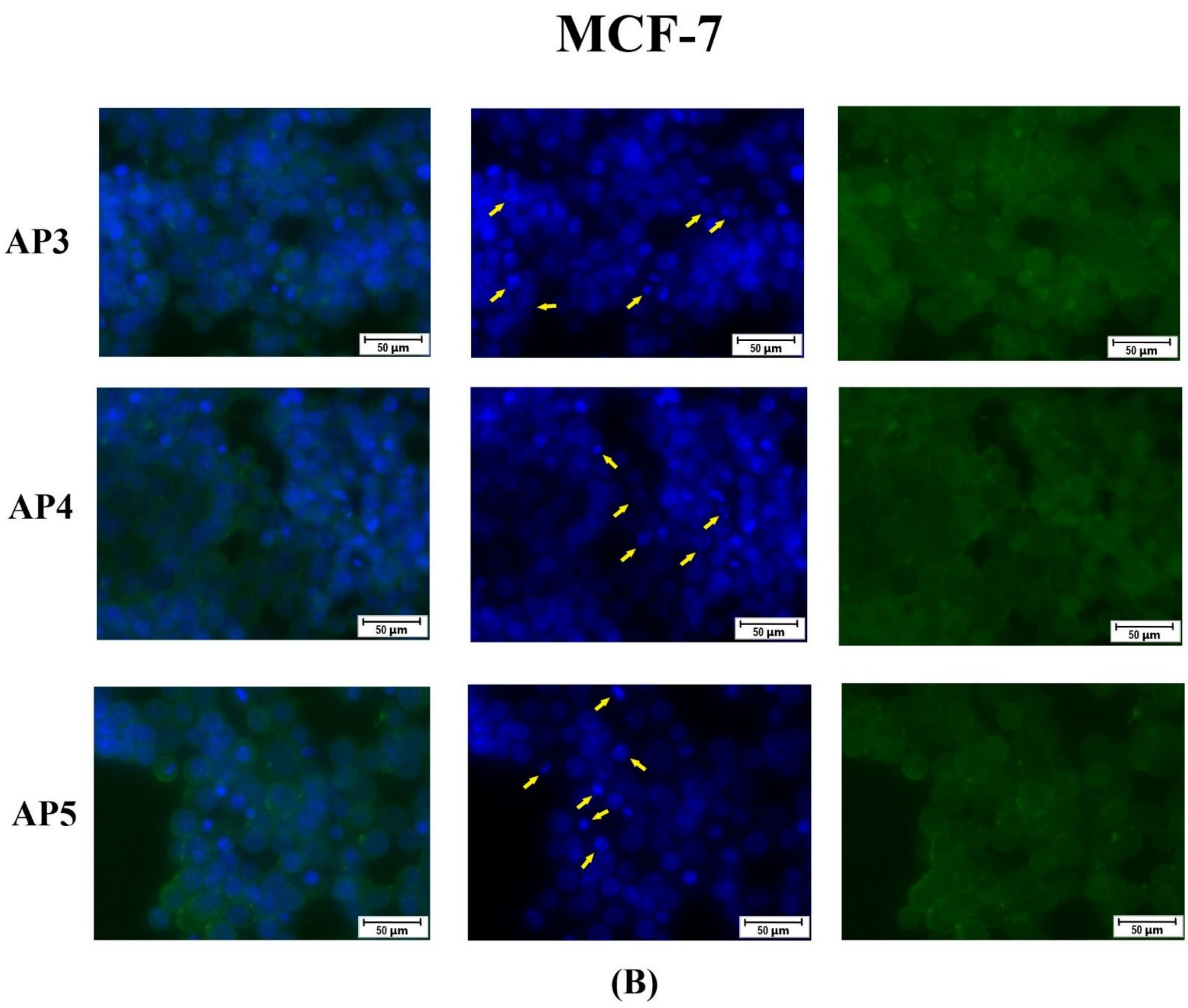

Figure S32. Enlarged Figure 5, Panel B (AP3, AP4 and AP5)

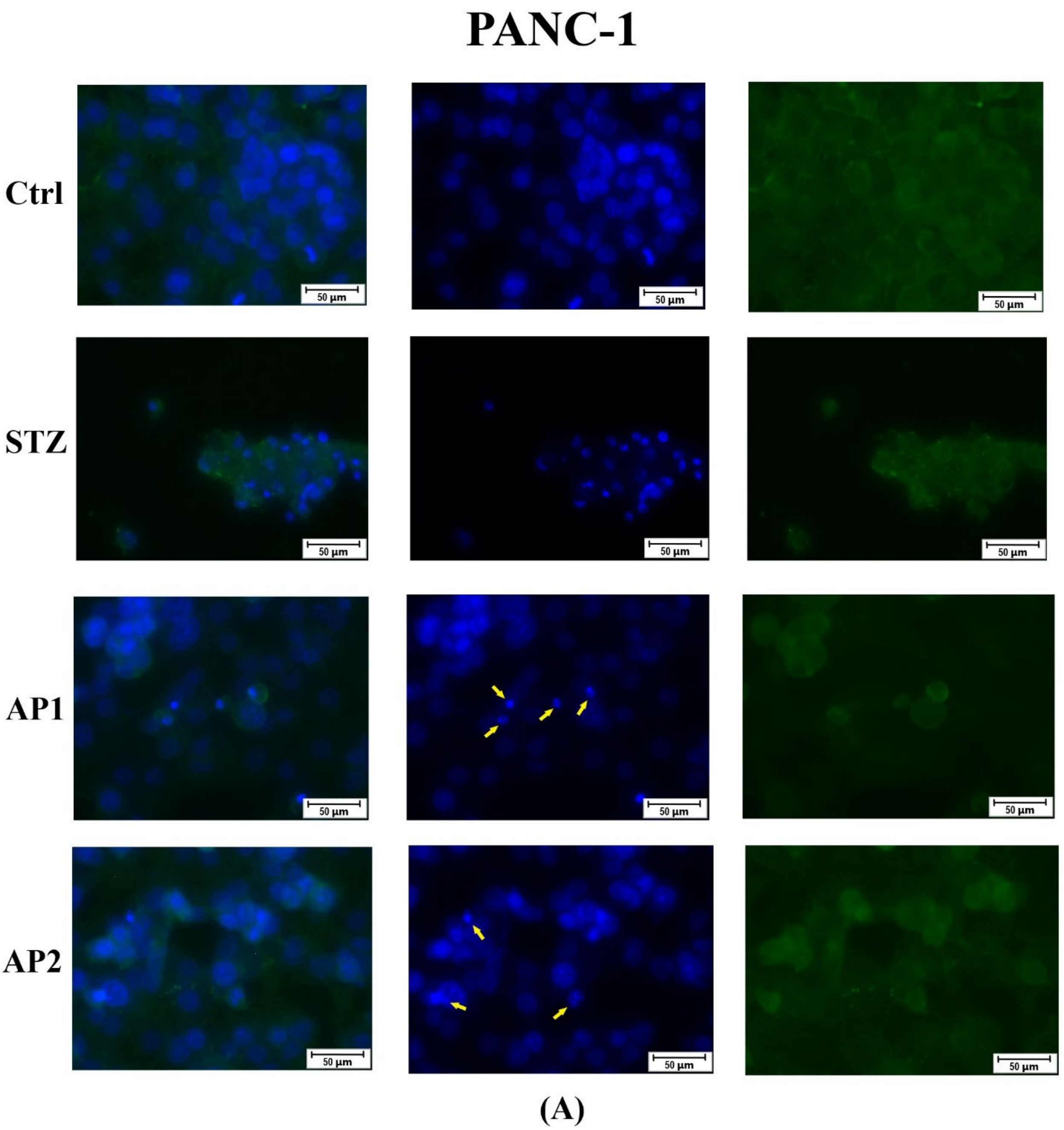

Figure S33. Enlarged Figure 6, Panel A (Ctr, STZ, AP1, AP2)

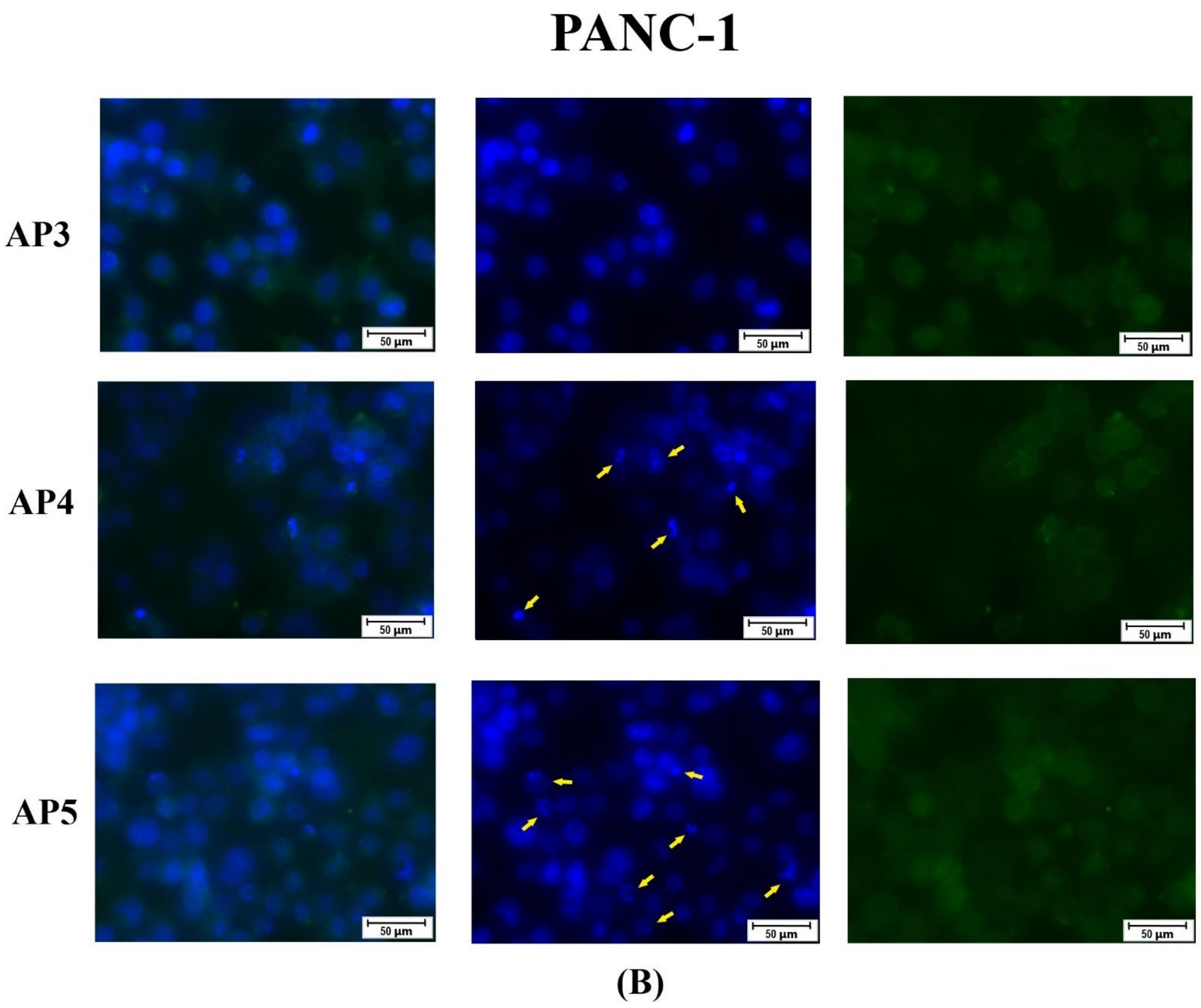

Figure S34. Enlarged Figure 6, Panel B (AP3, AP4 and AP5)

Table S1. Targets identified through the Pharmamapper platform for Bet and AP1-5

Pharma Model	Fit	Norm Fit	zscore	Uniplot	Uniprot ID	Pharma Model	Fit	Norm Fit	zscore	Uniplot	Uniprot ID
**Bet**						**AP3**					
1db1_v	5.744	0.4419	2.4695	VDR_HUMAN	P11473	1iz2_v	3.67	0.6117	1.55974	A1AT_HUMAN	P01009
1rlb_v	5.356	0.5356	1.7347	TTHY_HUMAN	P02766	1hak_v	3.694	0.9234	1.97239	ANXA5_HUMAN	P08758
2bel_v	4.906	0.4906	2.95419	DHI1_HUMAN	P28845	1ctr_v	3.741	0.7482	1.5205	CALM_HUMAN	P62158
1r5l_v	4.777	0.3981	1.4774	P49638	P49638	1csb_v	3.346	0.8366	1.40361	CATB_HUMAN	P07858
1jtv_v	4.593	0.5741	1.04125	P14061	P14061	1q6k_v	3.429	0.8573	1.46098	CATK_HUMAN	P43235
11gs_v	4.546	0.6494	2.82712	GSTP1_HUMAN	P09211	1i7b_v	3.455	0.4319	1.13554	DCAM_HUMAN	P17707
1fe3_v	4.526	0.6465	2.37172	FABP7_HUMAN	O15540	1itu_v	4.088	0.5111	1.03518	DPEP1_HUMAN	P16444
1q4x_v	4.385	0.4872	1.74429	P10828	P10828	1m17_v	3.474	0.8686	1.58572	EGFR_HUMAN	P00533
1w6k_v	4.363	0.6233	2.87477	ERG7_HUMAN	P48449	1h1b_v	3.688	0.6147	1.60854	ELNE_HUMAN	P08246
1hms_v	4.348	0.5436	1.73336	FABPH_HUMAN	P05413	1mqb_v	3.462	0.4945	1.03091	EPHA2_HUMAN	P29317
1it6_v	4.32	0.3323	1.74349	P36873	P36873	3bbt_v	3.599	0.5998	1.26754	ERBB4_HUMAN	Q15303
1rbp_v	4.295	0.5369	1.06535	RET4_HUMAN	P02753	1w6j_v	3.653	0.7307	1.72097	ERG7_HUMAN	P48449
1skx_v	4.27	0.427	1.299	NR1I2_HUMAN	O75469	1s9q_v	4.251	0.7086	2.29255	ERR3_HUMAN	P62508

1kav_v	4.183	0.5229	1.43932	PTN1_HUMAN	P18031	1qkt_v	3.469	0.8672	1.21776	ESR1_HUMAN	P03372
1ln3_v	4.144	0.3767	1.52015	Q9UKL6	Q9UKL6	1o1v_v	4.638	0.6626	2.19171	FABP6_HUMAN	P51161
1jqe_v	4.128	0.516	1.5978	P50135	P50135	1fe3_v	4.415	0.6307	1.65363	FABP7_HUMAN	O15540
1e3k_v	4.098	0.683	2.61908	PRGR_HUMAN	P06401	2hmb_v	4.407	0.6296	1.73272	FABPH_HUMAN	P05413
1gs4_v	4.092	0.5116	1.52027	ANDR_HUMAN	P10275	1nhz_v	4.282	0.5353	1.23522	GCR_HUMAN	P04150
2bk3_v	4.085	0.5835	1.18575	AOFB_HUMAN	P27338	1x0n_v	3.819	0.4244	1.09355	GRB2_HUMAN	P62993
1mv9_v	3.998	0.4998	1.54085	RXRA_HUMAN	P19793	2q6c_v	3.827	0.7655	2.55347	HMDH_HUMAN	P04035
1xap_v	3.952	0.4391	1.17973	RARB_HUMAN	P10826	1m48_v	3.946	0.5637	1.76739	IL2_HUMAN	P60568
1xha_v	3.941	0.3582	1.57106	P00517	P00517	2b7a_v	3.735	0.5336	1.07499	JAK2_HUMAN	O60674
1gnj_v	3.922	0.4902	1.0801	ALBU_HUMAN	P02768	1yvj_v	3.578	0.5963	1.07112	JAK3_HUMAN	P52333
1pmn_v	3.9	0.5572	1.51288	MK10_HUMAN	P53779	1liu_v	4.101	0.4556	1.31027	KPYR_HUMAN	P30613
1lhn_v	3.89	0.6483	1.85629	SHBG_HUMAN	P04278	2aa5_v	3.983	0.7967	1.8775	MCR_HUMAN	P08235
1fkf_v	3.869	0.4299	1.80712	FKB1A_HUMAN	P62942	1rv1_v	3.336	0.6672	1.06455	MDM2_HUMAN	Q00987
1d5j_v	3.845	0.4806	2.03564	MMP3_HUMAN	P08254	2g01_v	3.64	0.91	1.90093	MK08_HUMAN	P45983
1pq2_v	3.842	0.5488	1.80408	CP2C8_HUMAN	P10632	1utt_v	3.535	0.707	1.55756	MMP12_HUMAN	P39900
1o1v_v	3.835	0.5478	1.35313	FABP6_HUMAN	P51161	1hov_v	3.707	0.5296	1.3588	MMP2_HUMAN	P08253
1exa_v	3.835	0.3835	1.0067	RARG_HUMAN	P13631	1dmt_v	3.794	0.4742	1.86892	NEP_HUMAN	P08473
1xon_v	3.826	0.4783	1.41945	PDE4D_HUMAN	Q08499	1x89_v	3.722	0.5318	1.43229	NGAL_HUMAN	P80188
2fpy_v	3.822	0.4247	1.03346	PYRD_HUMAN	Q02127	1uhl_v	3.638	0.6064	1.26159	NR1H3_HUMAN	Q13133
2oaz_v	3.714	0.7428	2.60727	AMPM2_HUMAN	P50579	2o9i_v	3.79	0.5415	1.05565	NR1I2_HUMAN	O75469
1hrk_v	3.712	0.464	1.27798	P22830	P22830	1xvp_v	3.762	0.6271	1.49303	NR1I3_HUMAN	Q14994
1l8j_v	3.698	0.2844	1.04989	EPCR_HUMAN	Q9UNN8	1q22_v	4.3	0.6142	1.59542	O00204	O00204
1y0s_v	3.694	0.4104	1.71768	PPARD_HUMAN	Q03181	1g55_v	4.001	0.4001	1.77411	O14717	O14717
1wok_v	3.693	0.7385	2.31845	P09874	P09874	1u3w_v	3.371	0.6742	1.1959	P00326	P00326
1q22_v	3.69	0.5271	1.07334	O00204	O00204	2gqg_v	4.107	0.5134	2.05766	P00519	P00519
1nav_v	3.67	0.5243	1.29075	P10827	P10827	1gzr_v	3.604	0.6006	1.5604	P01343	P05019
1i7g_v	3.665	0.6108	2.10533	PPARA_HUMAN	Q07869	1n69_v	3.677	0.5253	1.33267	P07602	P07602
2ofu_v	3.662	0.4577	1.42067	LCK_HUMAN	P06239	1gse_v	3.743	0.6238	1.22229	P08263	P08263
1x0n_v	3.652	0.4058	1.30291	GRB2_HUMAN	P62993	1nav_v	4.373	0.6246	2.11795	P10827	P10827
2zas_v	3.648	0.912	2.39864	P62508	P62508	3fzk_v	3.389	0.8472	1.52222	P11142	P11142
1t64_v	3.645	0.405	1.1206	HDAC8_HUMAN	Q9BY41	1qyw_v	3.794	0.7587	1.75147	P14061	P14061
1t4e_v	3.63	0.6051	1.4352	MDM2_HUMAN	Q00987	1dcy_v	3.648	0.7297	1.57153	P14555	P14555
1irj_v	3.614	0.5163	1.94245	S10A9_HUMAN	P06702	1r7t_v	3.658	0.6096	1.78155	P16442	P16442
1pic_v	3.614	0.4015	1.92555	P85A_HUMAN	P27986	1hrk_v	3.879	0.4848	1.10142	P22830	P22830
1qvn_v	3.609	0.4511	1.43668	IL2_HUMAN	P60568	3ey4_v	3.439	0.6878	1.2047	P28845	P28845
1t84_v	3.587	0.7174	1.66861	WASP_HUMAN	P42768	1cbs_v	6.191	0.6191	1.42591	P29373	P29373
2vd1_v	3.573	0.5955	1.25412	PTGD2_HUMAN	P41222	1he3_v	3.653	0.6089	1.47987	P30043	P30043
1j78_v	3.57	0.8924	1.66753	VTDB_HUMAN	P02774	2i6b_v	3.998	0.6663	1.5605	P55263	P55263
2g24_v	3.559	0.5932	2.23143	RENI_HUMAN	P00797	2zdz_v	3.786	0.6309	1.714	P56817	P56817
1u3w_v	3.558	0.7117	2.42951	P00326	P00326	1dmw_v	3.57	0.7141	1.60589	PH4H_HUMAN	P00439
1rdt_v	3.558	0.5083	1.48439	PPARG_HUMAN	P37231	1i7g_v	3.784	0.6306	1.73714	PPARA_HUMAN	Q07869
2fme_v	3.557	0.7114	1.80038	KIF11_HUMAN	P52732	1nwe_v	3.746	0.7491	1.6669	PTN1_HUMAN	P18031
1dkf_v	3.552	0.444	1.00005	RARA_HUMAN	P10276	1j99_v	3.572	0.8929	1.76728	Q06520	Q06520
1r78_v	3.529	0.5042	1.41778	P24941	P24941	1sm2_v	3.839	0.6398	1.5315	Q08881	Q08881
1gsf_v	3.519	0.4398	1.0188	P08263	P08263	1lv2_v	4.59	0.6557	2.40148	Q14541	Q14541

2i16_v	3.501	0.5835	1.23195	ALDR_HUMAN	P15121	1x9d_v	3.363	0.4804	1.03036	Q9UKM7	Q9UKM7
1r7u_v	3.49	0.3878	1.0538	P16442	P16442	1dkf_v	4.312	0.539	1.88368	RARA_HUMAN	P10276
1bl6_v	3.481	0.8701	2.06017	Q16539	Q16539	1xap_v	4.471	0.4968	1.50311	RARB_HUMAN	P10826
1sz7_v	3.478	0.5797	1.16427	O43617	O43617	1fd0_v	4.418	0.5522	1.90292	RARG_HUMAN	P13631
2irz_v	3.468	0.4336	1.57764	BACE1_HUMAN	P56817	1h9u_v	4.694	0.4694	1.36177	RXRB_HUMAN	P28702
2f4j_v	3.467	0.4334	1.26945	P00519	P00519	1irj_v	3.792	0.5417	1.72663	S10A9_HUMAN	P06702
1uhl_v	3.465	0.5774	1.4583	NR1H3_HUMAN	Q13133	1l9n_v	4.319	0.5399	1.36315	TGM3_HUMAN	Q08188
2e9v_v	3.464	0.4949	1.08672	CHK1_HUMAN	O14757	1s0z_v	4.596	0.4596	1.74556	VDR_HUMAN	P11473
1rd4_v	3.425	0.5708	1.2519	ITAL_HUMAN	P20701	1j78_v	3.616	0.9039	1.12051	VTDB_HUMAN	P02774
3dct_v	3.421	0.3801	1.12112	Q96RI1	Q96RI1	1t84_v	3.764	0.7527	1.46254	WASP_HUMAN	P42768
2q6b_v	3.379	0.4224	1.28382	HMDH_HUMAN	P04035	**AP4**					
1xws_v	3.328	0.4755	1.47399	PIM1_HUMAN	P11309	1iz2_v	3.387	0.5646	1.03871	A1AT_HUMAN	P01009
1xvp_v	3.303	0.5505	1.01542	NR1I3_HUMAN	Q14994	1ma0_v	3.802	0.6336	1.09227	ADHX_HUMAN	P11766
1g2l_v	3.224	0.5373	1.41742	FA10_HUMAN	P00742	1hak_v	3.576	0.894	1.75112	ANXA5_HUMAN	P08758
3f7z_v	3.216	0.6433	1.3373	P49841	P49841	1i7b_v	3.732	0.4664	2.04579	DCAM_HUMAN	P17707
1p49_v	3	1	1.08335	STS_HUMAN	P08842	1pl6_v	3.554	0.7108	1.19193	DHSO_HUMAN	Q00796
**AP1**						1m17_v	3.702	0.9256	2.17505	EGFR_HUMAN	P00533
1iz2_v	3.576	0.5959	1.30822	A1AT_HUMAN	P01009	1w6j_v	3.756	0.7512	2.05371	ERG7_HUMAN	P48449
1egc_v	3.341	0.4773	1.19359	ACADM_HUMAN	P11310	1s9p_v	4.441	0.7402	2.84799	ERR3_HUMAN	P62508
1ma0_v	3.877	0.6462	1.10873	ADHX_HUMAN	P11766	1qkt_v	3.619	0.9047	1.68241	ESR1_HUMAN	P03372
1hak_v	3.443	0.8607	1.18449	ANXA5_HUMAN	P08758	1ya4_v	3.362	0.8405	1.34028	EST1_HUMAN	P23141
2iiv_v	3.619	0.7238	1.56316	DPP4_HUMAN	P27487	1zom_v	3.62	0.4525	2.01109	FA11_HUMAN	P03951
2ity_v	3.385	0.8462	1.33528	EGFR_HUMAN	P00533	1o1v_v	4.168	0.5954	1.50945	FABP6_HUMAN	P51161
1h1b_v	3.757	0.6262	1.71275	ELNE_HUMAN	P08246	1fe3_v	4.236	0.6051	1.4406	FABP7_HUMAN	O15540
1l8j_v	4.895	0.3765	2.56862	EPCR_HUMAN	Q9UNN8	2hmb_v	4.32	0.6171	1.6679	FABPH_HUMAN	P05413
1mqb_v	3.496	0.4995	1.05526	EPHA2_HUMAN	P29317	1fgi_v	3.25	0.6501	1.16466	FGFR1_HUMAN	P11362
1w6j_v	3.653	0.7306	1.66664	ERG7_HUMAN	P48449	1nhz_v	4.762	0.5952	2.00486	GCR_HUMAN	P04150
1g50_v	3.33	0.8325	1.06277	ESR1_HUMAN	P03372	1xwk_v	3.516	0.3907	1.16708	GSTM1_HUMAN	P09488
2j95_v	3.38	0.845	1.21038	FA10_HUMAN	P00742	1qcf_v	3.766	0.538	1.05203	HCK_HUMAN	P08631
1fe3_v	4.439	0.6341	1.65735	FABP7_HUMAN	O15540	1m48_v	3.614	0.5163	1.18605	IL2_HUMAN	P60568
2hmb_v	4.12	0.5886	1.20532	FABPH_HUMAN	P05413	2b7a_v	3.96	0.5657	1.60342	JAK2_HUMAN	O60674
1fgi_v	3.474	0.6947	1.48816	FGFR1_HUMAN	P11362	1yvj_v	3.583	0.5971	1.1752	JAK3_HUMAN	P52333
1nhz_v	4.923	0.6154	2.12985	GCR_HUMAN	P04150	1ya3_v	3.615	0.9036	1.8868	MCR_HUMAN	P08235
1x0n_v	3.868	0.4298	1.14548	GRB2_HUMAN	P62993	1utt_v	3.325	0.665	1.12376	MMP12_HUMAN	P39900
1qcf_v	4.172	0.596	1.6761	HCK_HUMAN	P08631	1hov_v	3.763	0.5376	1.54634	MMP2_HUMAN	P08253
2q6c_v	3.289	0.6578	1.05065	HMDH_HUMAN	P04035	1sg0_v	3.261	0.6522	1.18096	NQO2_HUMAN	P16083
1m48_v	3.735	0.5335	1.29862	IL2_HUMAN	P60568	1ilh_v	3.69	0.4612	1.01248	NR1I2_HUMAN	O75469
1yvj_v	3.637	0.6061	1.15113	JAK3_HUMAN	P52333	1xvp_v	4.346	0.7243	2.92059	NR1I3_HUMAN	Q14994
1liu_v	4.013	0.4459	1.12633	KPYR_HUMAN	P30613	1q22_v	4.618	0.6597	2.16992	O00204	O00204
1xbc_v	3.567	0.7134	1.47113	KSYK_HUMAN	P43405	1ydt_v	3.517	0.7035	1.46529	P00517	P00517
1qpe_v	4.457	0.7429	2.75386	LCK_HUMAN	P06239	2hzi_v	3.739	0.4673	1.21216	P00519	P00519
1ya3_v	3.412	0.8529	1.31995	MCR_HUMAN	P08235	1gzr_v	3.373	0.5621	1.07644	P01343	P05019
1r0p_v	3.67	0.6117	1.03888	MET_HUMAN	P08581	1gse_v	3.911	0.6518	1.66311	P08263	P08263
1hov_v	3.632	0.5189	1.17111	MMP2_HUMAN	P08253	1nav_v	4.524	0.6462	2.50621	P10827	P10827
1d8m_v	3.561	0.5935	1.31578	MMP3_HUMAN	P08254	1qyx_v	3.864	0.7728	1.42051	P14061	P14061

1s9j_v	5.044	0.5605	1.87012	MP2K1_HUMAN	Q02750	1dcy_v	3.519	0.7039	1.3368	P14555	P14555
1skx_v	5.105	0.5105	1.93791	NR1I2_HUMAN	O75469	1hrk_v	4.195	0.5243	1.6957	P22830	P22830
1xvp_v	4.232	0.7054	2.50241	NR1I3_HUMAN	Q14994	1he2_v	3.401	0.5668	1.09195	P30043	P30043
1q22_v	5.223	0.7461	2.99708	O00204	O00204	1u32_v	4.482	0.4075	1.65899	P36873	P36873
1u3w_v	3.429	0.6858	1.28775	P00326	P00326	1oiz_v	3.561	0.7122	1.33696	P49638	P49638
1ydt_v	3.669	0.7339	1.68277	P00517	P00517	3f7z_v	3.298	0.6596	1.04454	P49841	P49841
2hzi_v	3.785	0.4731	1.17538	P00519	P00519	2i6b_v	3.862	0.6437	1.37395	P55263	P55263
1gzr_v	3.627	0.6046	1.55824	P01343	P05019	1pic_v	3.649	0.4054	1.46543	P85A_HUMAN	P27986
1p9a_v	3.292	0.4115	1.03918	P07359	P07359	1so2_v	3.412	0.6825	1.4685	PDE3B_HUMAN	Q13370
1gse_v	4.008	0.6679	1.72485	P08263	P08263	1dmw_v	3.549	0.7099	1.65918	PH4H_HUMAN	P00439
1nav_v	4.364	0.6235	2.05862	P10827	P10827	1i7g_v	3.958	0.6597	2.26461	PPARA_HUMAN	Q07869
1qyw_v	3.844	0.7688	1.81052	P14061	P14061	1onz_v	3.693	0.7385	2.06874	PTN1_HUMAN	P18031
1r7t_v	3.42	0.5701	1.10216	P16442	P16442	1j99_v	3.444	0.861	1.55804	Q06520	Q06520
1tjj_v	3.646	0.6077	1.72557	P17900	P17900	2yxj_v	3.448	0.3831	1.1246	Q07817	Q07817
1hrk_v	3.892	0.4865	1.08481	P22830	P22830	1sm2_v	3.905	0.6509	1.77968	Q08881	Q08881
3ey4_v	3.404	0.6808	1.05312	P28845	P28845	1dkf_v	4.095	0.5118	1.57978	RARA_HUMAN	P10276
3cbs_v	4.936	0.5485	1.6217	P29373	P29373	1h9u_v	4.573	0.4573	1.27703	RXRB_HUMAN	P28702
1he3_v	3.733	0.6222	1.62124	P30043	P30043	1irj_v	3.501	0.5002	1.12662	S10A9_HUMAN	P06702
1oiz_v	3.594	0.7188	1.26737	P49638	P49638	1lhw_v	3.703	0.9258	2.25943	SHBG_HUMAN	P04278
2i6b_v	4.552	0.7587	2.63883	P55263	P55263	2h8h_v	3.196	0.7991	1.05598	SRC_HUMAN	P12931
1pic_v	3.703	0.4115	1.4387	P85A_HUMAN	P27986	1vjy_v	3.568	0.892	2.03912	TGFR1_HUMAN	P36897
1so2_v	3.416	0.6832	1.31448	PDE3B_HUMAN	Q13370	1l9n_v	4.984	0.623	2.47974	TGM3_HUMAN	Q08188
1i7g_v	3.999	0.6665	2.20004	PPARA_HUMAN	Q07869	1s19_v	3.55	0.4437	1.02901	VDR_HUMAN	P11473
1mrq_v	3.748	0.7496	1.77347	Q04828	Q04828	1j78_v	3.733	0.9331	1.5181	VTDB_HUMAN	P02774
2yxj_v	3.968	0.4409	2.24103	Q07817	Q07817	**AP5**					
1sm2_v	4.013	0.6689	1.87152	Q08881	Q08881	1iz2_v	3.598	0.5997	1.62455	A1AT_HUMAN	P01009
2i3i_v	3.823	0.4779	1.73932	Q96CA5	Q96CA5	1egc_v	3.364	0.4805	1.55257	ACADM_HUMAN	P11310
1m6d_v	3.584	0.512	1.34884	Q9UBX1	Q9UBX1	1ma0_v	3.892	0.6486	1.40157	ADHX_HUMAN	P11766
1ln3_v	4.284	0.3895	1.23538	Q9UKL6	Q9UKL6	1gni_v	3.865	0.5521	1.0041	ALBU_HUMAN	P02768
1xap_v	4.478	0.4975	1.48237	RARB_HUMAN	P10826	1of7_v	3.114	0.6228	1.03089	ALDH2_HUMAN	P05091
1fcz_v	4	0.5714	1.70764	RARG_HUMAN	P13631	1boa_v	4.137	0.3761	1.24055	AMPM2_HUMAN	P50579
1lho_v	4.652	0.7753	2.0418	SHBG_HUMAN	P04278	2bk3_v	4.226	0.6037	1.14591	AOFB_HUMAN	P27338
1l9n_v	4.387	0.5484	1.4346	TGM3_HUMAN	Q08188	2f3e_v	4.215	0.2634	1.07082	BACE1_HUMAN	P56817
1s19_v	3.69	0.4613	1.18179	VDR_HUMAN	P11473	1g54_v	4.422	0.6317	1.83939	CAH2_HUMAN	P00918
1j78_v	3.689	0.9222	1.24348	VTDB_HUMAN	P02774	1bmq_v	3.722	0.3384	1.06308	CASP1_HUMAN	P29466
1u59_v	3.705	0.5293	1.09161	ZAP70_HUMAN	P43403	1gmy_v	3.801	0.4224	1.32078	CATB_HUMAN	P07858
**AP2**						1bgo_v	3.946	0.4384	1.59584	CATK_HUMAN	P43235
1iz2_v	3.621	0.6034	1.59297	A1AT_HUMAN	P01009	2iw6_v	3.779	0.5399	1.46942	CCNA2_HUMAN	P20248
1ma0_v	3.732	0.622	1.00953	ADHX_HUMAN	P11766	2e9v_v	3.878	0.554	1.59164	CHK1_HUMAN	O14757
1of7_v	3.149	0.6299	1.02036	ALDH2_HUMAN	P05091	1t31_v	3.502	0.4378	1.04823	CMA1_HUMAN	P23946
2ea2_v	3.506	0.5843	1.09931	AMPM2_HUMAN	P50579	2bel_v	4.95	0.495	2.7179	DHI1_HUMAN	P28845
2fb8_v	3.644	0.9111	2.21447	BRAF1_HUMAN	P15056	1pl6_v	3.515	0.703	1.23542	DHSO_HUMAN	Q00796
1isi_v	3.439	0.5732	1.80109	BST1_HUMAN	Q10588	1hne_v	3.935	0.4372	1.11804	ELNE_HUMAN	P08246
1u4l_v	3.276	0.546	1.20657	CCL5_HUMAN	P13501	1l8j_v	3.917	0.3013	1.15276	EPCR_HUMAN	Q9UNN8
2oqi_v	3.661	0.7322	1.88876	DPP4_HUMAN	P27487	2vwv_v	3.853	0.7706	2.50891	EPHB4_HUMAN	P54760

1q5h_v	4.142	0.4142	1.24481	DUT_HUMAN	P33316	1w6j_v	3.845	0.7689	2.41778	ERG7_HUMAN	P48449
1h1b_v	3.52	0.5866	1.36058	ELNE_HUMAN	P08246	1s9p_v	4.086	0.6811	2.30034	ERR3_HUMAN	P62508
1mqb_v	3.537	0.5053	1.36812	EPHA2_HUMAN	P29317	1xp9_v	3.931	0.5616	1.98811	ESR1_HUMAN	P03372
1w6j_v	3.804	0.7608	2.23329	ERG7_HUMAN	P48449	1nde_v	3.713	0.4125	1.05768	ESR2_HUMAN	Q92731
1s9p_v	3.83	0.6384	1.70773	ERR3_HUMAN	P62508	2uwo_v	3.564	0.594	1.9987	FA10_HUMAN	P00742
1o1v_v	3.856	0.5509	1.05193	FABP6_HUMAN	P51161	1zpb_v	3.422	0.4277	1.08733	FA11_HUMAN	P03951
1fe3_v	4.226	0.6037	1.49813	FABP7_HUMAN	O15540	1o1v_v	3.817	0.5453	1.04723	FABP6_HUMAN	P51161
2hmb_v	4.368	0.624	1.83691	FABPH_HUMAN	P05413	1fe3_v	4.242	0.6061	1.58407	FABP7_HUMAN	O15540
1fgi_v	3.5	0.7001	1.70508	FGFR1_HUMAN	P11362	1hms_v	4.141	0.5177	1.1409	FABPH_HUMAN	P05413
1nhz_v	4.715	0.5894	2.02764	GCR_HUMAN	P04150	1fki_v	4.567	0.5709	2.34986	FKB1A_HUMAN	P62942
1x0n_v	3.877	0.4308	1.36505	GRB2_HUMAN	P62993	1pbk_v	3.521	0.3201	1.39949	FKBP3_HUMAN	Q00688
1uy8_v	4.276	0.6109	2.40978	HS90A_HUMAN	P07900	1sa4_v	3.574	0.5106	1.19305	FNTA_HUMAN	P49354
1m48_v	3.958	0.5654	1.95251	IL2_HUMAN	P60568	1nhz_v	5.194	0.6492	2.79702	GCR_HUMAN	P04150
2b7a_v	3.915	0.5593	1.58118	JAK2_HUMAN	O60674	1x0n_v	3.718	0.4131	1.10682	GRB2_HUMAN	P62993
1yvj_v	3.587	0.5978	1.23485	JAK3_HUMAN	P52333	1xwk_v	3.659	0.4066	1.60899	GSTM1_HUMAN	P09488
1liu_v	3.961	0.4401	1.21199	KPYR_HUMAN	P30613	1t64_v	3.785	0.4205	1.08396	HDAC8_HUMAN	Q9BY41
1ya3_v	3.538	0.8845	1.76548	MCR_HUMAN	P08235	1hw9_v	4.15	0.5187	2.3439	HMDH_HUMAN	P04035
1rv1_v	3.611	0.7222	1.97452	MDM2_HUMAN	Q00987	1osf_v	4.288	0.3573	1.38623	HS90A_HUMAN	P07900
1r0p_v	3.575	0.5958	1.01994	MET_HUMAN	P08581	1qvn_v	3.825	0.4782	1.56363	IL2_HUMAN	P60568
2hu6_v	3.304	0.6609	1.31109	MMP12_HUMAN	P39900	1yvj_v	3.543	0.5905	1.21906	JAK3_HUMAN	P52333
1hov_v	3.951	0.5644	1.97463	MMP2_HUMAN	P08253	1liu_v	4.084	0.4538	1.48687	KPYR_HUMAN	P30613
1s9j_v	4.495	0.4995	1.33419	MP2K1_HUMAN	Q02750	1xbb_v	3.558	0.593	1.03365	KSYK_HUMAN	P43405
1dxo_v	3.548	0.5914	1.2476	NQO1_HUMAN	P15559	1qpd_v	3.624	0.6041	1.24303	LCK_HUMAN	P06239
1q22_v	4.13	0.59	1.46946	O00204	O00204	2aa5_v	3.512	0.7025	1.16203	MCR_HUMAN	P08235
1o6u_v	3.604	0.7207	1.03789	O76054	O76054	1t4e_v	3.878	0.6464	1.62808	MDM2_HUMAN	Q00987
1u3w_v	3.551	0.7102	1.85505	P00326	P00326	1utz_v	3.988	0.5697	2.21222	MMP12_HUMAN	P39900
1gzr_v	3.656	0.6093	1.85757	P01343	P05019	1hov_v	3.627	0.5182	1.40374	MMP2_HUMAN	P08253
1uym_v	3.576	0.447	1.38486	P08238	P08238	1ciz_v	4.234	0.4704	1.44656	MMP3_HUMAN	P08254
1gse_v	3.872	0.6454	1.64887	P08263	P08263	1mmb_v	3.524	0.4405	1.03801	MMP8_HUMAN	P22894
1nav_v	4.623	0.6605	2.81354	P10827	P10827	1s9j_v	4.45	0.4945	1.31901	MP2K1_HUMAN	Q02750
1qyx_v	3.794	0.7589	1.34252	P14061	P14061	1pq6_v	3.877	0.3877	1.21971	NR1H2_HUMAN	P55055
1hrk_v	4.115	0.5144	1.63121	P22830	P22830	1q22_v	4.18	0.5972	1.60714	O00204	O00204
2clx_v	3.185	0.7961	1.10818	P24941	P24941	2w1g_v	3.287	0.6574	1.27567	O14965	O14965
3ey4_v	3.525	0.705	1.57701	P28845	P28845	1kt8_v	3.838	0.3489	1.02358	O15382	O15382
3cbs_v	4.769	0.5299	1.58799	P29373	P29373	1o6u_v	3.648	0.7297	1.20531	O76054	O76054
1he3_v	3.484	0.5806	1.20194	P30043	P30043	1u3w_v	3.384	0.6768	1.48594	P00326	P00326
1u32_v	4.882	0.4438	2.35991	P36873	P36873	1xh5_v	3.811	0.4234	1.1652	P00517	P00517
1oiz_v	3.812	0.7624	1.96568	P49638	P49638	1gzr_v	3.505	0.5842	1.55886	P01343	P05019
2i6b_v	4.39	0.7317	2.55908	P55263	P55263	1uym_v	3.538	0.4423	1.38345	P08238	P08238
1so2_v	3.459	0.6918	1.6375	PDE3B_HUMAN	Q13370	1gsf_v	4.669	0.5836	2.76728	P08263	P08263
1a28_v	3.835	0.9587	2.50749	PRGR_HUMAN	P06401	1nav_v	4.159	0.5942	1.95024	P10827	P10827
2vd1_v	4.629	0.7716	2.89444	PTGD2_HUMAN	P41222	1q4x_v	4.318	0.4798	1.37951	P10828	P10828
1ov4_v	4.412	0.8824	2.51105	Q06520	Q06520	3fzk_v	3.113	0.7783	1.09415	P11142	P11142
2yxj_v	3.512	0.3902	1.32982	Q07817	Q07817	1jtv_v	4.88	0.6101	1.14153	P14061	P14061
1sm2_v	3.565	0.5942	1.05446	Q08881	Q08881	1kqu_v	4.269	0.5336	1.93403	P14555	P14555

1lv2_v	4.087	0.5839	1.6445	Q14541	Q14541	1wt2_v	3.797	0.3797	1.2171	P16442	P16442
1bl6_v	3.437	0.8593	1.44143	Q16539	Q16539	1zdz_v	3.452	0.4931	1.28982	P19623	P19623
1lt8_v	3.545	0.4432	1.04906	Q93088	Q93088	1gz8_v	4.018	0.5022	1.28192	P24941	P24941
2i3i_v	3.684	0.4605	1.63084	Q96CA5	Q96CA5	1cbs_v	5.776	0.5776	1.27095	P29373	P29373
1ln3_v	4.483	0.4075	1.73406	Q9UKL6	Q9UKL6	1khb_v	4.171	0.3208	1.18876	P35558	P35558
1dkf_v	3.758	0.4698	1.02322	RARA_HUMAN	P10276	1it6_v	4.312	0.3317	1.45307	P36873	P36873
1fd0_v	3.826	0.4783	1.06017	RARG_HUMAN	P13631	1r5l_v	4.935	0.4113	1.4463	P49638	P49638
1h9u_v	4.882	0.4882	1.74443	RXRB_HUMAN	P28702	2i6b_v	4.073	0.6788	1.96125	P55263	P55263
1irj_v	4.171	0.5958	2.84891	S10A9_HUMAN	P06702	1so2_v	3.549	0.7098	1.98421	PDE3B_HUMAN	Q13370
1l9n_v	4.686	0.5858	2.1097	TGM3_HUMAN	Q08188	1y2j_v	3.701	0.5287	1.42658	PDE4B_HUMAN	Q07343
1d4p_v	3.636	0.7272	1.4707	THRB_HUMAN	P00734	2bik_v	4.066	0.5809	2.2367	PIM1_HUMAN	P11309
1s19_v	4.103	0.5129	2.23664	VDR_HUMAN	P11473	1k7l_v	3.545	0.4431	1.15034	PPARA_HUMAN	Q07869
1u59_v	3.611	0.5159	1.07918	ZAP70_HUMAN	P43403	1e3k_v	3.807	0.6344	1.63347	PRGR_HUMAN	P06401
						1nwl_v	4.247	0.5308	3.2499	PTN1_HUMAN	P18031
						1mrq_v	3.358	0.6717	1.16263	Q04828	Q04828
						1ov4_v	3.75	0.7501	1.22216	Q06520	Q06520
						1sm2_v	3.808	0.6347	1.71253	Q08881	Q08881
						1wb0_v	3.768	0.471	2.1911	Q13231	Q13231
						1wbn_v	3.869	0.6448	2.01824	Q16539	Q16539
						2i3i_v	3.563	0.4453	1.43295	Q96CA5	Q96CA5
						1ln3_v	4.702	0.4275	2.13619	Q9UKL6	Q9UKL6
						1xap_v	4.393	0.4881	1.5866	RARB_HUMAN	P10826
						1fd0_v	4.238	0.5297	1.82161	RARG_HUMAN	P13631
						2g1s_v	3.565	0.4456	1.39856	RENI_HUMAN	P00797
						1s0x_v	5.12	0.512	2.73768	RORA_HUMAN	P35398
						1mv9_v	4.394	0.5493	1.92871	RXRA_HUMAN	P19793
						1h9u_v	4.502	0.4502	1.29992	RXRB_HUMAN	P28702
						1f5f_v	3.99	0.57	1.37892	SHBG_HUMAN	P04278
						1o43_v	3.717	0.413	1.40715	SRC_HUMAN	P12931
						1g3m_v	4.251	0.6073	1.91674	ST1E1_HUMAN	P49888
						1l9n_v	4.372	0.5465	1.65548	TGM3_HUMAN	Q08188
						1d4p_v	3.637	0.7273	1.57378	THRB_HUMAN	P00734
						2fxr_v	3.72	0.372	2.15273	TRYB2_HUMAN	P20231
						1rlb_v	5.673	0.5673	1.84498	TTHY_HUMAN	P02766
						1ju6_v	3.443	0.5738	1.46868	TYSY_HUMAN	P04818
						1s0z_v	4.772	0.4772	2.21984	VDR_HUMAN	P11473
						3ewh_v	3.77	0.377	1.16382	VEGFR2	P35968
						1j78_v	3.664	0.9161	1.50273	VTDB_HUMAN	P02774
						1u59_v	3.578	0.5112	1.088	ZAP70_HUMAN	P43403

Table S2. Protein coding targets for melanoma, breast and pancreatic cancer identified through the Genecards database.

**Gene Symbol**	**Uniprot ID**	**Melanoma**	**Breast cancer**	**Pancreatic cancer**
ABCB1	P08183	0	61.99821	48.88727
ABCB5	Q2M3G0	16.27885	0	0
ABCC1	P33527	0	74.57783	0

ABCG2	Q9UNQ0	0	66.28763	0
ABRAXAS1	Q6UWZ7	0	71.89273	0
ACD	Q96AP0	18.57563	0	0
ADAM12	O43184	16.94814	0	0
AGO2	Q9UKV8	0	56.15822	0
AIM2	O14862	22.30115		
AIP	O00170	0	58.33452	0
AKT1	P31749	36.43299	158.8691	105.2372
AKT2	P31751	0	0	46.93726
AKT3	Q9Y243	16.68421	0	0
ALK	Q9UM73	23.29008	141.4329	123.2215
AOPEP	Q8N6M6	0	94.39619	59.72108
APC	P25054	0	225.4557	200.8211
AR	P10275	17.14208	117.0743	70.13282
ARID1A	O14497		87.0449	75.22849
ATM	Q13315	35.17866	540.725	298.6444
ATR	Q13535	0	105.8571	86.8267
ATRX	P46100	0	56.88642	0
AURKA	O14965	0	62.05322	49.27502
AXIN2	Q9Y2T1	0	156.9984	133.9811
BAGE	Q13072	19.72662	0	0
BAP1	Q92560	72.98464	134.3389	114.476
BARD1	Q99728	18.92454	391.9306	198.726
BAX	Q07812	14.95992	72.81789	63.67015
BCAR1	P56945	0	60.45303	0
BCL2	P10415	20.20955	76.06853	61.89828
BIRC5	O15392	0	63.52531	57.91643
BLM	P54132	14.33827	145.1711	105.2115
BMPR1A	P36894		126.4149	105.6989
BRAF	P15056	114.3571	148.9661	139.6618
BRCA1	P38398	34.21312	619.5485	386.1326
BRCA2	P51587	40.9267	669.2446	421.4082
BRIP1	Q9BX63	20.29803	438.5371	220.8869
BRMS1	Q9HCU9	0	59.77565	0
BUB1B	O60566		67.89086	66.02465
C11orf65	Q8NCR3	333.3542	0	184.421
CARD11	Q9BXL7	14.06767	0	0
CASP3	P42574	16.54448	59.88084	54.11931
CASP8	Q14790	0	95.40147	61.136
CASR	P41180	0	0	58.95123
CCND1	P24385	33.32523	125.1836	94.0774
CCNE1	P24864	0	80.11903	64.10547
CD274	Q9NZQ7	29.17635	89.18533	80.58733
CD44	P16070	0	65.25496	53.85272
CD63	P08962	16.54162	0	0

CDC73	Q6P1J9	0	94.13683	71.20262
CDH1	P12830	18.31217	333.7174	188.5526
CDK12	Q9NYV4	0	79.27988	59.59361
CDK4	P11802	134.6689	139.6325	109.0762
CDK6	Q00534	27.17523	68.12589	54.0983
CDKN1A	P38936	18.41771	65.34682	58.9023
CDKN1B	P46527	0	109.5138	97.88589
CDKN1C	P49918	0	0	46.64406
CDKN2A	Q8N726	152.0502	160.3636	179.0295
CDKN2B	P42772	31.04669	78.11681	64.87206
CEL	P19835	0	0	51.27727
CFTR	P13569	0	0	86.86888
CHEK1	O14757	19.49309	89.09564	73.45135
CHEK2	O96017	30.44474	436.5323	230.9112
CPA1	P15085	55.06822	0	0
CPA1	P15085			
CREB1	P16220	14.57958	0	0
CREBBP	Q92793	0	63.06053	0
CSPG4	Q6UVK1	16.96574	0	0
CTLA4	P16410	28.29126	0	52.54491
CTNNA1	P35221	0	158.9213	103.3994
CTNNB1	P35222	33.66005	136.187	103.8214
CTRC	Q99895	56.5728	0	0
CXCL1	P09341	15.54573	0	0
CXCL8	P10145	0	0	49.14671
CXCR4	P61073	16.63083	63.89609	59.92258
CYP17A1	P05093	0	73.65149	0
CYP19A1	P11511	0	70.43009	0
CYSLTR2	Q9NS75	22.93747	0	0
DCT	P40126	16.68673	0	0
DCTN5	Q9BTE1	0	65.36148	0
DDB2	Q92466	22.839	0	0
DDR1	Q08345	17.12825	0	0
DICER1	Q9UPY3	14.35246	166.2254	138.3828
DLC1	Q96QB1	0	0	50.05437
DNAH1	Q9P2D7	19.52285	0	0
DNMT3A	Q9Y6K1	18.5924	0	0
EGF	P01133	0	0	46.38826
EGFR	P00533	30.04058	235.1702	202.6057
EIF1AX	P47813	27.17298	0	0
EP300	Q09472	0	83.20406	62.86908
EPCAM	P16422	18.38975	136.7251	114.7221
ERBB2	P04626	26.10611	253.5554	152.4754
ERBB3	P21860	0	92.24663	61.67943
ERBB4	Q15303	20.97177	84.54987	20.97177

ERCC1	P07992	0	60.13597	46.644
ERCC1	P07992	0	0	46.644
ERCC2	P18074	27.39098	91.23955	58.46856
ERCC3	P19447	21.26401	0	0
ERCC4	Q92889	21.09489	61.21636	59.35064
ERCC5	P28715	21.23018	62.09228	0
ESR1	P03372	0	179.6297	83.75999
EWSR1	Q01844	14.76491	0	0
EZH2	Q15910	18.37308	76.20058	57.33052
FANCA	O15360	20.54848	79.48431	65.06921
FANCC	Q00597	0	127.4469	99.9629
FANCD2	Q9BXW9	0	91.97935	69.12874
FANCE	Q9HB96	0	66.71062	54.4249
FANCF	Q9NPI8		63.85669	49.92748
FANCG	O15287	0	65.24105	58.91915
FANCI	Q9NVI1	0	57.72392	0
FANCL	Q9NW38	0	62.97609	51.43479
FANCM	Q8IYD8	0	96.54688	59.26799
FAS	P25445	49.02888	0	0
FASLG	P48023	0	0	52.00333
FBN1	P35555	15.5224	0	0
FBXW7	Q969H0	0	75.31535	53.42648
FGFR1	P11362	20.09338	94.52943	74.58968
FGFR2	P21802	17.9761	117.1885	91.3456
FGFR3	P22607	17.85253	100.1134	87.63348
FGFR4	P22455			
FGFR4	P22455	0	0	52.75244
FH	P07954	0	109.0949	86.21909
FHIT	P49789	0	61.13811	0
FLCN	Q8NFG4	0	99.76791	88.96972
FOXA1	P55317	0	73.46141	47.10125
GALNT12	Q8IXK2	0	59.05445	0
GATA3	P23771	0	82.13905	0
GATA6	Q92908	0	0	54.16615
GLI1	P08151	0	0	50.08908
GNA11	P29992	44.20374	0	0
GNAQ	P50148	47.94426	0	0
GNAS	P84996	17.52957	0	64.97181
GREM1	O60565		70.33272	57.8306
HIF1A	Q16665	17.18123	102.7234	79.40504
HMMR	O75330	0	69.11738	0
HNF1A	P20823	0	60.51448	53.31181
HNF1B	P35680	0	0	52.66565
HOXB13	Q92826	15.99552	102.1739	96.11382
HRAS	P01112	36.13221	107.2363	95.72232

IDH1	O75874	17.29647		59.3023
IDH2	P48735	16.30988	0	52.5554
IFNA1	P01562	15.84809	0	0
IFNG	P01579	16.35751	0	46.15404
IGF1	P05019	0	56.03579	0
IGF1R	P08069	0	63.27194	51.5332
IGF2	P01344	0	0	49.31522
IL10	P22301	0	0	46.48979
IL1B	P01584	0	63.54253	58.58881
IL2	P60568	22.84421	67.66321	0
IL24	Q13007	16.97787	0	0
IL6	P05231	14.10708	89.06818	75.90004
INS	P01308	51.69153	0	0
ITCH	Q96J02	16.30172	0	0
JAK2	O60674	0	58.34967	48.33041
JUN	P05412	0	76.44429	48.76753
KDR	P35968	14.20131	0	50.78141
KIT	P10721	39.22547	131.6181	114.9593
KLC1	Q07866	20.113	0	0
KMT2D	O14686	0	64.53993	0
KRAS	P01116	28.76685	148.8261	149.8408
LZTR1	Q8N653	0	86.48321	74.82753
MAGEA1	P43355	29.17655	0	0
MAGEA10	P43363	14.46469	0	0
MAGEA11	P43364	14.82496	0	0
MAGEA12	P43365	14.8557	0	0
MAGEA2	P43356	14.05901	0	0
MAGEA3	P43357	31.49519	0	0
MAGEA4	P43358	15.89692	0	0
MAGEA6	P43360	14.97222	0	0
MAGEB2	O15479	15.23348	0	0
MAGEC1	O60732	14.20457	0	0
MAGED1	Q9Y5V3	14.71943	0	0
MAP2K1	Q02750	33.07936	76.6552	57.37616
MAP2K2	P36507	24.57735	0	0
MAP2K4	P45985	0	55.64134	0
MAP3K1	Q13233	0	70.30081	0
MAPK1	P28482	15.93836	59.13753	53.1916
MAX	P61244		63.57494	51.91521
MBD4	O95243	27.35341	0	0
MC1R	Q01726	96.22695	0	0
MCAM	P43121	30.07995	0	0
MDM2	Q00987	21.04499	104.5988	73.26064
MELTF	P08582	17.56304	0	0
MEN1	O00255	0	120.0346	124.3247

MET	P08581	26.99107	149.8975	137.9787
MGMT	P16455	31.88171	0	48.18745
MIA	Q16674	27.1528	0	0
MITF	O75030	111.9895	67.7654	62.32529
MLANA	Q16655	37.53565	0	0
MLH1	P40692	27.90901	255.1807	213.9204
MLH3	Q9UHC1	0	85.57126	75.00493
MMP2	P08253	17.84158	57.29705	53.47307
MMP9	P14780	0	57.16981	53.52632
MRE11	P49959	0	139.8256	108.2362
MSH2	P43246	26.57988	260.688	214.414
MSH3	P20585		136.39	107.0452
MSH6	P52701	23.51069	261.6199	215.9307
MT-CYB	P00156	0	74.29556	0
MTA1	Q13330	0	60.99865	0
MTOR	P42345	21.42716	114.1116	84.818
MUC1	P15941	0	74.27483	62.47353
MUC16	Q8WXI7	0	78.55771	60.7976
MUTYH	Q9UIF7	0	157.8283	118.2175
MYC	P01106	28.64962	121.1917	92.71548
NBN	O60934	29.76358	173.0476	128.1623
NCOR1	O75376	0	58.25284	0
NF1	P21359	35.10582	185.0258	159.227
NF2	P35240	17.44	90.633	70.74871
NFE2L2	Q16236	0	56.7468	52.9183
NGFR	P08138	14.05761	0	0
NOTCH1	P46531	0	73.66273	80.93017
NPHP3	Q7Z494	0	0	52.22565
NRAS	P01111	55.36802	93.14447	80.23615
NRG1	Q02297	18.88752	0	0
NTHL1	P78549	0	100.0281	72.67387
NTRK1	P04629	21.47467	72.16765	52.51338
NTRK2	Q16620	16.55516	58.64225	0
NTRK3	Q16288	17.43724	62.57567	0
OCA2	Q04671	17.8083	0	0
PALB2	Q86YC2	28.05481	482.183	290.7933
PALLD	Q8WX93	0	0	107.719
PARP1	P09874	16.33894	59.09624	0
PBRM1	Q86U86	47.07866	0	0
PDCD1	Q15116	32.99334	61.98205	58.45397
PDGFRA	P16234	18.68095	111.3715	95.18822
PDX1	P52945	0	0	79.78531
PGR	P06401	0	67.63525	0
PHB1	P35232	0	62.03972	0
PHOX2B	Q99453	0	55.80176	47.53078

PIK3CA	P42336	32.99834	178.3652	124.5517
PIK3CG	P48736	14.10984	0	46.81896
PIK3R1	P27986	0	77.57992	51.44062
PITX1	P78337	14.97883	0	0
PLCB4	Q15147	28.64774	0	0
PMEL	P40967	38.67237	0	0
PMS1	P54277	0	84.08138	66.60274
PMS2	P54278	23.71358	216.9924	177.8862
PNLIP	P16233	0	0	62.76545
POLD1	P28340	0	178.3819	158.7388
POLE	Q07864	0	173.5119	138.6515
POT1	Q9NUX5	40.54015	87.17026	76.65765
PPARG	P37231	0	59.1496	56.95573
PPM1D	O15297	0	89.51253	0
PRAME	P78395	29.47854	0	0
PRKAR1A	P10644	0	80.61785	66.34778
PRKDC	P78527	0	65.75308	0
PRSS1	P07477	0	0	83.89069
PRSS2	P07478	0	0	48.25429
PTCH1	Q13635	20.35585	137.8223	20.35585
PTEN	P60484	56.95153	227.3403	177.504
PTF1A	Q7RTS3	0	0	55.57148
PTGS2	P35354	0	65.23264	59.41718
PTK2	Q05397	16.17494	56.51371	50.29196
PTK6	Q13882	0	57.23638	0
RABL3	Q5HYI8	48.63616	0	0
RAD50	Q92878	0	187.334	141.1701
RAD51	Q06609	21.31466	114.9851	78.67349
RAD51B	O15315		84.98767	59.95802
RAD51C	O43502	0	197.4842	126.4152
RAD51D	O75771	0	209.7909	128.6326
RAD54L	Q92698	0	86.62517	57.16557
RAF1	P04049	19.91864	0	46.03881
RB1	P06400	33.92296	145.2176	124.97
RB1CC1	Q8TDY2	0	61.76735	0
RECQL	P46063	0	73.41366	0
RECQL4	O94761	15.37856	84.37447	54.57122
RET	P07949	26.23742	152.3895	124.0837
RINT1	Q6NUQ1	0	59.28776	0
RNF43	Q68DV7	0	65.03284	70.78381
ROS1	P08922	17.0291	0	0
RUNX1	Q01196	0	103.314	88.00754
S100B	P04271	22.13725	0	0
SDHA	P31040	0	114.4416	88.05478
SDHAF2	Q9NX18	0	68.09451	53.22041

SDHB	P21912	20.31821	107.2475	78.89915
SDHC	Q99643	0	92.06057	63.98571
SDHD	O14521	15.68474	91.51993	65.49953
SEPTIN9	Q9UHD8	0	57.39552	0
SF3B1	O75533	36.77667	0	0
SHC1	P29353	0	66.71969	0
SLC45A2	Q9UMX9	32.68852	0	0
SLC67A1	Q96BI1	0	55.71971	0
SLX4	Q8IY92	0	65.41164	0
SMAD2	Q15796	0	0	49.19935
SMAD3	P84022	0	63.88618	53.60629
SMAD4	Q13485	0	148.4793	172.446
SMARCA4	P51532	18.51728	142.8995	119.2983
SMARCB1	Q12824	15.06228	82.00491	70.71309
SMARCE1	Q969G3	0	58.13596	46.25734
SMO	Q99835	15.86069	59.14577	56.03445
SOD2	P04179	0	0	48.52961
SOX10	P56693	22.80516	0	0
SOX9	P48436	0	0	49.23303
SPINK1	P00995	0	0	100.4693
SPRED1	Q7Z699	14.26086	0	0
SRC	P12931	22.13801	84.69838	60.55379
STAT1	P42224	0	68.60042	46.21427
STAT3	P40763	21.44246	94.96027	87.08474
STK11	Q15831	59.1829	171.8037	163.5375
SUFU	Q9UMX1	17.11469	69.41646	60.20163
TERF1	P54274	18.93765	0	0
TERF2	Q15554	18.72734	0	0
TERF2IP	Q9NYB0	29.48967	0	0
TERT	O14746	63.64605	121.8115	102.2655
TFF1	P04155	0	82.02419	46.98759
TGFB1	P01137	15.46024	69.15884	74.70341
TGFBR1	P36897	0	57.81929	0
TGFBR2	P37173	0	97.65627	85.25002
TINCR	A0A2R8Y7D0	14.72212	0	0
TMEM127	O75204	0	73.61097	58.63094
TNF	P01375	0	0	54.04094
TNFSF10	P50591	0	55.65212	50.4925
TP53	P04637	56.49036	310.1178	256.0034
TP63	Q9H3D4	0	58.05535	0
TRB	P0DSE2	0	0	56.39172
TRPM1	Q7Z4N2	14.27048	0	0
TSC1	Q92574	0	122.9979	110.1016
TSC2	P49815	0	144.9835	141.779
TSPAN31	Q12999	47.70525	0	0

TUG1	A0A6I8PU40	17.4294	73.17677	57.50064
TYR	P14679	44.68463	0	0
TYRP1	P17643	25.51834	0	0
VEGFA	P15692	18.23701	81.06743	74.63884
VHL	P40337	0	97.47833	108.4926
WEE1	P30291	0	57.95288	0
WRN	Q14191	23.40831	65.2076	47.10989
WT1	P19544	0	74.02679	61.4692
XPA	P23025	23.05297	0	0
XPC	Q01831	24.8467	58.64527	0
XRCC1	P18887	0	56.1701	0
XRCC2	O43543	0	100.3117	64.46467
XRCC3	O43542	18.6926	79.48451	0

Table S3. Pharmacologically predicted common targets of Bet and AP1–5 overlapping with cancer-specific targets

Compound	Gene symbol	UniProt ID	Fit Score	z score	Cancer Relevance (GeneCards score)	PDB ID
Bet	AR	P10275	4.092	1.52027	17.14 (melanoma), 117.07 (breast cancer), 70.13 (pancreatic cancer)	1gs4
AP5	AURKA	O14965	3.287	1.27567	62.10 (breast cancer), 48.28 (pancreatic cancer)	1w1g
AP2	BRAF	P15056	3.644	2.21447	114.36 (melanoma), 148.97 (breast cancer), 139.66 (pancreatic cancer)	2fb8
AP5	CHEK1	O14757	3.878	1.59164	19.49 (melanoma), 89.1 (breast cancer), 73.45 (pancreatic cancer)	2e9v
Bet	CHEK1	O14757	3.464	1.08672	19.49 (melanoma), 89.1 (breast cancer), 73.45 (pancreatic cancer)	2e9v
AP1	EGFR	P00533	3.385	1.33528	30.04 (melanoma), 235.17 (breast cancer), 202.61 (pancreatic cancer)	2ity
AP3	EGFR	P00533	3.474	1.58572	30.04 (melanoma), 235.17 (breast cancer), 202.61 (pancreatic cancer)	1m17
AP4	EGFR	P00533	3.702	2.17505	30.04 (melanoma), 235.17 (breast cancer), 202.61 (pancreatic cancer)	1m17
AP3	ERBB4	Q15303	3.599	1.26754	20.97 (melanoma), 84.55 (breast cancer), 65.60 (pancreatic cancer)	3bbt
AP1	ESR1	P03372	3.33	1.06277	179.63 (breast cancer), 83.76 (pancreatic cancer)	1g50
AP3	ESR1	P03372	3.469	1.21776	179.63 (breast cancer), 83.76 (pancreatic cancer)	1qkt
AP4	ESR1	P03372	3.619	1.68241	179.63 (breast cancer), 83.76 (pancreatic cancer)	1qkt
AP5	ESR1	P03372	3.931	1.98811	179.63 (breast cancer), 83.76 (pancreatic cancer)	1xp9
AP1	FGFR1	P11362	3.474	1.48816	20.10 (melanoma), 94.53 (breast cancer), 74.59 (pancreatic cancer)	1fgi
AP2	FGFR1	P11362	3.5	1.70508	20.10 (melanoma), 94.53 (breast cancer), 74.59 (pancreatic cancer)	1fgi
AP4	FGFR1	P11362	3.25	1.16466	20.10 (melanoma), 94.53 (breast cancer), 74.59 (pancreatic cancer)	1fgi
AP1	IGF1	P05019	3.627	1.55824	56.04 (breast cancer)	1gzr
AP2	IGF1	P05019	3.656	1.85757	56.04 (breast cancer)	1gzr
AP3	IGF1	P05019	3.604	1.5604	56.04 (breast cancer)	1gzr
AP4	IGF1	P05019	3.373	1.07644	56.04 (breast cancer)	1gzr
AP5	IGF1	P05019	3.505	1.55886	56.04 (breast cancer)	1gzr
AP1	IL2	P60568	3.735	1.29862	22.84 (melanoma), 67.66 (breast cancer)	1m48
AP2	IL2	P60568	3.958	1.95251	22.84 (melanoma), 67.66 (breast cancer)	1m48
AP3	IL2	P60568	3.946	1.76739	22.84 (melanoma), 67.66 (breast cancer)	1m48
AP4	IL2	P60568	3.614	1.18605	22.84 (melanoma), 67.66 (breast cancer)	1m48
AP5	IL2	P60568	3.825	1.56363	22.84 (melanoma), 67.66 (breast cancer)	1qvn

Bet	IL2	P60568	3.609	1.43668	22.84 (melanoma), 67.66 (breast cancer)	1qvn
AP2	JAK2	O60674	3.915	1.58118	58.35 (breast cancer), 48.33 (pancreatic cancer)	2b7a
AP3	JAK2	O60674	3.735	1.07499	58.35 (breast cancer),48.33 (pancreatic cancer)	2b7a
AP4	JAK2	O60674	3.96	1.60342	58.35 (breast cancer), 48.33 (pancreatic cancer)	2b7a
AP5	KDR	P35968	3.77	1.16382	14.20 (melanoma), 50.78 (pancreatic cancer)	3ewh
AP1	MAP2K1	Q02750	5.044	1.87012	33.8 (melanoma), 76.66 (breast cancer), 57.37 (pancreatic cancer)	1s9j
AP2	MAP2K1	Q02750	4.495	1.33419	33.8 (melanoma), 76.66 (breast cancer), 57.37 (pancreatic cancer)	1s9j
AP5	MAP2K1	Q02750	4.45	1.31901	33.8 (melanoma), 76.66 (breast cancer), 57.37 (pancreatic cancer)	1s9j
AP2	MDM2	Q00987	3.611	1.97452	21.04 (melanoma), 104.60 (breast cancer), 73.26 (pancreatic cancer)	1rv1
AP3	MDM2	Q00987	3.336	1.06455	21.04 (melanoma), 104.60 (breast cancer), 73.26 (pancreatic cancer)	1rv1
AP5	MDM2	Q00987	3.878	1.62808	21.04 (melanoma), 104.60 (breast cancer), 73.26 (pancreatic cancer)	1t4e
Bet	MDM2	Q00987	3.63	1.4352	21.04 (melanoma), 104.60 (breast cancer), 73.26 (pancreatic cancer)	1t4e
AP1	MET	P08581	3.67	1.03888	27.00 (melanoma), 149.90 (breast cancer), 137.98 (pancreatic cancer)	1r0p
AP2	MET	P08581	3.575	1.01994	27.00 (melanoma), 149.90 (breast cancer), 137.98 (pancreatic cancer)	1r04
AP1	MMP2	P08253	3.632	1.17111	17.84 (melanoma), 57.30 (breast cancer), 53.47 (pancreatic cancer)	1hov
AP2	MMP2	P08253	3.951	1.97463	17.84 (melanoma), 57.30 (breast cancer), 53.47 (pancreatic cancer)	1hov
AP3	MMP2	P08253	3.707	1.3588	17.84 (melanoma), 57.30 (breast cancer), 53.47 (pancreatic cancer)	1hov
AP4	MMP2	P08253	3.763	1.54634	17.84 (melanoma), 57.30 (breast cancer), 53.47 (pancreatic cancer)	1hov
AP5	MMP2	P08253	3.627	1.40374	17.84 (melanoma), 57.30 (breast cancer), 53.47 (pancreatic cancer)	1hov
Bet	PARP1	P09874	3.693	2.31845	16.34 (melanoma), 59.10 (breast cancer)	1wok
AP2	PGR	P06401	3.835	2.50749	67.64 (breast cancer)	1a28
AP5	PGR	P06401	3.807	1.63347	67.64 (breast cancer)	1e3k
Bet	PGR	P06401	4.098	2.61908	67.64 (breast cancer)	1e3k
AP1	PIK3R1	P27986	3.703	1.4387	77.58 (breast cancer), 51.44 (pancreatic cancer)	1pic
AP4	PIK3R1	P27986	3.649	1.46543	77.58 (breast cancer), 51.44 (pancreatic cancer)	1pic
Bet	PIK3R1	P27986	3.614	1.92555	77.58 (breast cancer), 51.44 (pancreatic cancer)	1pic
Bet	PPARG	P37231	3.558	1.48439	59.1496 (breast cancer), 56.96 (pancreatic cancer)	1rdt
AP4	SRC	P12931	3.196	1.05598	12.14 (melanoma), 84.70 (breast cancer), 60.55 (pancreatic cancer)	2h8h
AP5	SRC	P12931	3.717	1.40715	12.14 (melanoma), 84.70 (breast cancer), 60.55 (pancreatic cancer)	1o43
AP4	TGFBR1	P36897	3.568	2.03912	57.82 (breast cancer)	1vjy

Table S4. STRING database interaction scores for cancer-associated predicted targets

#node1	node2	homology	coexpression	experimentally_determined_interaction	database_annotated	automated_textmining	combined_score
ESR1	MDM2	0	0.042	0.785	0	0.762	0.946
ESR1	PGR	0.673	0.24	0.771	0.7	0.56	0.974
ESR1	IGF1	0	0.062	0	0	0.964	0.964
FGFR1	IGF1	0	0.042	0	0.65	0.507	0.82
IGF1	MMP2	0	0.082	0	0.4	0.682	0.809
IGF1	JAK2	0	0	0.063	0	0.826	0.829
IGF1	FGFR1	0	0.042	0	0.65	0.507	0.82
IGF1	ESR1	0	0.062	0	0	0.964	0.964
IL2	JAK2	0	0.042	0	0.65	0.715	0.896
JAK2	IL2	0	0.042	0	0.65	0.715	0.896

JAK2	MAP2K1	0.601	0.087	0.161	0.4	0.533	0.757
JAK2	IGF1	0	0	0.063	0	0.826	0.829
MAP2K1	JAK2	0.601	0.087	0.161	0.4	0.533	0.757
MDM2	ESR1	0	0.042	0.785	0	0.762	0.946
MMP2	IGF1	0	0.082	0	0.4	0.682	0.809
PGR	ESR1	0.673	0.24	0.771	0.7	0.56	0.974

Table S5. String enrichment analysis results

**Term description**	**Observed gene count**	**False discovery rate**	**Matching proteins**
Wiki Pathways			
Focal adhesion: PI3K-Akt-mTOR-signaling pathway	6	8.58×10^{-7}	IL2,MDM2,MAP2K1,JAK2,IGF1,FGFR1
PI3K-Akt signaling pathway	6	8.58×10^{-7}	IL2,MDM2,MAP2K1,JAK2,IGF1,FGFR1
Breast cancer pathway	5	1.03×10^{-6}	MAP2K1,PGR,IGF1,FGFR1,ESR1
Mammary gland development pathway - Pregnancy and lactation (Stage 3 of 4)	3	9.11×10^{-5}	PGR,JAK2,ESR1
IL-5 signaling pathway	3	9.80×10^{-5}	IL2,MAP2K1,JAK2
Bladder cancer	3	9.80×10^{-5}	MMP2,MDM2,MAP2K1
Malignant pleural mesothelioma	5	9.80×10^{-5}	MMP2,MDM2,MAP2K1,IGF1,FGFR1
AGE/RAGE pathway	3	0.00033	MMP2,MAP2K1,JAK2
Leptin signaling pathway	3	0.00044	MAP2K1,JAK2,ESR1
Glioblastoma signaling pathways	3	0.0005	MDM2,MAP2K1,FGFR1
KEGG Pathways			
Pathways in cancer	8	6.89×10^{-10}	MMP2,IL2,MDM2,MAP2K1,JAK2,IGF1,FGFR1, ESR1
Proteoglycans in cancer	6	1.39×10^{-8}	MMP2,MDM2,MAP2K1,IGF1,FGFR1,ESR1
Endocrine resistance	5	4.01×10^{-8}	MMP2,MDM2,MAP2K1,IGF1,ESR1
PI3K-Akt signaling pathway	6	2.21×10^{-7}	IL2,MDM2,MAP2K1,JAK2,IGF1,FGFR1
Breast cancer	5	2.21×10^{-7}	MAP2K1,PGR,IGF1,FGFR1,ESR1
Melanoma	4	1.42×10^{-6}	MDM2,MAP2K1,IGF1,FGFR1
Prostate cancer	4	3.85×10^{-6}	MDM2,MAP2K1,IGF1,FGFR1
Estrogen signaling pathway	4	1.15×10^{-5}	MMP2,MAP2K1,PGR,ESR1
Signaling pathways regulating pluripotency of stem cells	4	1.29×10^{-5}	MAP2K1,JAK2,IGF1,FGFR1
Bladder cancer	3	2.71×10^{-5}	MMP2,MDM2,MAP2K1

Table S6. Enrichr enrichment analysis results

**Pathway**	**Overlap**	**Adjusted P-value**	**Genes**
Wiki pathways 2024 human			
Cancer pathways WP5434	8/507	2.97×10^{-10}	MAP2K1;MMP2;MDM2;IGF1;JAK2;ESR1;IL2;FGFR1
Focal adhesion PI3K Akt mTOR signaling WP3932	6/303	9.72×10^{-8}	MAP2K1;MDM2;IGF1;JAK2;IL2;FGFR1
PI3K Akt signaling WP4172	6/338	1.25×10^{-7}	MAP2K1;MDM2;IGF1;JAK2;IL2;FGFR1
Breast cancer pathway WP4262	5/154	1.63×10^{-7}	MAP2K1;PGR;IGF1;ESR1;FGFR1
Mammary gland development pregnancy & lactation stage 3/4 WP2817	3/33	1.43×10^{-5}	PGR;JAK2;ESR1
IL5 signaling WP127	3/37	1.61×10^{-5}	MAP2K1;JAK2;IL2
Pleural mesothelioma WP5087	5/437	1.61×10^{-5}	MAP2K1;MMP2;MDM2;IGF1;FGFR1
Bladder cancer WP2828	3/40	1.61×10^{-5}	MAP2K1;MMP2;MDM2
AGE RAGE pathway WP2324	3/66	6.60×10^{-5}	MAP2K1;MMP2;JAK2
Leptin signaling WP2034	3/71	7.41×10^{-5}	MAP2K1;JAK2;ESR1
Elsevier pathway collection			
Endometrioid endometrial cancer	6/92	2.53×10^{-10}	MAP2K1;MDM2;PGR;IGF1;JAK2;ESR1

ESR1/ERBB positive luminal breast cancer	6/93	2.53×10^{-10}	MAP2K1;MDM2;PGR;IGF1;JAK2;ESR1
Endometrial cancer	6/106	3.77×10^{-10}	MAP2K1;MDM2;PGR;IGF1;JAK2;ESR1
Melanoma	6/145	1.56×10^{-9}	MAP2K1;MMP2;MDM2;IGF1;JAK2;FGFR1
ESR1 signaling in breast cancer	5/51	1.56×10^{-9}	MAP2K1;MDM2;PGR;JAK2;ESR1
Proteins involved in breast cancer related to IGF1R/Akt signaling pathway	5/55	1.93×10^{-9}	MAP2K1;MMP2;MDM2;IGF1;JAK2
VEGFA dependent angiogenesis in cancer	5/62	3.07×10^{-9}	MAP2K1;PGR;IGF1;JAK2;ESR1
Proteins involved in breast cancer related to ESR1 signaling pathway	5/64	3.17×10^{-9}	MAP2K1;MMP2;MDM2;JAK2;ESR1
Proteins involved in endometriosis	6/256	2.66×10^{-8}	MMP2;PGR;IGF1;ESR1;IL2;FGFR1
Prostate Cancer	5/123	7.11×10^{-8}	MMP2;MDM2;IGF1;JAK2;FGFR1
KEGG 2024			
Pathways in cancer	8/525	2.58×10^{-10}	MAP2K1;MMP2;MDM2;IGF1;JAK2;ESR1;IL2;FGFR1
Proteoglycans in cancer	6/197	4.75×10^{-9}	MAP2K1;MMP2;MDM2;IGF1;ESR1;FGFR1
Breast cancer	5/146	8.25×10^{-8}	MAP2K1;PGR;IGF1;ESR1;FGFR1
PI3K-AKT signaling pathway	6/355	8.25×10^{-8}	MAP2K1;MDM2;IGF1;JAK2;IL2;FGFR1
Melanoma	4/72	$5.26E\times 10^{-7}$	MAP2K1;MDM2;IGF1;FGFR1
Prostate cancer	4/103	1.87×10^{-6}	MAP2K1;MDM2;IGF1;FGFR1
Estrogen signaling pathway	4/135	4.77×10^{-6}	MAP2K1;MMP2;PGR;ESR1
Signaling pathways regulating the pluripotency of stem cells	4/141	4.97×10^{-6}	MAP2K1;IGF1;JAK2;FGFR1
Bladder cancer	3/40	9.40×10^{-6}	MAP2K1;MMP2;MDM2
Chemical carcinogenesis- receptor activation	4/195	1.46×10^{-5}	MAP2K1;PGR;JAK2;ESR1

Table S7. Physicochemical and ADMET predictions for Bet and AP5 according to ADMETlab 3.0. MW: molecular weight; logP: octanol–water partition coefficient; logD: distribution coefficient; logS: aqueous solubility; TPSA: topological polar surface area; nRot: number of rotatable bonds; Fsp³: fraction of sp³ carbons; MCE-18: medicinal chemistry evolution score; NPscore: natural product-likeness score; Ro5: Lipinski's rule of five; QED: quantitative estimate of drug-likeness; PAINS: pan-assay interference alerts; ALARM NMR: assay for reactive molecules by nuclear magnetic resonance; Caco-2: intestinal permeability model; HIA: human intestinal absorption; P-gp: P-glycoprotein; F: oral bioavailability; BBB: blood–brain barrier; PPB: plasma protein binding; Fu: fraction unbound; VDss: volume of distribution; OATP1B1: organic anion transporting polypeptide 1B1; HLM: human liver microsomes; CYP: cytochrome P450; CL: plasma clearance; T½: half-life; hERG: human ether-à-go-go-related gene; DILI: drug-induced liver injury; AMES: Ames mutagenicity test; H-HT: hepatotoxicity; A549: human lung carcinoma cell line.

**Property**	**Optimal range**	**Betulin**	**AP5**
Physicochemical properties			
Molecular weight (Da)	100–600	442.4	724.4
logP	≤ 5.0	5.2	5.91
logD	−2 to 5	4.54	4.45
logS (aqueous solubility)	> −4 (optimal)	−5.48	−7.48
TPSA (Å²)	0–140	40.5	135.7
nRot (rotatable bonds)	0–11	2	12
Fsp³	≥ 0.42	0.933	0.789
MCE-18	≥ 45	100.8	170
NPscore	−5 to 5 (higher = NP-like)	3.23	1.68
Absorption			
Caco-2 permeability (log unit)	> −5.15	−5.04	−4.88
HIA	HIA− = well absorbed	HIA− (0.0)	HIA− (0.0)
P-gp substrate	Non-substrate preferred	0.908	0
P-gp inhibitor	Non-inhibitor preferred	0.001	0.81

F ≥ 20% bioavailability	Lower prob. = higher F	0.597	0.416
Distribution			
BBB penetration	BBB− preferred (anticancer)	0.997	0.04
PPB (%)	< 90%	93.20%	98.40%
Fu (fraction unbound, %)	> 5% middle range	6.99%	1.57%
VDss (L/kg)	0.04–20	−0.007	0.007
OATP1B1 inhibitor	Non-inhibitor preferred	0.96	1
Metabolism			
HLM stability	Stable >30 min (prob. → 0)	0.39	0
CYP2C19 substrate	Non-substrate preferred	0.998	0.404
CYP3A4 substrate	Non-substrate preferred	0.112	1
CYP2C9 substrate	Non-substrate preferred	0.041	0.805
CYP2B6 inhibitor	Non-inhibitor preferred	0.983	0.427
CYP2C8 inhibitor	Non-inhibitor preferred	0.964	0.99
Excretion			
Cl plasma (ml/min/kg)	5–15 moderate; <5 low	9.9	0.85
T½ (hours)	>4 h intermediate; >8 h long	0.72	1.43
Toxicity			
hERG blocker	< 0.5 preferred	0.143	0.187
DILI risk	< 0.5 preferred	0.378	1
AMES mutagenicity	< 0.5 preferred	0.468	0.199
Carcinogenicity	< 0.5 preferred	0.927	0.978
Genotoxicity	< 0.5 preferred	0.545	1
Nephrotoxicity	< 0.5 preferred	0.748	0.918
Skin sensitization	< 0.5 preferred	0.97	0.903
Hepatotoxicity (H-HT)	< 0.5 preferred	0.659	0.455
Hematotoxicity	< 0.5 preferred	0.608	0.169
A549 cytotoxicity	< 0.5 = non-cytotoxic	0.376	0.001
Rat oral acute toxicity	< 0.5 = low toxicity	0.28	0.094
